# Continuous evolution of compact protein degradation tags regulated by selective molecular glues

**DOI:** 10.1126/science.adk4422

**Published:** 2024-03-15

**Authors:** Jaron A. M. Mercer, Stephan J. DeCarlo, Shourya S. Roy Burman, Vedagopuram Sreekanth, Andrew T. Nelson, Moritz Hunkeler, Peter J. Chen, Katherine A. Donovan, Praveen Kokkonda, Praveen K. Tiwari, Veronika M. Shoba, Arghya Deb, Amit Choudhary, Eric S. Fischer, David R. Liu

**Affiliations:** 1 Merkin Institute of Transformative Technologies in Healthcare, Broad Institute of Harvard and MIT, Cambridge, MA 02142; 2 Department of Chemistry and Chemical Biology, Harvard University, Cambridge, MA 02138; 3 Howard Hughes Medical Institute, Harvard University, Cambridge, MA 02138; 4 Department of Cancer Biology, Dana-Farber Cancer Institute, Boston, MA 02215; 5 Department of Biological Chemistry and Molecular Pharmacology, Harvard Medical School, Boston, MA 02115; 6 Chemical Biology and Therapeutics Science, Broad Institute of Harvard and MIT, Cambridge, MA 02142; 7 Department of Medicine, Harvard Medical School, Boston, MA 02115; 8 Divisions of Renal Medicine and Engineering, Brigham and Women’s Hospital, Boston, MA 02115

## Abstract

Conditional protein degradation tags (degrons) are usually >100 amino acids or are triggered by small molecules with substantial off-targets, thwarting their use as specific modulators of endogenous protein levels. We developed a phage-assisted continuous evolution platform for molecular glue complexes (MG-PACE) and evolved a 36-amino acid zinc-finger (ZF) degron (SD40) that binds the ubiquitin ligase substrate receptor cereblon in complex with PT-179, an orthogonal thalidomide derivative. Endogenous proteins tagged in-frame with SD40 using prime editing are degraded by otherwise inert PT-179. Cryo-electron microscopy structures of SD40 in complex with ligand-bound cereblon revealed mechanistic insights into the molecular basis of SD40’s activity and specificity. Our efforts establish a system for continuous evolution of molecular glue complexes and ZF tags that overcome shortcomings associated with existing degrons.

## One Sentence Summary

Rapid evolution of molecular glue interfaces yields a compact small molecule-triggered degron with high target protein specificity.

## Introduction

Molecular glues are small molecules that induce binding of two proteins, enabling precise temporal control over proximity-driven biological processes (1-4). Thalidomide and its analogs pomalidomide and lenalidomide (IMiDs, immunomodulatory drugs) are molecular glues that bind cereblon (CRBN), the substrate receptor of the CRL4^CRBN^ E3 ubiquitin ligase, altering its substrate specificity to recognize protein neosubstrates that are ubiquitinated and subsequently degraded (5-7). CRBN•IMiD•neosubstrate ternary complexes contain an extended interface of protein–protein contacts, and IMiDs do not bind protein neosubstrates in the absence of CRBN (8-11). As a result, IMiDs elicit sigmoidal dose-response curves without the ‘hook effect’ characteristic of bivalent inducers of proximity (12, 13). IMiD-bound CRBN recognizes a small β-turn motif often found in zinc-finger (ZF) transcription factors. Appending this small β-turn motif, commonly called a ‘degron’, to other proteins makes the resulting fusion protein susceptible to IMiD-induced degradation (9, 14-16).

Small molecule-responsive protein tags are indispensable tools in chemical biology. Technologies for small molecule-induced degradation of target proteins include the dTAG system (17, 18), small-molecule-assisted shutoff (SMASh) (19), ligand-induced degradation (20), HaloProtacs (21, 22), and the auxin-inducible degron (23-25). These tags, however, are primarily limited to exogenous expression of fusion proteins due to their large size (108- to 304-amino acids) or require co-expression of non-native effector domains (23, 26). This requirement precludes the study of proteins expressed from their endogenous genomic loci at native expression levels under endogenous regulatory control. In contrast, some zinc-finger (ZF) motifs recognized by IMiD-bound CRBN are small enough to be, in theory, precisely inserted into a target gene in-frame using prime editing (27-30). However, IMiDs induce degradation of numerous off-target neosubstrates in human cells, limiting their utility as research tools (7, 9, 31, 32). An ideal degron tag would be small enough for precision in-frame installation via genome editing, respond to a cell-permeable small molecule with no off-target effects, and be invulnerable to the hook effect.

In this study, we developed a phage-assisted continuous evolution system (PACE) to reprogram molecular glue complexes and applied the system to evolve a small, 36-residue ZF domain that engages IMiD-bound human CRBN. We evolved this small degron in the presence of orthogonal IMiD derivatives bearing phthalimide-ring substitutions that disrupt binding to native protein neosubstrates and verified that these ‘bumped’ IMiD derivatives exhibit greatly reduced off-target activity in human cells compared to pomalidomide, a canonical IMiD. The evolved degron is small enough to be efficiently inserted in-frame into a target endogenous genomic site in human cells using prime editing for in-frame gene tagging. Human proteins tagged with the evolved degron are rapidly and potently degraded by PT-179, an orthogonal IMiD derivative. To overcome the fundamental limitation that IMiD-based degrons are inactive in rodents, we separately evolved ZF variants that engage mouse CRBN, enabling induced protein degradation in mouse cells. Finally, we obtained cryo-electron microscopy (cryo-EM) structures of the evolved zinc finger in complex with CRBN and bumped as well as canonical IMiD derivatives revealing an expanded interface of evolved interactions bridging the CRBN N-terminal and C-terminal domains. These findings collectively establish a continuous evolution platform for the rapid remodeling of molecular glue complexes, provide degrons that recognize new, highly specific molecular glues, and discover a neosubstrate-mediated mechanism of inducing CRBN to adopt its closed, putatively active conformation.

## Results

### A phage-assisted continuous evolution circuit for molecular glue complexes

**Figure 1.**
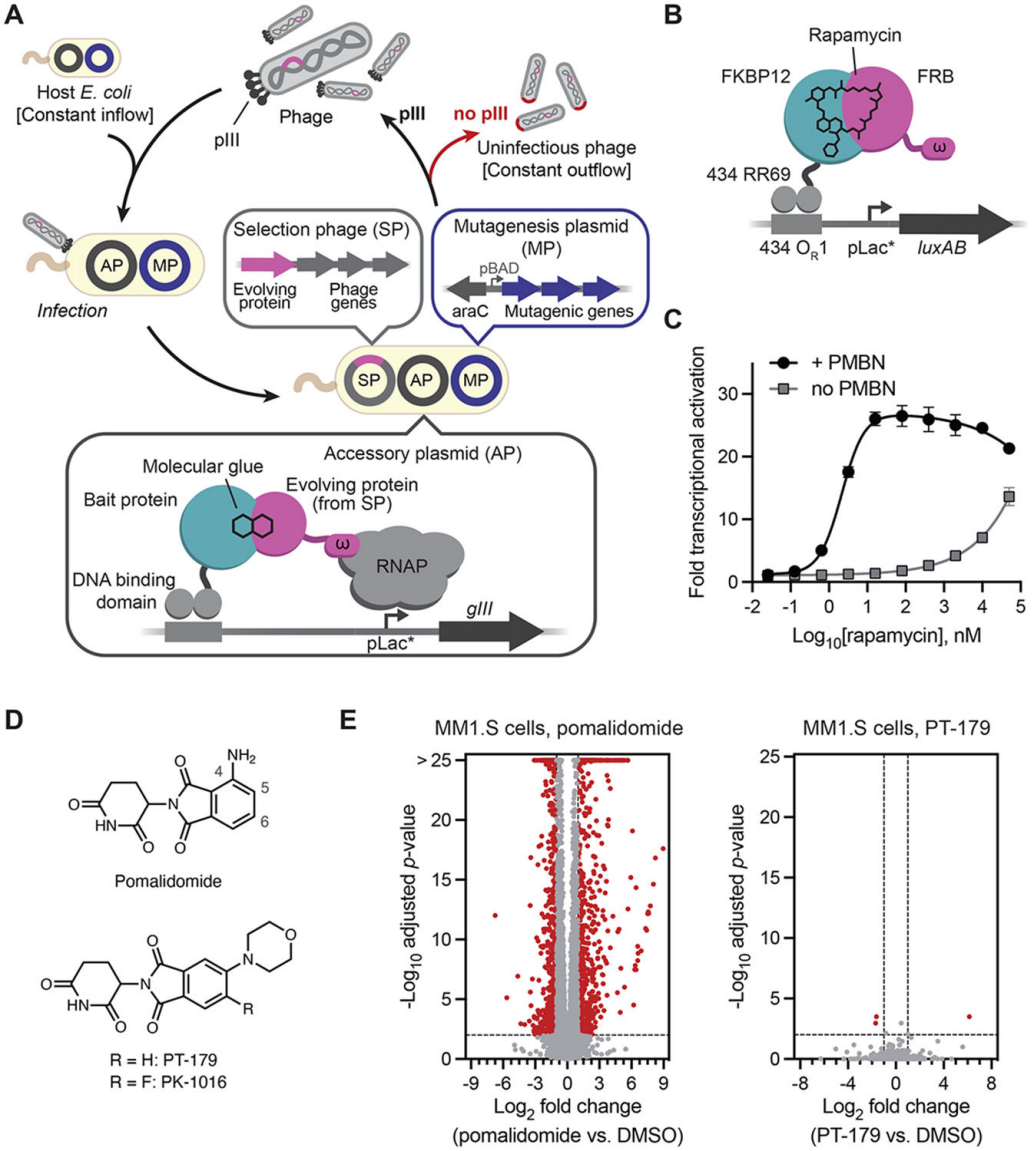
Phage-assisted continuous evolution circuit for molecular glue complexes (MG-PACE). (**A**) In PACE, the gene for a protein of interest (POI) is placed on a selection phage (SP) in place of the phage gene *gIII*, which encodes the phage coat protein pIII. Host cells harbor an accessory plasmid (AP) encoding a selection circuit that provides pIII and a mutagenesis plasmid (MP) that increases the incidence of mutation during SP replication. (**B**) Rapamycin-sensitive MG-PACE circuit. (**C**) Rapamycin-induced activation of the MG-PACE circuit (1 hr). (**D**) Pomalidomide and IMiD derivatives PT-179 and PK-1016. (**E**) Whole-transcriptome RNA-Seq of MM1.S cells treated with pomalidomide (1 μM, 48 hrs, left) or PT-179 (1 μM, 48 hrs, right). Values and error bars in (C) represent the mean and standard deviation of three replicates, each normalized to a control treatment with DMSO only. Values in (E) represent the mean of five replicates; *p*-values for each gene were calculated using the Wald test and adjusted using the Benjamini-Hochberg method (84).

Phage-assisted continuous evolution (PACE) harnesses the very short life cycle of the M13 *E. coli* bacteriophage (~10 minutes) to perform many generations of evolution in a short time period with minimal researcher intervention, accelerating laboratory evolution typically by >100-fold (Fig. 1A) (33, 34). Selection phage (SP) harbor all the genes necessary to produce progeny phage except for *gIII*, which encodes the phage coat protein pIII, and is replaced by a gene encoding the evolving protein of interest (POI). Host cells contain an accessory plasmid (AP) that provides pIII if the POI encoded in the SP has the desired activity. Selection pressure is applied by dilution with fresh host cells either continuously (PACE) or periodically (phage-assisted non-continuous evolution, PANCE) in fixed-volume vessels called lagoons. SP that fail to replicate faster than the rate of dilution wash out of the lagoon. Host cells also express a suite of proteins from a mutagenesis plasmid (MP) that increase the frequency of substitution mutations during SP replication (35). PACE and PANCE have been used to evolve a wide variety of proteins including polymerases (33, 36-38), proteases (39-41), protein-binding proteins (42-44), metabolic enzymes (45), DNA-binding proteins (46), and gene editing agents such as Cas nucleases (47, 48), base editors (49-53), prime editors (54) and TALE nucleases (55) with new or improved activities and specificities.

To apply continuous evolution to molecular glue complexes, we built on previous PACE selections for protein–DNA and protein–protein binding interactions using hybrid transcription activation circuits in *E. coli* (42, 47, 48, 55) to develop a PACE selection that links pIII expression to molecular glue ternary complex formation (MG-PACE). In the bacterial two-hybrid system, a specified protein–protein binding event recruits RNA polymerase to initiate transcription of a reporter gene (56, 57). We designed an initial selection system responsive to rapamycin, which induces dimerization of FKBP12 and FRB (FKBP12-rapamycin-binding fragment of mTOR) (58, 59). FKBP12 is anchored upstream of a pLac-derived promoter (pLac*) by fusion to the DNA binding protein RR69, a derivative of the 434 phage repressor (60). FRB is fused to the small ω-subunit of the *E. coli* RNA polymerase. Rapamycin-induced binding of FKBP12 and FRB recruits the full RNA polymerase to pLac*, driving expression of *gIII* or a luciferase reporter *luxAB* (Fig. 1B).

*E. coli* are impermeable to many small molecules (61), which could prevent activation of the MG-PACE circuit. Indeed, we detected luciferase expression only at high (>1μM) concentrations of rapamycin (Fig. 1C). Coadministration of polymyxin B nonapeptide (PMBN), a polycationic small molecule that permeabilizes the *E. coli* outer membrane (62), produced a robust rapamycin dose-response curve with a sub-nanomolar EC_50_ (Fig. 1C). A hook effect emerges at high concentrations, reflecting rapamycin's modest affinity for FRB without FKBP12 (K_D_ = 26 μM) (63, 64). These data suggest that under permeabilizing conditions, the MG-PACE circuit activates fast enough to reflect equilibrium binding dynamics of the molecular glue ternary complex. Collectively, these findings establish that the MG-PACE gene circuit links the affinity of a known molecular glue interaction to gene expression, and thus can serve as the basis of a PACE selection.

### Orthogonal IMiD derivatives with no detected off-target neosubstrates

IMiDs bind a hydrophobic pocket in the C-terminal domain of CRBN, placing their glutarimide ring within the pocket and leaving the phthalimide ring partially exposed (65). CRBN•IMiD neosubstrates engage both the exposed IMiD phthalimide ring and the surrounding CRBN protein surface (8-11). Recently, we synthesized a panel of IMiD derivatives featuring substituents at the phthalimide 4- and 5-positions to identify motifs that impede native neosubstrate engagement, thereby minimizing off-target protein degradation (66). In that study, we identified PT-179 (Fig. 1D) as a potential orthogonal IMiD derivative that does not induce degradation of known pomalidomide neosubstrates.

To thoroughly assess the potential of PT-179 for neosubstrate degradation in an unbiased, proteome-wide and transcriptome-wide manner, we treated human cells lines that are known to express neosubstrates of canonical IMiDs with either pomalidomide or PT-179 and analyzed the treated cells by global proteomic and transcriptomic analyses. In MOLT4 cells, an acute lymphoblastic leukemia line, pomalidomide induced significant downregulation of the previously identified neosubstrates IKZF1 (9) and ZFP91 (9, 31), while PT-179 did not significantly downregulate any detected proteins (no downregulation ≥ 2-fold at *p* ≤ 0.0001; fig. S1A). In KELLY cells, a neuroblastoma line, pomalidomide induced robust downregulation of the developmental transcription factor SALL4 (32) and ZFP91 (31), while PT-179 exhibited no significant degradation of detected proteins (fig. S1B,D,E). In HEK293T cells, pomalidomide induced degradation of ZFP91, while PT-179 did not downregulate any detected proteins (fig. S1C). In addition, we treated multiple myeloma MM.1S cells with both compounds and performed whole-transcriptome sequencing. Pomalidomide induced differential expression of 1,426 genes, reflecting degradation of key transcription and epigenetic regulators such as IKZF1/3 and ARID2 (Fig. 1E) (67). Treatment with PT-179 resulted in only three differentially expressed genes, a 475-fold decrease in the frequency of gene expression perturbation, consistent with very low neosubstrate activity (Fig. 1E). Taken together, these results demonstrate that PT-179 is an IMiD derivative with minimal endogenous neosubstrates in the tested cell types.

To verify that PT-179 still binds CRBN, we purified the DDB1•CRBN complex and measured its affinity for PT-179 by competitive fluorescence anisotropy (65). PT-179 binds CRBN with a dissociation constant (K_D_) of 587 nM, similar to pomalidomide (K_D_ = 500 nM, Fig. S2A-C). Finally, we performed competitive CRBN engagement assays by bioluminescence resonance energy transfer (BRET) from a CRBN–NanoLuc fusion to a fluorescent IMiD conjugate in human cells (68). These assays suggested that PT-179 engages CRBN in HEK293T and U2OS cells with 7-fold lower apparent potency than pomalidomide (fig. S2D), though we note that pomalidomide's apparent affinity for CRBN in cells is likely enhanced by neosubstrate recruitment since dissociation constants are lower for CRBN•IMiD•neosubstrate ternary complexes than for CRBN•IMiD binary complexes (fig S2C, fig. S15C,D).

### Evolution of ZF degrons that engage CRBN bound to orthogonal IMiD derivatives

**Figure 2.**
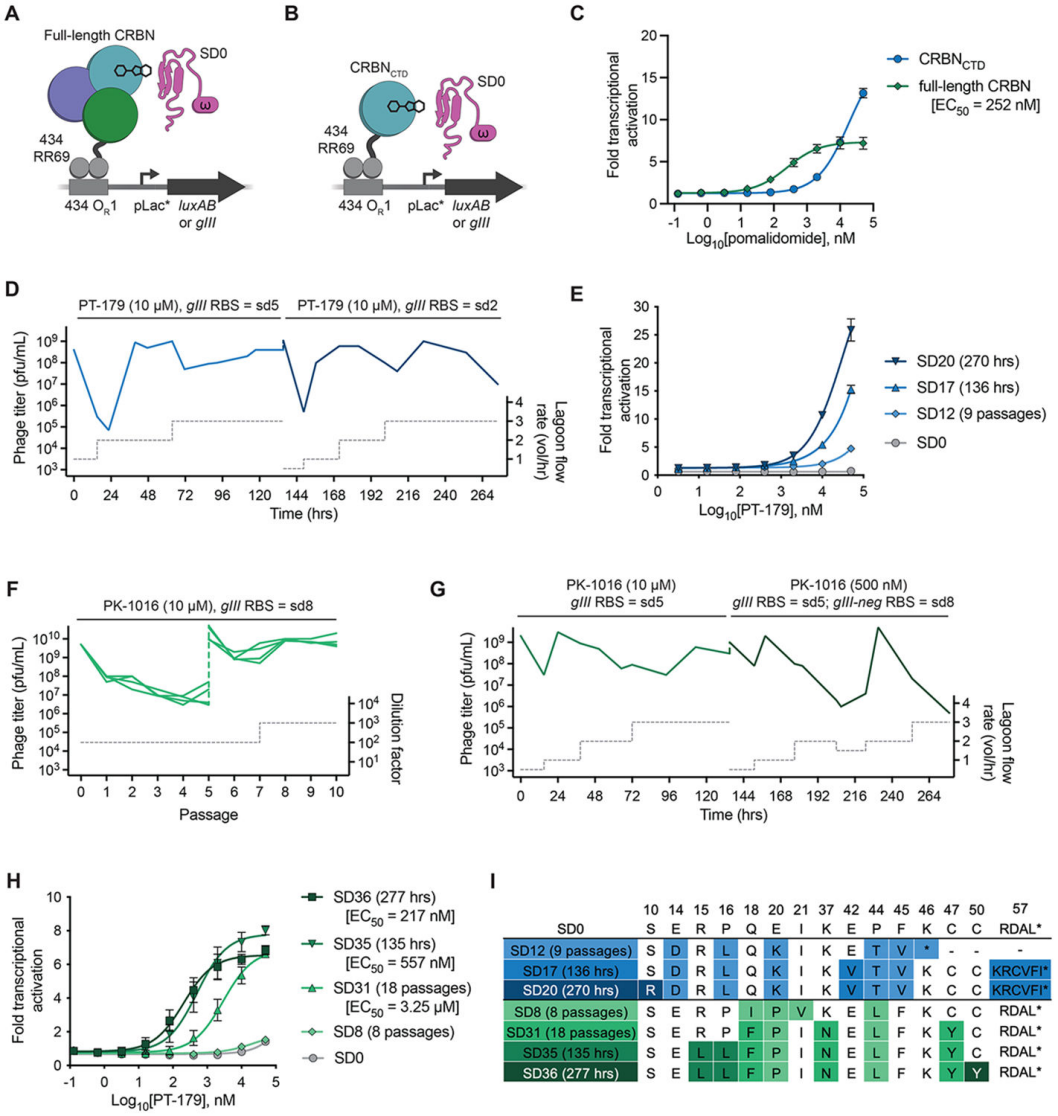
Phage-assisted continuous evolution of new ZF degrons. (**A**) Full-length CRBN MG-PACE circuit. (**B**) CRBN_CTD_ MG-PACE circuit. (**C**) Pomalidomide-induced activation of both CRBN MG-PACE circuits (1 hr). (**D**) PACE in CRBN_CTD_ circuit. (**E**) PT-179-induced CRBN_CTD_ circuit activation with evolved variants (1 hr). (**F**) PANCE in the full-length CRBN circuit. (**G**) PACE in the full-length CRBN circuit. (**H**) PT-179-induced full-length CRBN circuit activation with evolved variants (1 hr). (**I**) Mutations in PANCE- and PACE-evolved degron variants. SD17 and SD20 contain a frame-shift mutation recoding residues 57–60 and extending the reading frame by two residues. Values and error bars in (C), (E), and (H) represent the mean and standard deviation of three replicates, each normalized to a control treatment with DMSO only. Dashed vertical lines in (D), (F), and (G) represent neutral drift passages.

To evolve ZFs that engage PT-179-bound CRBN, we constructed an MG-PACE circuit using the CRBN•IMiD•ZF molecular glue complex. As a starting ZF, we chose a 60-amino acid chimera comprised of IMiD neosubstrates IKZF1 and ZFP91 that was previously developed as a potent IMiD-responsive ‘super-degron’, hereafter referred to as SD0 (14). We translationally fused CRBN to the DNA-binding protein RR69 and fused SD0 to the RNAP ω-subunit, then measured transcriptional activation by luminescence (Fig. 2A). Pomalidomide elicited a sigmoidal dose-response curve (EC_50_ = 252 nM), but with only 7-fold maximal transcriptional activation (Fig. 2C). Increasing or decreasing expression levels of RR69–CRBN or ω–SD0 did not improve transcriptional activation (fig. S3).

We also explored anchoring the circuit with the CRBN C-terminal domain (CTD) alone, which contains the IMiD binding pocket (Fig. 2B) (65, 69). The CRBN_CTD_ MG-PACE circuit exhibited 13-fold transcriptional activation at the highest dose, albeit with a rightward shift of the dose-response curve. This shift likely reflects a decrease in affinity of pomalidomide toward CRBN_CTD_ (69), a decrease in affinity of SD0 toward CRBN_CTD_•pomalidomide, or both (Fig. 2C). We attributed the difference in maximum circuit activation to poor expression of full-length CRBN in *E. coli*. In our initial efforts, we used the CRBN_CTD_ MG-PACE circuit, hypothesizing that higher activation (lower selection stringency) would better support weak-binding SD0 variants in the early stages of evolution.

Attempts to evolve SD0 to bind CRBN_CTD_•PT-179 repeatedly failed, suggesting that too many mutations were required to access SD0 variants capable of supporting phage propagation. In order to bridge this mutational gap, we revisited the CRBN•pomalidomide•IKZF1 co-crystal structure (9) and identified three residues that we predicted might be important for accommodating bumped IMiD derivatives. We cloned a library of phage encoding all possible mutations at the three corresponding positions in SD0 (Q18, E20, and I21) using NNK codons (N = A,T,C,G and K = G,T), but were still unable to obtain variants that conferred PT-179-dependent phage propagation.

We hypothesized that IMiD derivatives with smaller substituents might serve as evolutionary stepping-stones that would guide the evolution of SD0 to accommodate larger substitutions. We previously identified dozens of substituted IMiD derivatives that exhibit only partial disruption of neosubstrate degradation (66). We selected a panel of 16 IMiD derivatives encompassing a range of neosubstrate degradation propensities and conducted 96 simultaneous PANCE experiments separately using each of the 16 IMiD derivatives (fig. S4). Though slower than PACE, PANCE is easily parallelized in multi-well plates or automated systems (53, 70) and prevents washout of weakly propagating phage, providing a greater opportunity for weakly active early-stage solutions to evolve in response to challenging problems. Each lagoon was seeded with phage encoding either SD0 or the SD0 library, and PANCE passages were conducted in media supplemented with a target IMiD derivative.

After 8–9 passages reaching an average overall dilution of 10^15^–10^17^-fold, we obtained 15 pools of phage (10 originating from SD0 library phage, 5 from SD0 phage) that propagated strongly in the presence of one of five target IMiD derivatives (fig. S5). Successful evolutions occurred in lagoons supplemented with IMiD derivatives that have the highest propensity to degrade pomalidomide neosubstrates. Despite differences in the structures of IMiD derivative stepping-stones, evolved SD0 mutants in surviving phage converged on similar mutations (fig. S6). We characterized variants SD8 and SD12 and found that they elicit dose-dependent luciferase expression from the CRBN_CTD_ MG-PACE circuit in response to their respective IMiD derivatives, and to a lesser extent in response to PT-179 (fig. S7). These results indicate that evolving with alternate IMiD derivatives as evolutionary stepping-stones succeeded in producing SD0 variants that bind PT-179-bound CRBN_CTD_.

From these initial evolved variants, we pursued two evolutionary trajectories (fig S8). First, we subjected phage encoding SD12 to 270 hours of PACE (representing hundreds of generations of phage mutation, selection, and replication for each surviving phage), on the CRBN_CTD_ MG-PACE circuit in media supplemented with 10 μM PT-179 (Fig. 2D). We increased selection stringency by decreasing the strength of the ribosome binding site governing *gIII* translation (71), requiring more transcription events from the MG-PACE hybrid promoter to sustain adequate pIII production. Surviving phage showed evolutionary convergence on the SD20 variant, which evolved non-silent mutations in 12 of the original 60 residues in SD0 and the addition of two C-terminal residues (Fig. 2I, fig. S8D). SD20 induces robust luciferase expression from the CRBN_CTD_ MG-PACE circuit in response to PT-179 (24-fold activation at 50 μM PT-179) (Fig. 2E). These results suggest that SD20 evolved to bind CRBN_CTD_•PT-179 with affinity comparable to that of SD0 binding CRBN_CTD_•pomalidomide.

Second, we evolved SD8 using the full-length CRBN circuit (fig. S8C). We conducted 10 PANCE passages (10^24^-fold overall dilution) followed by 135 hours of PACE (Fig. 2F,G) using PK-1016 (Fig. 1D), a close analog of PT-179 with a fluorine at the phthalimide 6-position. PK-1016 binds CRBN with 2-fold lower affinity than PT-179 (K_D_ = 1,000 nM, fig. S2C), and we reasoned that this change could further increase selection stringency. These efforts yielded SD35, which potently activated the full-length CRBN MG-PACE circuit in response to PT-179 (EC_50_ = 557 nM, Fig. 2H,I, fig. S8C,D). We also attempted to augment selection stringency further by lowering RBS strength but observed phage washout.

We hypothesized that reducing the concentration of PK-1016 might increase stringency by reducing the fraction of bound CRBN during selection. To discourage the evolution of variants capable of PK-1016-independent CRBN binding, we performed simultaneous negative selection using a mutant CRBN (Y384A W386A, CRBN^YW/AA^) that is deficient in IMiD binding (72). To implement this negative selection, we constructed a single-chain version of the DNA-binding P22 phage repressor c2 (scP22) (73), translationally fused scP22 to CRBN^YW/AA^, and placed the p22 O_L_1 operator upstream of *gIII-neg*, a dominant-negative form of *gIII* (fig. S9) (38). SD35 variants that bind CRBN without an IMiD derivative should also bind CRBN^YW/AA^, triggering expression of pIII-neg and poisoning the production of infectious progeny phage. We evolved the SD35-containing pool of phage surviving the previous 135 hours of PACE for an additional 142 hours using this strategy and lowered the concentration of PK-1016 from 10 μM to 500 nM (Fig. 2G, fig. S8C). The resulting evolved variant surviving both positive and negative selection, SD36, contains eight mutated residues (Fig. 2I, fig. S8D) and elicits a potent sigmoidal PT-179 dose-response curve in the full-length CRBN MG-PACE circuit (EC_50_ = 217 nM, Fig. 2H).

### Identification of SD40, a compact PT-179-dependent degradation tag

**Figure 3.**
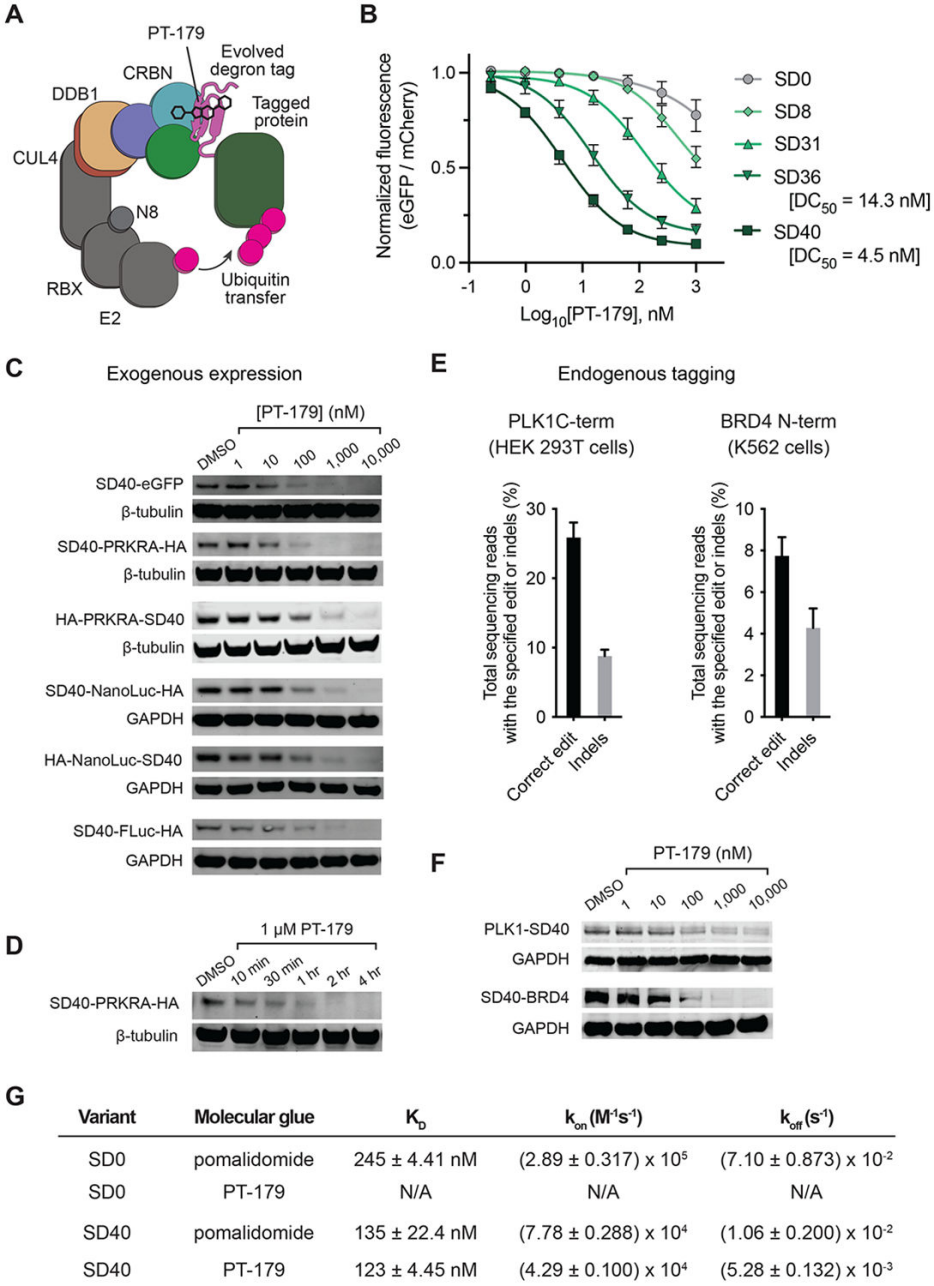
SD40 degrades exogenously- and endogenously-expressed tagged proteins in human HEK293T and K562 cells. (**A**) Cartoon representation of PT-179 recruiting a degron-tagged protein to the CUL4•RBX1•DDB1•CRBN E3 ligase complex. (**B**) PT-179-induced eGFP degradation (≥20 hrs) with evolved degrons in HEK293T cells. (**C**) Degradation of proteins fused to SD40 (24 hrs). The corresponding genes were genomically integrated in HEK293T cells using a lentiviral vector. (**D**) Time course of PT-179-induced degradation of genomically expressed SD40-PRKRA in HEK293T cells. (**E**) Insertion of a 108-bp sequence encoding SD40 in-frame in endogenous genomic target genes using twin prime editing. SD40 was precisely inserted before the endogenous *PLK1* stop codon in HEK293T cells and after the endogenous *BRD4* start codon in K562 cells. (**F**) Degradation of endogenous SD40-tagged PLK1 (2 hrs) and BRD4 (24 hrs) in cell lines generated by prime editing. (**G**) Thermodynamic and kinetic binding parameters for SD0 and SD40 binding to CRBN•pomalidomide or CRBN•PT-179. Values and error bars in (B) represent the mean and standard deviation of three replicates, normalized to control treatment with DMSO only. Values and error bars in (E) represent the mean and standard deviation of three replicates. Thermodynamic and kinetic binding parameters in (G) reflect the mean and standard error of three replicates.

Next, we characterized the ability of evolved degrons to recruit tagged proteins to the endogenous CUL4A/B•RBX1•DDB1•CRBN (CRL4^CRBN^) E3 ligase complex in human cells, triggering ubiquitination and proteasomal degradation in response to PT-179 (Fig. 3A). We generated stable HEK293T cell lines expressing degron variants fused to eGFP with untagged mCherry as an internal control for expression (74). SD20, which evolved in the CRBN_CTD_ selection circuit, did not trigger eGFP degradation in response to PT-179 despite its marked improvement in activating the CRBN_CTD_ MG-PACE circuit (fig. S10A,B). SD20 contains several mutations in the C-terminal portion of the degron (Fig. 2I, fig. S10A). This region of IKZF1 has been predicted through molecular dynamics to engage CRBN_NTD_, and mutating certain residues within this region such as R57 (which is mutated in SD20) impairs ternary complex formation *in vitro* (9). We speculate that evolution in the absence of CRBN_NTD_ led to C-terminal mutations in SD20 that impede ternary complex formation with full-length CRBN, as evidenced by SD20 only weakly activating the full-length CRBN MG-PACE circuit in response to PT-179 (fig. S10C). Consistent with this model, reverting the mutated C-terminal residues in SD20 lead to an increase in transcriptional activation with full-length CRBN and PT-179 (fig. S10C).

In contrast, variants evolved in the full-length CRBN selection circuit induced PT-179-dependent eGFP degradation in reporter HEK293T cells, with more evolved variants exhibiting greater potency (Fig. 3B). SD36 potently degraded eGFP in response to PT-179 with a half-maximal degradation (DC_50_) of 14.3 nM. These results demonstrate that the full-length CRBN MG-PACE circuit successfully evolved ZF degrons that degrade tagged proteins in human cells in response to bumped IMiD derivative PT-179.

The eight mutations in SD36 occur in the center of the degron, between residues 15 and 50 (fig. 2I, fig. S11A). We hypothesized that portions of the N- and C-termini of the degron that were not mutated during evolution might be dispensable for its engagement with CRBN•PT-179. We successively truncated the N- and C-terminal ends of SD36 and identified a 36 amino-acid ‘minimal’ degron, SD40, that exhibited 3.2-fold enhanced potency for PT-179-induced GFP degradation (DC_50_ = 4.5 nM, Fig. 3A, S11A). Trimming the degron beyond this point led to a marked reduction in degradation potency (fig. S11B). Although degron variants were evolved to bind CRBN•PK-1016, PK-1016 is a poor degrader of SD40-eGFP in HEK293T cells, suggesting that the added fluorine is disruptive for cell penetration (fig. S11C). SD40 remains potently responsive to canonical IMiD derivatives such as pomalidomide and mezigdomide (fig. S11D, E).

To test the versatility of SD40, we generated five stable HEK293T cell lines that exogenously express N- or C-terminal SD40 fusions with Nluc, Fluc, and the kinase activator PRKRA. We observed robust dose-dependent degradation of each fusion construct in response to PT-179 (Fig. 3C). A time-course experiment revealed that PRKRA degradation occurred within 10 minutes of 1 μM PT-179 administration and degradation was nearly complete within one hour (Fig. 3D). To investigate whether SD40 targets unintended neosubstrates for degradation, we performed global proteomics using HEK293T cells stably expressing SD40–eGFP. After 5 hours of treatment with pomalidomide, SD40-eGFP was significantly degraded alongside multiple known endogenous neosubstrates of pomalidomide (fig. S12A). In contrast, after 5 and 24 hours of treatment with PT-179, SD40–eGFP was the only significantly degraded protein (no other protein out of ~7,800 detected was degraded ≥ 2-fold at *p* ≤ 0.0001; fig. S12B,C). These results corroborate that SD40 mediates rapid and selective PT-179-triggered degradation of various tagged proteins as N- or C-terminal fusions.

### SD40 can be installed in target genes via prime editing

Next, we evaluated the installation of SD40 at endogenous genomic target genes in mammalian cells using twin prime editing (twin PE) (54, 75). We optimized prime editing parameters, including spacer position and prime editor construct (30), to insert SD40 at the C-terminus of PLK1 with 26% editing efficiency and 8.8% indel byproducts in HEK293T cells (Fig. 3E, fig. S13), enabling rapid isolation of a homozygous PLK1-SD40 cell line. PLK1 is an essential cell-state regulator (76), and complete knockdown for 24 hours is cytotoxic. After 2 hours of treatment with PT-179, we observed robust PLK1 knockdown (Fig. 3F). We also used twin prime editing to install SD40 at the N-terminus of BRD4 yielding 7.7% knock-in of SD40 in K562 cells (Fig. 3E, S13) with 4.3% indel byproducts. From a pool of prime-edited cells we isolated a homozygous line expressing SD40–BRD4 and observed that PT-179 effected robust degradation (Fig. 3F). For comparison, we also attempted scarless insertion of SD40 and FKBP12^F36V^ from the dTag system (324 base pairs) (17) at the N-terminus of BRD4 using Cas9 nuclease-directed HDR using two different sgRNAs and a double-strand donor DNA template. Cas9-mediated insertion of SD40 was more efficient (5.2–8.7% editing) than the larger FKBP12^F36V^ (0.61–0.82% editing), though indel products predominated in both cases (77–86% indels for SD40, 93–96% indels for FKBP12^F36V^, fig. S14). These findings indicate that SD40 is amenable to installation into native target genes in human cells by prime editing with favorable ratios of the desired edit to indel byproducts, facilitating the evaluation of conditional degradation of an endogenous target protein of interest expressed in its native genomic regulatory context.

### SD40 tightly binds CRBN•PT-179 in vitro

To compare the affinity of the evolved ternary complex CRBN•PT-179•SD40 to that of the original complex CRBN•pomalidomide•SD0, we expressed and purified representative SD40 and SD0 constructs from *E. coli* as fusions to maltose binding protein (MBP). We co-expressed and co-purified CRBN with a truncated version of its adaptor protein, DNA damage-binding protein 1 (DDB1^ΔBPB^ •CRBN) from *Trichoplusia ni* Hi Five cells. We conducted bio-layer interferometry (BLI) with immobilized MBP–degron to measure association and dissociation rates of DDB1^ΔBPB^ •CRBN precomplexed with either PT-179 or pomalidomide. As expected, SD0 binds CRBN•pomalidomide (K_D_ = 217 nM) but not CRBN•PT-179 (Fig. 3G, fig. S15A). In contrast, SD40 binds CRBN precomplexed with both molecules (CRBN•pomalidomide•SD40 K_D_ = 135 nM, CRBN•PT-179•SD40 K_D_ = 123 nM, fig. S15B). Equilibrium binding measurements using time-resolved fluorescence resonance energy transfer (TR-FRET) yielded data consistent with these findings (Fig. S15C,D). BLI revealed that SD40 binds pre-complexed CRBN with slower on- and off-rates relative to SD0 (Fig. 3G), suggesting that the MG-PACE circuit may select for longer residence times at the evolving CRBN•PT-179•degron interface. Finally, we investigated whether the presence of SD40 increases PT-179’s apparent affinity for CRBN in human cells. We observed a 1.8-fold increase in affinity in HEK293T cells stably expressing SD40–PRKRA and a further 3.8-fold increase in HEK293T cells overexpressing SD40–PRKRA (fig. S16). Overall, these data indicate that MG-PACE successfully evolved a high-affinity molecular glue interaction with PT-179-bound CRBN.

### Structural characterization of the DDB1•CRBN•PT-179•SD40 complex

**Figure 4.**
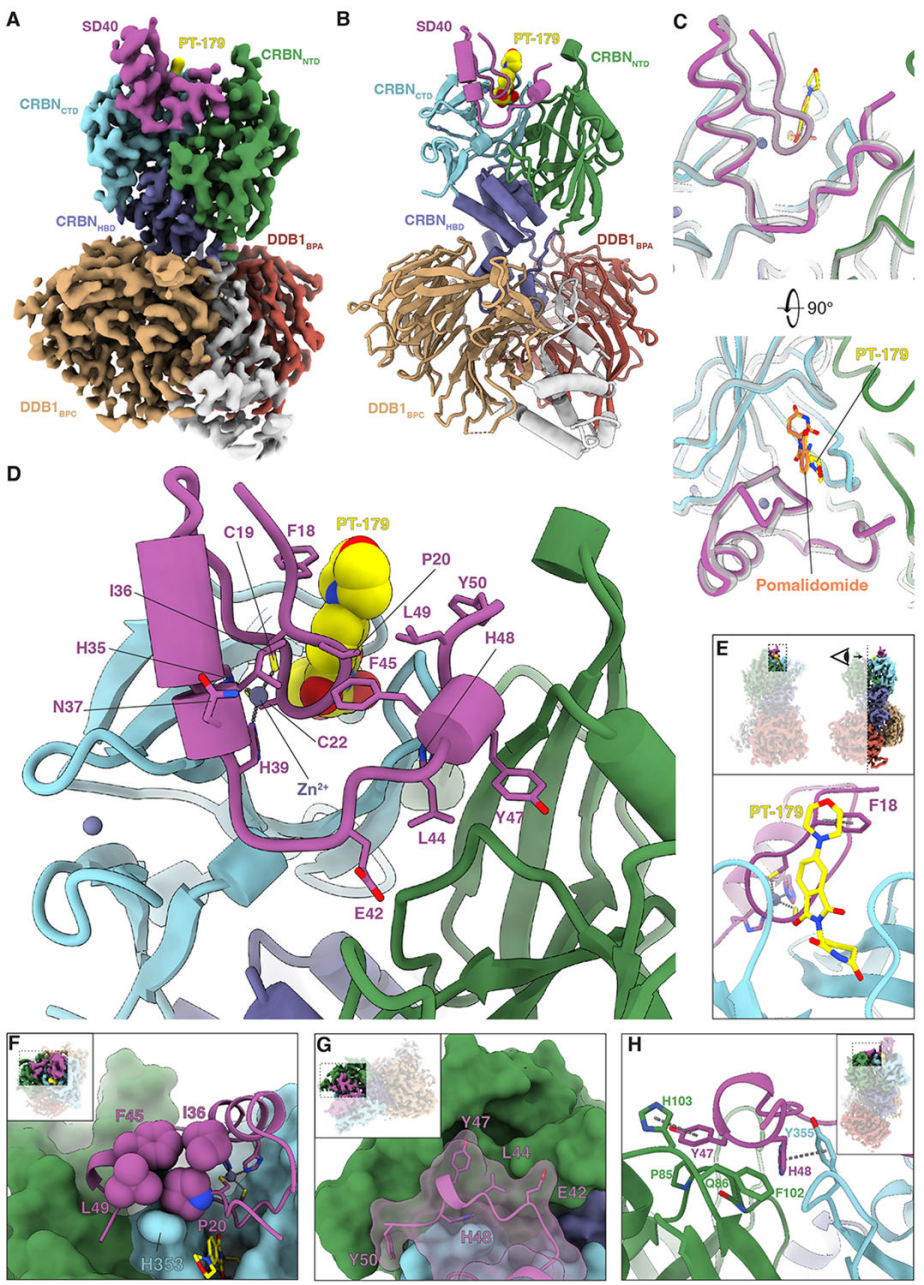
Cryo-EM structure of the ternary complex of SD40 with DDB1•CRBN bound to IMiD derivatives. (**A**) EM map of DDB1^ΔBPB^•CRBN•PT-179•SD40, colored by domains. Map obtained from local refinement on CRBN and sharpened using deepEMhancer (98). (**B**) Cartoon model of DDB1^ΔBPB^•CRBN•PT-179•SD40 with PT-179 highlighted in space-filling representation. (**C**) Comparison of the structures of SD40 bound to CRBN•PT-179 and to CRBN•pomalidomide. Backbone trace of DDB1^ΔBPB^•CRBN•pomalidomide•SD40 is shown in translucent gray superimposed on DDB1^ΔBPB^•CRBN•PT-179•SD40 in color aligned on the CRBN residues around the ligand. (**D**) Closeup of the cartoon model in (B) near the CRBN•PT-179•SD40 interface with key residues of SD40 shown in stick representation. (**E**) Interaction of F18 and PT-179 though stacking of the side chain against the morpholine ring of the compound. (**F**) A constellation of hydrophobic residues (P20, I36, F45, and L49) shown in space-filling representation. These residues pack tightly against each other and H353 of CRBN. (**G**) Additional interactions made by the C-terminal tail of SD40 with CRBN_NTD_. CRBN is shown in surface representation and SD40 is shown as a transparent surface and cartoon. SD40 residues contributing most to the interaction are shown in stick representation. (**H**) Interaction of Y47 and H48 of SD40 with CRBN residues. π–π stacking interactions with H103 in CRBN_NTD_ and Y355 in CRBN_CTD_ are highlighted. In panels E–H, figure insets show the map orientation, depth cue, and cropping applied to arrive at that panel.

Evolved mutations in SD40 cluster near the C-terminal region and IMiD contact site. To understand how they influence the affinity of SD40 to CRBN, we determined cryo-EM structures of SD40 in complex with DDB1^ΔBPB^•CRBN bound to PT-179 or to pomalidomide. With PT-179, three-dimensional classification revealed heterogeneity at the DDB1^ΔBPB^•CRBN interface implying relative motion between the two proteins (Movie S1). Hence, after multiple rounds of refinement, we obtained two distinct high-quality maps for DDB1^ΔBPB^•CRBN•PT-179•SD40 at global resolutions of ~2.4 and ~2.5 Å, respectively (Fig. S18B), which were then used to build models (Fig. 4A,B, fig. S18, S19). For comparison with a conventional IMiD, we also built a model of DDB1^ΔBPB^•CRBN•pomalidomide•SD40 using a map refined to a global resolution of ~3.3 Å (Fig. 4C, fig. S17, S19H). Additional compound density in the PT-179 maps compared to the pomalidomide map allowed unambiguous placement of the morpholine ring of PT-179 (fig. S19I,J).

The cryo-EM structures with PT-179 reveal an extensive interface between SD40 and CRBN (Fig. 4D) bridging the IMiD-binding C-terminal and N-terminal domains of CRBN. In IKZF1, Q146 (equivalent to Q18 in SD0) forms a water-mediated hydrogen bond with the phthalimide amino group of pomalidomide (9). As SD40 evolved to bind PT-179 and PK-1016, both of which lack this amino group, this hydrogen bonding opportunity was lost. Instead, the side chain of F18 packs against the electron-rich morpholine substituent at the C5 position of the phthalimide ring of PT-179 (Fig. 4E, fig. S20). The C-terminal tail (residues 42–50) of SD40 folds back on the zinc finger motif and is stabilized by a constellation of hydrophobic residues—P20, I36, F45, and L49 (Fig. 4F). E20P (arising in SD8) acts as a gatekeeper mutation that enables this fold by tightly packing against the three other residues as well as H353 of CRBN.

The additional contacts of the SD40 C-terminal tail with CRBN at residues E42, L44, Y47, H48, and Y50 result in an increased buried solvent-accessible surface area of 2,293 Å^2^ at the CRBN•SD40 interface compared to 1,098 Å^2^ without the tail (Fig. 4G). Moreover, this interface has a high shape complementarity score (77) of 0.68 (on a scale of 0 to 1, with 1 being perfectly complementary). The mutation P44L arose in SD8 without the presence of CRBN_NTD_. Upon introduction of full-length CRBN, it likely bound a hydrophobic patch of CRBN around M88 and anchored the C-terminal tail on CRBN supporting subsequent mutations (map quality insufficient to unambiguously assign rotamers near CRBN_M88_). Y47 and H48 are well-positioned to make favorable interactions with several CRBN residues, notably π–π stacking interactions with H103 and Y355, respectively, bridging the N- and C-terminal domains of CRBN (Fig. 4H). C47Y arose in SD31, which coincided with a sharply increased circuit activation compared to SD8 (Fig. 2H). Conversely, trimming of H48–Y50 in SD41 led to a marked reduction in degradation signal compared to SD40 (fig. S11A,B).

Side-chain densities for Y50 of SD40 and F150 of CRBN were not resolved in the map, but the backbones are well-positioned to form π–π stacking interactions suggesting that the final mutation C50Y may increase affinity through this potentially transient interaction. Mezigdomide was recently shown to promote the closed (putatively active) conformation of CRBN by recruiting the N-terminal domain via interactions near F102 and F150 (11), both of which are at the CRBN•SD40 interface. These results suggest that SD40 and mezigdomide take advantage of a shared strategy for CRBN activation by making non-overlapping contacts across both CRBN_CTD_ and CRBN_NTD_, thus explaining the highly potent degradation activity of mezigdomide (fig. S11E).

The R15L, P16L, and K37N mutations are not present at the CRBN•PT-179•SD40 interface and likely evolved to influence MG-PACE circuit activation by allosteric or other mechanisms. The lower helical propensity of asparagine compared to lysine (78) may have resulted in premature termination of the helix at position 38—which, in prior structures (9) extends to residue 40. This early helix termination turns the SD40 C-terminal tail towards CRBN_NTD_ and provides the opportunity to make the aforementioned contacts (fig. S20A).

The structure of the CRBN•pomalidomide•SD40 interface is similar to that of CRBN•PT-179•SD40, with the SD40 C-terminal tail adopting the same looped conformation (Fig. 4C), indicating that the binding mode of SD40 is not specific to PT-179. Given this molecule-agnostic mode of binding, we hypothesized that the reversion mutation F18Q in SD40 would restore the water-mediated hydrogen bonding interaction seen with pomalidomide (9). Indeed, reverting the F18 mutation in SD40 led to a 5.6-fold improvement in potency with pomalidomide (DC_50_ = 177 pm, fig. S20C). This SD40 revertant displayed a modest 2.2-fold reduction in potency with PT-179 (DC_50_ = 10.1 nM, fig. S20D). Modeling SD40 and the revertant using Rosetta (79) predicted that glutamine avoids a steric clash with the morpholine substitution on PT-179, but its interaction with PT-179 is less energetically favorable than that of phenylalanine (fig. S20B, table S7). These observations highlight that the interaction between the SD40 C-terminal tail and CRBN_NTD_ is the primary driver of affinity.

Collectively, the high-resolution structures of the DDB1^ΔBPB^•CRBN•PT-179•SD40 ternary complex reveal that the mutations in SD40 evolved to take advantage of numerous types of interactions between the evolved degron, CRBN, and PT-179. These include new polar and hydrophobic interactions with mutated residues, conformational rearrangement in non-mutated residues so they can participate in new interactions with CRBN, new interactions within SD40 including a well-packed hydrophobic cluster, and positioning complementary SD40 groups next to nearly every reachable surface of partner molecules (Fig. 4D–H). Hence, these models illustrate that a genetically encodable evolved degron can exploit interactions with regions of CRBN previously known to engage current-generation small-molecule drug candidates such as mezigdomide as well as entirely novel regions.

### Evolution of a mouse CRBN-compatible degron tag

**Figure 5.**
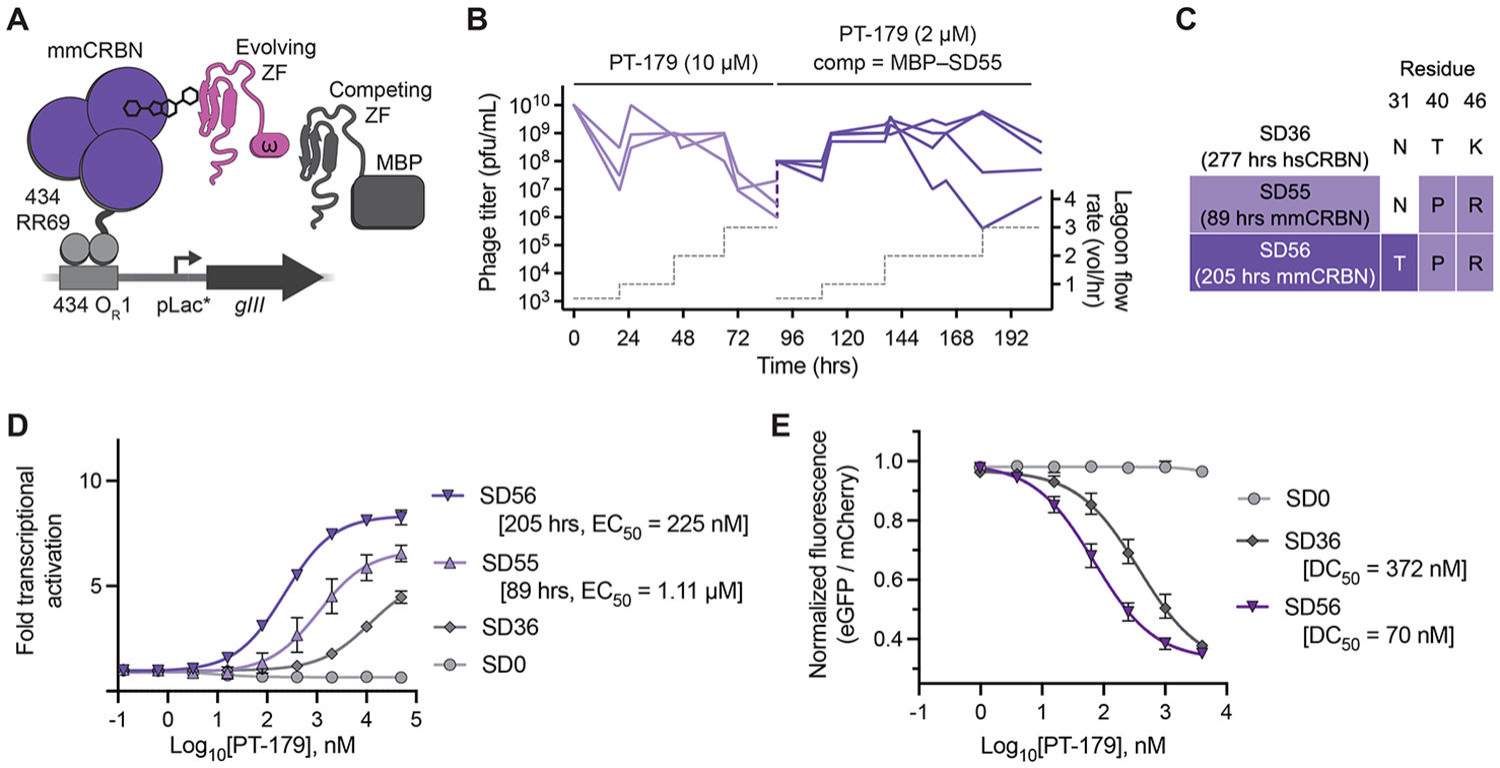
MG-PACE evolves degrons that engage PT-179-bound mouse CRBN. (**A**) The mmCRBN MG-PACE circuit. Stringency is increased by expression of a competing ZF degron fused to maltose binding protein (MBP). (**B**) PACE in the mmCRBN MG-PACE circuit. (**C**) Mutations in evolved PT-179•mmCRBN-binding degrons. (**D**) PT-179-induced mmCRBN circuit activation (1 hr) by SD0 and evolved variants. (**E**) PT-179-induced degradation (≥20 hrs) of degron-tagged eGFP in mouse 3T3 cells. Values and error bars in (D) and (E) represent the mean and standard deviation of three replicates, each normalized to control treatment with DMSO only, except for the highest PT-179 concentration in (E), which used two replicates.

Compared to human CRBN, mouse cereblon (mmCRBN) contains a V388I mutation that prevents IMiD-induced recruitment of ZF neosubstrates (8). The development of a potent and compact degron that is compatible with mmCRBN would facilitate studies that modulate target protein function in mouse systems. We hypothesized that MG-PACE could evolve degrons that overcome this difference in mmCRBN and respond to PT-179 in mouse cells. We constructed a mmCRBN MG-PACE circuit (Fig. 5A) and performed a total of 205 hours of PACE seeded with SD36-encoding phage (Fig. 5B). SD0 did not activate the mmCRBN circuit, but SD36 showed activity for mmCRBN•PT-179 (Fig. 5D). The additional contacts made by the C-terminal tail of SD36 to the CRBN N-terminal domain may allow it to tolerate a marginal change in binding mode of the ZF due the steric occlusion caused by V388I.

After 89 hours of PACE, we identified the SD55 variant with improved activation of the mmCRBN MG-PACE circuit (EC_50_ = 1.11 μM, Fig. 5C,D). We hypothesized that expression of a competing degron variant in PACE would impose selection pressure on the evolving degron to increase binding affinity to mmCRBN•PT-179 (Fig. 5A, fig. S8A,B). We conducted an additional 116 hours in host cells expressing a fusion of MBP to SD55 and identified variant SD56 with an additional 4.9-fold improvement in the EC_50_ of circuit activation (EC_50_ = 225 nM, Fig. 5D).

To test whether MG-PACE produced a PT-179 responsive degron tag that is functional in mouse cells, we transduced mouse 3T3 cells with degron–eGFP–IRES–mCherry constructs for SD0, SD36, and SD56. Following overnight treatment with PT-179, we observed no signs of eGFP degradation when fused to SD0, but SD36 and SD56 elicited dose-dependent eGFP degradation (Fig. 5E). SD56 exhibited greater potency than SD36 in mouse cells (SD56 DC_50_ = 70 nM vs. SD36 DC_50_ = 372 nM, Fig. 5E). SD56 also responds to canonical IMiD derivatives in 3T3 cells, such as pomalidomide and mezigdomide (fig. S21A–C), and to pomalidomide and PT-179 in human HEK293T cells with potencies similar to SD40 (fig. S21D,E). Canonical IMiDs do not have neosubstrates in mouse cells (80). To verify that PT-179 does not recruit neosubstrates, we performed global proteomics analysis in mouse Ba/F3 cells treated with PT-179 for 5 and 24 hours and detected no significant changes to protein expression (no downregulation ≥ 2-fold at *p* ≤ 0.0001; fig. S22). Collectively, these data demonstrate that MG-PACE can be applied using mouse E3 ligase domains to evolve potent degrons that function in mouse cells.

## Discussion

Small molecule-induced degrons have become a major focus of both basic research and clinical applications. Among the handful of well-characterized degrons, none are both small enough for efficient genomic knock-in and compatible with a bio-orthogonal ligand that avoids degradation of endogenous neosubstrates, including important transcription factors such as IKZF1/3, ZFP91, and SALL4. We developed a molecular-glue PACE system that enables rapid and continuous evolution of high-affinity interactions between a protein of interest and a small-molecule-bound target. Applying the MG-PACE system evolved potent and selective molecular glue interactions between new ZF degrons and CRBN bound to orthogonal IMiD derivatives. The resulting chemically inducible degron is only 36 amino acids, can be efficiently inserted at genomic loci using prime editing, and responds to a small-molecule IMiD derivative that perturbs far fewer endogenous neosubstrates than canonical IMiD derivatives. We also applied MG-PACE to mmCRBN evolution, overcoming unfavorable residue-neosubstrate contacts, allowing the evolution of a degron that responds to PT-179 in mouse cells. Together, these findings provide powerful tools that enable manipulation of protein levels in human and mouse cells with exquisite specificity. These tools should be especially useful to study protein function or validate potential therapeutic targets while preserving endogenous regulatory mechanisms that govern protein levels, thereby maximizing the biological relevance of the findings.

Collectively, our results establish MG-PACE as a powerful tool for remodeling molecular glue interactions. We developed gene circuits for simultaneous positive and negative selection and tools to modulate selection stringency by controlling *gIII* RBS strength and expressing competing binders. Phage encoding SD36 experienced an overall 10^271^-fold dilution over 18 serial PANCE passages and 277 hours of PACE, corresponding to the shortest linear path experiencing hundreds of generations of degron mutation, selection, and replication. MG-PACE may also be useful to provide compact degron tags that are compatible with the large diversity of other IMiD derivatives that have been developed (81), allowing IMiDs with especially desirable pharmacologic properties to be applied to targeted protein degradation or other molecular glue applications.

Our finding that active degrons evolved exclusively in the presence of full-length CRBN supports the emerging picture that the closed form of CRBN, in which the N-terminal domain in close proximity to the IMiD-binding C-terminal domain, is the active conformation for ubiquitin transfer (9, 11). The ~2.4 Å global resolution cryo-EM structure of the ternary complex establishes that degrons can evolve substantial interactions with both CRBN_NTD_ and CRBN_CTD_ to drive CRBN towards the closed state. This study also identified previously unrecognized regions in CRBN_NTD_ that can be targeted to improve the affinity of CRBN-modulating drugs and additional degrons. This enhanced understanding of CRBN complexed with a potent, laboratory-evolved degron thus may inform the development of next-generation CRBN-based therapeutics.

## Materials and Methods

### General methods

Antibiotics were purchased from Gold Bio and used at the following concentrations: streptomycin (50 μg/mL), chloramphenicol (25 μg/mL), carbenicillin (50 μg/mL), spectinomycin (50 μg/mL), tetracycline (10 μg/mL), and kanamycin (50 μg/mL). Nucleic acid samples were resuspended in either buffer EB (Qiagen, 19086) or nuclease-free water (Qiagen, 129117). PCR reactions for cloning were performed with Phusion U Multiplex PCR Master Mix (Thermo Fischer, F562L). Gene blocks and primers were ordered from Integrated DNA Technologies. PCR reactions for sequencing phage variants were performed with GoTaq Green Master Mix (Promega, M712B) or Phusion U Multiplex PCR Master Mix (Thermo Fischer, F562L). Bacterial plasmids were cloned into either Mach1 (Thermo Fisher Scientific, C862003) or Turbo (NEB, C2984) chemically competent cells. Lentiviral vectors were cloned into Stbl3 chemically competent cells (Thermo Fisher Scientific, C737303). Polymyxin B nonapeptide (PMBN) was purchased from Chemieliva. Pomalidomide was purchased from Combi-Blocks. All PACE experiments were performed in the S2060 strain (34).

IMiD derivatives AD-45, AD-46, AD-55, AD-64, PK-802, PK-803, PK-804, PK-806, PK-1016, PT-176, PT-178, PT-179, PT-187, PT-198, VS-775, VS-777, and VS-786 were synthesized as previously described (66).

### Luminescence assays

Chemically competent S2060 cells were co-transformed with accessory and complementary plasmids (Table S1). Cells were plated on LB agar supplemented with carbenicillin, spectinomycin, and tetracycline and grown overnight at 37 °C. Single colonies were seeded in 1 mL DRM with carbenicillin and spectinomycin and grown overnight in a 37 °C shaker at 220 rpm. Overnight cultures were back-diluted 1:100 in DRM with carbenicillin and spectinomycin and grown at 37 °C with shaking until OD600 values reached 0.3-0.4. Polymyxin B nonapeptide (PMBN) was added to a final concentration of 3 μg/mL culture from a 1000x stock (3 mg/mL in DMSO). 1 μL DMSO or indicated small molecule dissolved in 1 μL DMSO was added to the bottom of a well in a 96-well black wall, clear bottom plate (Costar) and resuspended in 200 μL PMBN-treated cell culture per well. 96-well plates were then incubated in an Infinite M1000 Pro microplate reader (Tecan) at 37 °C with continuous shaking for ~1 hour prior to reading A_600 nm_ and luminescence values. Raw luminescence values were then divided by the corresponding A_600 nm_ values to normalize to cell density. EC_50_ values were calculated in GraphPad Prism (v9.5.1), using an agonist vs. response variable slope (four parameter) model.

### Cell culture conditions

HEK 293T (ATCC CRL-3216), U2OS (ATCC, HTB-96), and NIH 3T3 (ATCC CRL-1658) cells were cultured in Dubelcco’s Modified Eagle Medium (DMEM) plus GlutaMAX (ThermoFisher Scientific) supplemented with 10% (v/v) fetal bovine serum (FBS, ThermoFisher Scientific). K562, MM.1S (CRL-2974), KELLY (92110411), and MOLT-4 (CRL-1582) cells were cultured in Roswell Park Memorial Institute (RPMI) 1640 media (ThermoFisher Scientific) supplemented with 10% (v/v) FBS. All cells were maintained below 90% confluency and cultured at 37°C with 5% CO_2_. Cell lines were authenticated by their suppliers and tested negative for mycoplasma. Ba/F3 (gift from Dr. Nathanael Gray, Stanford) were cultured similar to MOLT-4 cells with the media additionally supplemented with 1 ng/mL recombinant mouse IL-3 (PropecBio CYT-371).

### Transcriptome-wide RNA sequencing

The effect of small molecule treatment on transcriptome-wide RNA expression was evaluated using bulk RNA sequencing of 5 biological replicates per sample. Briefly, 200,000 MM.1S cells were plated per well in a 24-well plate with 1 μM of either pomalidomide, PT-179, or a DMSO control with a final concentration of 0.5% v/v DMSO. 48 hours following treatment, cells were washed with PBS and lysed in RLT buffer (Qiagen) with β-mercaptoethanol. Total RNA from each sample was purified from 2–4×10^5^ cells using the RNeasy Mini Kit (Qiagen) with on-column DnaseI digestion and eluted in nuclease-free water. Polyadenylated RNA was sequenced using Smart-seq2 as previously described (82) with two changes: 20 ng of total RNA and Maxima Rnase H-minus reverse transcriptase (Thermo Fisher) were used for the initial reverse transcription step. The resulting cDNA libraries were sequenced on an Illumina NextSeq 550 High Output kit with the following run parameters: 37 cycles for read1, 37 cycles for read2, 8 cycles for index1, and 8 cycles for index2.

Sequencing data were demultiplexed with bcl2fastq and roughly 40 million paired-end reads were obtained per sample. Transcript-level quantification was performed using kallisto (83) with the *homo sapiens* GRCh38 reference transcriptome and with bootstrapping. Differential expression analysis at the gene level was conducted using Deseq2 with batch correction of biological replicates (84). To pre-filter low count genes, only genes with at least 10 counts in at least 5 samples were analyzed. Compared between conditions, genes with a transcript abundance fold-change > 2 and with a false discovery rate-corrected *P*-value < 0.01 were called as differentially expressed.

### Global quantitative proteomics sample preparation

MOLT4, Kelly, HEK293T or Ba/F3 cells were treated with DMSO, PT-179 or pomalidomide at 1 μM for 5 or 24 hr and cells were harvested by centrifugation at 4 °C before snap freezing in liquid nitrogen. Cells were lysed by addition of lysis buffer (8 M Urea, 50 mM NaCl, 50 mM 4-(2-hydroxyethyl)-1-piperazineethanesulfonic acid (EPPS) pH 8.5, Protease and Phosphatase inhibitors) and homogenization by bead beating (BioSpec) for three repeats of 30 seconds at 2400. Bradford assay was used to determine the final protein concentration in the clarified cell lysate. 50 μg of protein for each sample was reduced, alkylated and precipitated using methanol/chloroform as previously described (32) and the resulting washed precipitated protein was allowed to air dry. Precipitated protein was resuspended in 4 M Urea, 50 mM HEPES pH 7.4, followed by dilution to <1 M urea with the addition of 200 mM EPPS, pH 8. Proteins were digested with LysC (1:50; enzyme:protein) and trypsin (1:50; enzyme:protein) overnight at 37 °C. Sample digests were acidified with formic acid to a pH of 2-3 prior to desalting using C18 solid phase extraction plates (SOLA, Thermo Fisher Scientific). Desalted peptides were dried in a vacuum centrifuge and reconstituted in 0.1% formic acid for LC-MS analysis.

### diaPASEF quantitative LCMS proteomics and data analysis

Data were collected using a TimsTOF Pro2 (Bruker Daltonics, Bremen, Germany) coupled to a nanoElute LC pump (Bruker Daltonics, Bremen, Germany) via a CaptiveSpray nano-electrospray source. Peptides were separated on a reversed-phase C18 column (25 cm x 75 μm ID, 1.6 μM, IonOpticks, Australia) containing an integrated captive spray emitter. Peptides were separated using a 50 min gradient of 2 – 30% buffer B (acetonitrile in 0.1% formic acid) with a flow rate of 250 nL/min and column temperature maintained at 50 °C.

DDA was performed in Parallel Accumulation-Serial Fragmentation (PASEF) mode to determine effective ion mobility windows for downstream diaPASEF data collection (Meier et al., 2020). The ddaPASEF parameters included: 100% duty cycle using accumulation and ramp times of 50 ms each, 1 TIMS-MS scan and 10 PASEF ramps per acquisition cycle. The TIMS-MS survey scan was acquired between 100–1700 m/z and 1/k0 of 0.7–1.3 V.s/cm^2^. Precursors with 1–5 charges were selected and those that reached an intensity threshold of 20,000 arbitrary units were actively excluded for 0.4 min. The quadrupole isolation width was set to 2 m/z for m/z <700 and 3 m/z for m/z >800, with the m/z between 700–800 m/z being interpolated linearly. The TIMS elution voltages were calibrated linearly with three points (Agilent ESI-L Tuning Mix Ions; 622, 922, 1,222 m/z) to determine the reduced ion mobility coefficients (1/K_0_). To perform diaPASEF, the precursor distribution in the DDA m/z-ion mobility plane was used to design an acquisition scheme for DIA data collection which included two windows in each 50 ms diaPASEF scan. Data was acquired using sixteen of these 25 Da precursor double window scans (creating 32 windows) which covered the diagonal scan line for doubly and triply charged precursors, with singly charged precursors able to be excluded by their position in the m/z-ion mobility plane. These precursor isolation windows were defined between 400–1200 m/z and 1/k0 of 0.7–1.3 V.s/cm^2^.

The diaPASEF raw file processing and controlling peptide and protein level false discovery rates, assembling proteins from peptides, and protein quantification from peptides was performed using either targeted, cell line specific spectral libraries generated by searching offline fractionated DDApasef data against a Swissprot human or mouse database (January 2021) or using library free analysis in DIA-NN 1.8 (85). Library free mode performs an in-silico digestion of a given protein sequence database alongside deep learning-based predictions to extract the DIA precursor data into a collection of MS2 spectra. The search results are then used to generate a spectral library which is then employed for the targeted analysis of the DIA data searched against a Swissprot human or mouse database (January 2021). Database search criteria largely followed the default settings for directDIA including tryptic with two missed cleavages, carbamidomethylation of cysteine, oxidation of methionine, N-terminal acetylation and precursor Q-value (FDR) cut-off of 0.01. Precursor quantification strategy was set to Robust LC (high accuracy) with RT-dependent cross run normalization. Proteins with low sum of abundance (<2,000 x no. of treatments) were excluded from further analysis and proteins with missing values were imputed by random selection from a Gaussian distribution either with a mean of the non-missing values for that treatment group or with a mean equal to the median of the background (in cases when all values for a treatment group are missing). Protein abundances were scaled using in-house scripts in the R framework (R Development Core Team, 2014) and resulting data was filtered to only include proteins that had a minimum of 3 counts in at least 4 replicates of each independent comparison of treatment sample to the DMSO control. Significant changes comparing the relative protein abundance of these treatment to DMSO control comparisons were assessed by moderated t test as implemented in the limma package within the R framework (86).

### Fluorescence polarization

Fluorescence polarization experiments were performed according to previously reported methods (65). All experiments were performed in 25 mM HEPES, 200 nM NaCl, 1 mM TCEP, pH 7.4. Dilutions of DDB1-CRBN sample were mixed with 20 nM Cy5-thalidomide compound at a final DMSO concentration of 0.1% v/v. Solutions were incubated for 30 minutes at RT with gentle shaking (220 rpm). Polarization values (mP) were measured on a Tecan Infinite M1000 Pro microplate reader. GraphPad Prism (v9.5.1) was used to calculate the K_D_ value of cereblon and thalidomide-Cy5 (fig. S2A) using a saturated, one site binding model.

### Competitive fluorescence polarization

Competitive fluorescence polarization experiments were performed according to previously reported methods (65). All experiments were performed in 25 mM HEPES, 200 nM NaCl, 1 mM TCEP, pH 7.4. A pre-complexed sample was prepared by incubating DDB1-CRBN (400 nM) with thalidomide–Cy5 conjugate (fig. S2A) (80 nM) at RT for 30 minutes with gentle shaking (220 rpm). To initiate competition, dilutions of pomalidomide, PT-179, or PK-1016 were added to the pre-complexed CRBN sample at a final DMSO concentration of 1% v/v. Polarization values were measured on an Infinite M1000 Pro microplate reader (Tecan) following 1 hour of incubation at RT with gentle shaking. A total of three independent measurements were acquired and averaged per biological sample to generate the values shown in figure S2C. All mP values were normalized between 0 and 100 using the formula $Normalized\;\;mP=\left[\frac{\left(x-minimum\right)}{maximum-minimum}\right]*100$, where ‘‘x’’ is the mP value of the sample and ‘‘minimum’’ and ‘‘maximum’’ are the minimum and maximum mP values, respectively, across all samples. K_D_ values were estimated from computed K_i_-values as previously described (65).

### In-cell CRBN engagement NanoBRET

In-cell CRBN engagement assays were conducted with a commercial kit (Promega N2910) according to a published procedure (68). U2OS or HEK293T cells were transfected with a combination of NanoLuc-CRBN fusion vector (1 μg/ 100,000 cells) and DDB1 expression vector (4 μg/ 100,000 cells) encoded plasmid (Promega N2910) using Lipofectamine 3000 reagent (Thermo Fischer Scientific, L3000015). After 24 hours of transfection, DMSO solutions of pomalidomide and PT-179 were prepared to 10x target concentrations in a 384-well white microplate (Corning, 3765) from 10 mM of stock solutions using a Tecan D300e Digital Dispenser. The transfected cells were washed with PBS, trypsinized, and plated in a 384-well white microplate (Corning, 3765) at 3000 cells in 34 μL Opti-MEM I reduced serum medium per well. Complete 20X NanoBRET^™^ Tracer Reagent (2 μL) and 10x pomalidomide or PT-179 solutions (4 μL) were dispensed in each well. The plates were incubated for 1 hour at 37 °C, 5% CO2. After incubation plates were brought to room temperature and incubated with 3X complete substrate plus inhibitor solution (20 μL) for 3 minutes at room temperature before measuring BRET signal on an EnVision multilabel plate reader with EnVision Manager 1.13 (PerkinElmer). At each liquid handling step, the plates were shaken on a Thermo Fisher combi instrument followed by centrifugation at 100 xg for 1 minute.

Data presented in figure S16 was obtained using the above protocol with slight modifications. Lipofectamine 2000 was used in place of lipofectamine 3000 for all transfections, 1 μg of pSD192A was included for generating the SD40-PRKRA overexpression sample, and the assay was performed using 2,500 cells per well.

NanoBRET values for the in-cell CRBN engagement assay were calculated as follows:

1) Raw NanoBRET Ratio (mBU) = (618nm_Em_/460nm_Em_)×1000

2) mean corrected NanoBRET ratio mBU = Mean mBU experimental − Mean mBU no-ligand control.

EC_50_ values were calculated in GraphPad Prism (v9.5.1), using an inhibitor vs. response variable slope (four parameter) model.

### Phage-assisted non-continuous evolution (PANCE)

PANCE was conducted as previously described (34). Briefly, chemically competent S2060 *E. coli* were transformed with the desired APs and MP6, plated on 2xYT agar plates supplemented with 100 mM glucose and the necessary antibiotics. Single colonies were picked in DRM containing maintenance antibiotics, serially diluted 1:10 in fresh DRM, and grown overnight at 37 °C with shaking. The next day, culture wells that were in log-phase (OD600 ~0.3-0.6) were pooled and treated with 10 mM arabinose to induce mutagenesis. Induced host cells were then split into several 1 mL cultures in a 96-well plate and target IMiD derivatives were added to 10 μM from a 10 mM stock solution (final 0.1% DMSO v/v). Cultures were infected with phage at the reported dilutions. Infected cultures were grown for 8-16h at 37 °C with shaking for at 220 rpm. Host cells were pelleted by centrifugation at 3700 x g for 10 min and supernatant containing phage was harvested for storage and/or infection. Phage titers were determined by qPCR or plaque assay (34). For genotype determination, single plaques were picked and amplified using GoTaq Master Mix (Promega, M7122) with primers AB1396 and AB1792 (Table S4) and resultant PCR products were submitted for Sanger sequencing.

### Phage-assisted continuous evolution (PACE)

PACE was conducted as previously described (34). Briefly, chemically competent S2060 *E. coli* were transformed with the desired APs and MP6, plated on 2xYT agar plates supplemented with 100 mM glucose and the necessary antibiotics. Single colonies were picked in DRM containing maintenance antibiotics, serially diluted 1:10 in fresh DRM, and grown overnight at 37 °C with shaking. The next day, culture wells that were in log-phase (OD600 ~0.3-0.6) were used to seed a chemostat containing 60 mL of DRM. Chemostat cultures were grown to OD600 0.3–0.6 and then continuously diluted with fresh DRM at a rate that maintains cell density at OD600 0.8–1.0. Chemostat volumes were maintained at 40-60 mLs. Lagoons were 3–15 mL and supplied with arabinose (10 mM), polymyxin B nonapeptide (3 μg/mL), and target IMiD derivative (10 μM –500 nM) from a 20x stock solution in 1:1 DMSO/water using a syringe pump.

Prior to infection of every PACE experiment, phage were propagated in strain S2208 containing MP6 for 4-6 hrs with shaking in DRM supplemented with 10 mM arabinose and necessary antibiotics. Phage samples were then isolated via centrifugation and 150 μL was used to infect lagoons. Aliquots (500 μL) were taken from lagoons and centrifuged at 6000 x g for 5 minutes to isolate phage samples at indicated timepoints. Titers were determined by plaque assays using S2208 cells (34). For genotype determination, single plaques were picked and amplified using GoTaq Master Mix (Promega, M7122) with primers AB1396 and AB1792 (Table S4) and resultant PCR products were submitted for Sanger sequencing. At each timepoint, typically four plaques were picked for each lagoon.

### Lentiviral plasmid construction, virus generation, and transduction

Zinc finger (ZF) constructs were codon optimized using IDT’s codon optimization tool and ordered as gBlock gene fragments with flanking BsmBI restriction sites (Integrated DNA Technologies). Both Cilantro 2 vector (Addgene #74450) and PCR amplified and purified ZF gBlocks were digested with BsmBI-v2 (NEB R0739) following NEB’s protocol. Following digestion, Cilantro 2 vector was Quick-CIP treated (NEB M0525). Digested vector and inserts were purified using QIAquick PCR purification kit (Qiagen 128104) prior to ligation using T4 DNA ligase (NEB M0202). Ligase reaction mix was directly carried forward for transformation in One Shot Stbl3 Chemically Competent bacteria (Thermo Fischer Scientific C737303). Plasmid DNA was isolated from bacterial cells using the ZymoPURE II Plasmid Midiprep Kit (Zymo Research D4200) or the QIAGEN Plasmid Midi Kit (Qiagen 12143) according to the manufacturer’s protocols.

For lentiviral production, HEK293T cells were seeded in a 6-well, 9.6 cm^2^ dish (Corning) at a density of 7.0 x 10^5^ cells/well. The next day, cells were transfected with 1333 ng of transfer vector encoding the degradation reporter construct, 1000 ng of psPAX2 (Addgene #12260), and 667 ng of pMD2.G (Addgene #12259) using lipofectamine 2000 (Thermo Fisher Scientific 11668019). After 5-6 hours following initiation of transfection, fresh culture media was exchanged. After 48 hours, the supernatant was passed through a 0.45 μM filter (Corning) and the filtrate was stored at −80 °C for future use in lentiviral infections.

HEK293T and NIH 3T3 cells were plated in a 6-well, 9.6 cm^2^ dish (Corning) in DMEM + 10% FBS. Lentivirus was applied dropwise in the presence of 10 μg/mL polybrene (Sigma-Aldrich). Low MOI (MOI ≤ 0.3) infections were performed for all cell lines to ensure a low number of integration events. The following day, the media was exchanged for fresh DMEM + 10% FBS containing 2 μg/mL puromycin to initiate selection. Selections were carried out for ≥3 days prior to running experiments.

### Flow cytometry analysis

Flow cytometry to quantify degron-tagged eGFP degradation was performed according to previously described methods (9, 74). Briefly, following ≥3 days of antibiotic selection of lentivirally transduced HEK293T or NIH 3T3 cells, cells were plated in a poly-D-lysine 48-well, 0.75 cm^2^ plate at a density of 4.0 x 10^4^ or 8.0 x 10^3^ cells/well, respectively. The next day, cells were treated with compounds diluted to a final concentration of 0.5% DMSO/well. Following overnight treatment (≥20 hrs), cells were harvested and analyzed on Beckman CytoFLEX S or Beckman CytoFLEX LX flow cytometers to quantify eGFP and mCherry fluorescence. FLOWJO was used to analyze all flow data. Parameters were derived in the software to enable calculation of the geometric mean for eGFP:mCherry ratios on a single-cell level. The values were then normalized by dividing the average geometric mean of the experimental condition by the average geometric mean of the DMSO controls. Each experiment was performed in biological triplicate. In GraphPad Prism (v9.5.1), DC_50_ values were calculated using an inhibitor vs. response variable slope (four parameter) model.

### Immunoblots

HEK293T or K562 cells were plated at a density of 1.6 x 10^5^ cells/well in a 24-well plate coated with poly-D-lysine (Corning) in 1 mL media (DMEM + 10% FBS for HEK293T cells and RPMI + 10% FBS for K562 cells). After 16–18 hrs, cells were treated with either DMSO or the indicated concentration of small molecules to a 0.5% final concentration of DMSO. Plates were incubated at 37 °C, 5% CO_2_ for 24 hours for all cell lines except endogenously tagged PLK1–SD40 HEK293T cells, which were incubated for 2 hours. Cells were washed with PBS and then lysed for 30 minutes at 4 °C in radioimmunoprecipitation assay (RIPA; 25 mM Tris-HCl, 150 mM NaCl, 1% NP-40, 1% sodium deoxycholate, 0.1% SDS, pH ~ 7.6) buffer containing 1 mM phenylmethylsulfonyl fluoride (PMSF; Sigma Aldrich) and 1 x EDTA-free protease inhibitor cocktail (Roche). Lysates were centrifuged for 20 min @18,000 x g to isolate the soluble fractions. Protein concentration in clarified lysates was quantified using Pierce BCA Protein Assay Kit (Thermo Scientific) according to the manufacturer’s protocol. Clarified lysates were mixed with NuPAGE LDS Sample Buffer (Thermo NP0007) containing 10 mM DTT and boiled for 10 minutes. Gel electrophoresis was performed on boiled samples using polyacrylamide gels (Invitrogen NP0329BOX) with equal amounts of total protein in each lane. Proteins were transferred to nitrocellulose membranes (Invitrogen IB23001) using the iBlot 2 (Invitrogen IB21001) P0 protocol. After transfer, membranes were blocked in blocking buffer (50 mM Tris-HCl, 150 mM NaCl, 0.5% Tween-20, 1% bovine serum albumin, pH 7.4) for 1 hr at RT and then incubated with primary antibody overnight at 4 °C in blocking buffer. The following primary antibodies were used for the indicated proteins at the reported dilutions: HA-tag (Cell Signaling, #3724; 1:1000), BRD4 long isoform (Cell Signaling, #13440; 1:1000), GAPDH (Cell Signaling, #5174; 1:5000), Beta tubulin (Bioss, bsm-33034M; 1:5000), PLK1 (Cell Signaling, #4513; 1:1000), GFP (Cell Signaling, #2956; 1:1000), Anti-TUB (Invitrogen, #PA531164, 1:1000). Following overnight incubation, membranes were washed 3x with TBST buffer (50 mM Tris-HCl, 150 mM NaCl, 0.5% Tween-20, pH 7.4). Membranes were incubated with 1:10,000 goat anti-rabbit 680RD (LI-COR, 926-68071) and/or goat anti-mouse 680RD (LI-COR, 926-68070) for 1hr at RT in blocking buffer. Membranes were washed 3x with TBST buffer and visualized on an Odyssey Imaging System (LI-COR).

### Degron purification for BLI and TR-FRET

SD40 and a two-zinc finger construct containing SD0 (residues 141-243(Δ197-238) of IKZF1 where residues 450-460 of ZFP91 are in place of amino acids 146-156 of IKZF1) (Table S2) were cloned into vectors enabling expression as a Avi- and His-tagged maltose binding protein (MBP) fusion in *E. coli* BL21(DE3) cells (NEB, C2527H). Colonies were seeded into LB media supplemented with kanamycin and grown overnight with shaking at 37 °C. The following day, cultures were back-diluted 1:200 in 2xYT medium supplemented with 50 μM zinc chloride and kanamycin. Cultures were grown with shaking at 37 °C until OD600 reached ~0.4-0.6. Cultures were cooled on ice for 20 min., and expression of degron–MBP–Avi–His fusion proteins was induced with 0.5 mM isopropylthiogalactoside (IPTG). Cultures were grown overnight with shaking at 16 °C. Cells were harvested by centrifugation at 4000 x g for 20 min. Pellets were resuspended in ice-cold lysis buffer (50 mM Tris-Hcl, 450 mM NaCl, 10 mM imidazole, 50 μM ZnCl_2_, 20% glycerol, 3 mM TCEP, Roche cOmplete EDTA-free protease inhibitor tablet, pH 7.6) and lysed by 3 minutes of sonication (pulses were 3 seconds on followed by 3 seconds off). Lysates were clarified via centrifugation at 20,000 x g for 30 minutes. Clarified lysates were incubated with HisPur Ni-NTA resin (Thermo Fisher Scientific, 88221) for 1 hour with gentle rocking at 4 °C. Resin was washed with 20 column volumes of ice-cold wash buffer (50 mM Tris-Hcl, 300 mM NaCl, 25 mM imidazole, 50 μM ZnCl_2_, 10% glycerol, 3 mM TCEP, pH 7.6) followed by 10 column volumes of elution buffer (50 mM Tris-Hcl, 50 mM NaCl, 500 mM imidazole, 50 μM ZnCl_2_, 10% glycerol, 3 mM TCEP, pH 7.6). Pooled elution fractions were buffer exchanged into storage buffer (25 mM HEPES, 150 mM NaCl, 50 μM ZnCl_2_, 3 mM TCEP, 10% glycerol, pH 8.2) and concentrated using 30 kDa MWCO Amicon Ultra-15 Centrifugal Filter Unit (Millipore Sigma, UFC903096). Samples concentrations were determined using Bradford assay.

### Degron biotinylation for BLI and TR-FRET

Degron protein samples were buffer exchanged using 30 kDa MWCO Amicon Ultra-15 Centrifugal Filter Unit (Millipore Sigma, UFC903096) into biotinylation buffer (50 mM bicine, 50 mM NaCl, 1 mM TCEP, 50 μM ZnCl_2_, pH 8.3). 40 nmol of protein sample was used as input. Biotinylation reactions were set up according to manufacturer’s protocols (Avidity, BirA500-RT). Following 1-2 hours of incubation at RT, samples were buffer exchanged into storage buffer (25 mM HEPES, 200 mM NaCl, 50 μM ZnCl_2_, 1 mM TCEP, 10% glycerol, pH 7.4) via FPLC using a Superdex^™^ 200 Increase 10/300 GL column (Cytiva). Fractions were analyzed via SDS-PAGE and samples containing the biotinylated degron sample were concentrated using a 30 kDa MWCO Amicon Ultra-15 Centrifugal Filter Unit. Pooled, concentrated protein samples were flash-frozen in liquid nitrogen and stored at −80°C for later use.

### CRBN (for BLI, TR-FRET, and EM) and degron (for EM) expression and purification

His_6_-tagged hsDDB1^ΔBPB^ (Uniport entry Q16531, residues 396–705 replaced with a GNGNSG linker) and FLAG and Spy-tagged hsCRBN (Uniport entry Q96SW2, full length) were cloned into pAC8-derived vectors (87) and recombinantly expressed using the baculovirus expression system (Invitrogen). To generate baculovirus, 1 μg of pAC8-derived plasmids was co-transfected with linearized baculoviral DNA in *Spodoptera frugiperda* Sf9 cells grown in ESF 921 medium (Expression Systems) at a density of 0.9×10^6^ cells/mL. Following 5 days of incubation without shaking at 27 °C, the supernatant was collected and a fresh batch of Sf9 cells at a density of 1×10^6^ cells/mL was infected with 1.5% (v/v) baculovirus for viral amplification. Viral titer and volume were increased by another two rounds of amplification in Sf9 cells for 3 days each incubated at 27 °C with shaking at 120 rpm. For protein expression, *Trichoplusia ni* High Five cells grown in SF-4 Baculo Express IC medium (BioConcept) at a density of 2×10^6^ cells/mL were co-infected with 1.5% (v/v) each of DDB1- and CRBN-expressing baculoviruses. After 40 hours of incubation at 27 °C with shaking at 160 rpm, High Five cells were collected by centrifuging for 15 min at 3,500×g.

Avi–His_6_–MBP–FLAG-tagged SD40 was cloned into a pET expression vector. For expression, *E. coli* LOBSTR cells (Kerafast) were transformed and grown in TB medium (ThermoFisher) at 37 °C. Upon achieving an OD_600_ of 1.4, cells were induced with 0.5 mM isopropyl β-D-1-thiogalactopyranoside (IPTG). After 4 hours, the culture was centrifuged for 15 min at 4,000 x g and the pellet collect and frozen at −80 °C.

All protein purification steps were done at 4 °C or on ice. Cells were resuspended in lysis buffer [50 mM Tris/HCl pH 8.0, 200 mM NaCl, 0.1% (v/v) Triton X-100, 1 mM tris(2-carboxyethyl)phosphine (TCEP), 1 mM phenylmethylsulfonyl fluoride (PMSF), and a cocktail of protease inhibitors; for SD40, 50 μM ZnCl_2_ and 10% (v/v) glycerol were also added] and lysed on ice by sonication. The lysate was separated using ultracentrifugation at 185,511×g for 1 hour and the soluble fraction was treated with Benzonase at room temperature for 15 min and filtered through 0.45 μm membrane. For DDB1^ΔBPB^•CRBN, the lysate was applied to anti-DYKDDDDK G1 Affinity Resin (Genscript) and eluted with 150 μg/mL FLAG peptide. For SD40, the lysate was applied to Ni-charged resin (Genscript). For BLI experiments, DDB1^ΔBPB^•CRBN tags were cleaved by incubating overnight at 4 °C with 3% (w/w) TEV protease. The sample was then diluted to 66.7 mM NaCl with 50 mM Tris/HCl pH 8.5, 1 mM TCEP buffer and applied to a Poros 50 HQ anion exchange column (ThermoFisher) and then eluted in 50 mM Tris/HCl pH 8.5, 1 mM TCEP buffer containing increasing concentrations of NaCl from 25 mM to 1 M. Eluted fractions were pooled and concentrated using a 30 kDa MWCO centrifugal concentrator. For SD40, the lysate was applied to high affinity Ni-charged resin (Genscript) and eluted with increasing concentrations of imidazole (100 to 500 mM). Eluted fractions were pooled and concentrated using a 10 kDa MWCO centrifugal concentrator (Sartorius).

Next, size exclusion chromatography was performed using a Superdex200 column (GE Healthcare) for DDB1^ΔBPB^•CRBN or a Superdex75 column (GE Healthcare) for SD40 in a buffer containing 25 mM HEPES/NaOH 7.4, 200 mM NaCl, and 1 mM TCEP; for SD40, 50 μM ZnCl_2_ and 10% (v/v) glycerol were also added. For DDB1^ΔBPB^•CRBN, only the fractions on the left of the main peak were collected, pooled, and concentrated to 5.34 mg/mL (35.37 μM) for cryoEM and 3.98 mg/mL (27.2 μM) for BLI experiments. For SD40, the fractions were collected, pooled, and concentrated to 9.42 mg/mL (186.9 μM) for cryoEM experiments. The concentrated purified samples were aliquoted, flash frozen in liquid N_2_, and stored at −80 °C. For TR-FRET, FLAG-eGFP-hsCRBN was purified by the same procedure as described above for FLAG and Spy-tagged hsCRBN.

### Biolayer interferometry (BLI)

BLI experiments were performed similarly to previously described methods (12). The Octet^®^ System (Sartorius) was used for all BLI experiments. All binding experiments were performed in black 96-well plates (VWR, 76221-764) in 250 μL/well BLI buffer: 25 mM HEPES, 200 mM NaCl, 1 mM TCEP, 0.1% v/v Tween20, 0.05 mg/mL BSA, 100 μM of indicated molecular glue compound at a final DMSO concentration of 1%, pH 7.4. All binding measurements were conducted with shaking (1,000 rpm) at 30°C. Biotinylated degron sample was immobilized on Streptavidin-coated optical probes (Sartorius, 18-5019) at a concentration that produced a steady, linear signal during loading (50-400nM). To generate analyte samples, DDB1-CRBN was precomplexed in BLI buffer for 30 min at RT. Pre-complexed sample was diluted 3-fold for a total of 3-5 concentrations. Baseline steps were performed in the exact buffer used for analyte and ligand preparation to ensure there were no effects from the addition of DMSO and the small molecule. Data were analyzed using the Octet data analysis software. All data were analyzed using a global fit analysis for a 1:1 binding model. Reported K_D_-values were calculated using the determined k_on_ and k_off_ values and validated for accuracy by comparing to the steady-state analysis.

### Time-resolved fluorescence resonance energy transfer (TR-FRET) assays

To measure compound EC_50_ values: 200 nM biotinylated SD0 or SD40, 200 nM CRBN–eGFP•DDB1^ΔBPB^, and 2nM terbium (Tb)-coupled streptavidin (88) were mixed in a buffer containing 50 mM HEPES/NaOH pH 8.0, 200 mM NaCl, 1 mM TCEP, 0.1% (v/v) pluronic acid, and 0.5% BSA (w/v) [mixture 1]. For each data point, 15 μL of the mixture 1 was added to a well in 384-well low volume round bottom microplate (Corning) and increasing concentrations of the compounds were dispensed using a D300e Digital Dispenser (HP) and normalized to 2% (v/v) DMSO. All steps were carried out on ice, and to avoid photobleaching samples were protected from light for steps involving CRBN-eGFP. Using a PHERAstar FS microplate reader (BMG Labtech), Tb was excited at 337 nm and emissions from Tb at 490 nm and from eGFP at 520 nm were measured. In each cycle, to minimize background, the emission signal was integrated in the window of 70–600 μs after the excitation pulse. The evolution of the emissions was monitored over 60 cycles each separated by 109 s. Background fluorescence due to the compounds was calculated by repeating the assay without CRBN–eGFP•DDB1^ΔBPB^ and subtracting the signal for the corresponding compound concentration. The ratio of emissions at 520/490 from the last 5 cycles was averaged as the final TR-FRET reading. In GraphPad Prism (v10.1.1), EC_50_ values were calculated using an agonist vs. response variable slope (four parameter) model.

To measure ternary complex K_D_ values: 400 nM biotinylated SD0 or SD40, 100 μM compound, and 4 nM of Tb-coupled streptavidin were mixed in the buffer as described above [mixture 2]. Serial dilutions of CRBN–eGFP•DDB1^ΔBPB^ from 9.8 nM to 1.25 μM were prepared in the same buffer [mixture 3]. 7.5 μL each of mixtures 2 and 3 were added to each well in a 384-well microplate and incubated. To subtract background signal from excess compound concentration, the reading from zero CRBN concentration was subtracted. To subtract background emissions from increasing concentrations of eGFP, emission values from a blank sample containing the same concentration of CRBN–eGFP•DDB1^ΔBPB^ but no compound was subtracted from each reading. TR-FRET readings were taken in the manner described above. In GraphPad Prism (v10.1.1), K_D_ values were calculated using specific binding with Hill slope model.

### Prime editing in HEK293T cells

Twin prime editing experiments were conducted as previously described (30, 54, 75). Briefly, HEK293T cells were seeded in 96-well plates (Corning) at a density of 15k cells in 100 μL DMEM + 10% FBS in each well. After 16–20 hours, cells were transfected at approximately 60% confluency with 0.5 μl of Lipofectamine 2000 (Thermo Fisher Scientific), according to the manufacturer’s protocols, and 250 ng of prime editor plasmid DNA, 33 ng of pegRNA 1 plasmid DNA and 33 ng of pegRNA 2 plasmid DNA. Cells were cultured for four days after transfection. Media was removed, and genomic DNA was extracted by the addition of 100 μL freshly prepared lysis buffer (10 mM Tris-HCl, PH 7.5; 0.05% SDS 25 μg/mL of proteinase K (ThermoFisher)) directly into each well. The mixture was incubated in the tissue culture plate at 37 °C for 1–2 hours, then transferred to a PCR plate and incubated at 80 °C for 30 minutes to inactivate enzymes.

### High-throughput DNA sequencing of genomic DNA samples

Targeted genomic sites were amplified from genomic DNA and sequenced on an Illumina Miseq as previously described (30). Briefly, primers containing Illumina forward and reverse adapters (Table S4) were used for a first round of PCR (PCR1) to amplify a genomic region of interest under the following conditions: 0.5 μM each forward and reverse primer, 1 μL of genomic DNA, and 10 μL PhusionU Green Multiplex PCR Master Mix in a 20-μL reaction. PCR reactions were carried out as follows: 98 °C for two minutes and then 30 cycles of 98 °C for 10 seconds, 61 °C for 20 seconds, 72 °C for 30 seconds, followed by a final 72 °C extension for two minutes. A second round of PCR (PCR2) was conducted to add a unique Illumina barcode pair to each sample under the following conditions: 0.5 μM each forward and reverse primer, 1 μL unpurified PCR1, and 10 μL PhusionU Green Multiplex PCR Master Mix in a 20-μL reaction. PCR reactions were carried out as follows: 98 °C for two minutes and then 10 cycles of 98 °C for 10 seconds, 61 °C for 20 seconds, 72 °C for 30 seconds, followed by a final 72 °C extension for two minutes. PCR2 products were pooled by common amplicon and purified by electrophoresis on a 1% agarose gel using a QIAquick Gel Extraction Kit (Qiagen), eluting with 40 μL water. DNA concentration was measured by fluorometric quantification (Qubit, ThermoFisher) and sequenced on an Illumina MiSeq instrument using Illumina MiSeq control software (version 3.1), according to the manufacturer’s protocols.

Sequencing reads were demultiplexed using MiSeq reporter (Illumina). Alignment of amplicon sequences to a reference sequence was performed using CRISPResso2 (89). CRISPResso2 was run in HDR mode using the desired SD40-containing allele as the expected allele (e flag). Editing yield was calculated as: [no.of reads with the desired edit that do not contain indels]÷[no.of total reads]. Indels were quantified as: [no.of reads aligning to either the desired edit or the wild-type allele containing an insertion or deletion]÷[no.of total reads].

To minimize PCR bias, genomic DNA from optimal editing conditions at PLK1 in HEK293T cells (editor PE6c (54), pegRNAs 313A and 314C (Table S3)) and BRD4 in K562 cells (editor PE6c, pegRNAs 327K and 328B (Table S3)) (Fig. 3G) were re-sequenced in a three-step PCR protocol with unique molecular identifiers (UMIs) as previously described (75). In brief, the genomic DNA was first linearly amplified (PCR0) with only a forward primer containing a 15-nt UMI (Table S4) under the following conditions: 0.1 μM forward primer, 1 μL genomic DNA, and 10 μL PhusionU Green Multiplex PCR Master Mix in a 20-μL reaction. PCR reactions were conducted as follows: ten cycles of 98 °C for 1 minute, 61 °C for 25 seconds, 72 °C for 1 minute. PCR products were purified by 1.8x AMPure beads (Beckman Coulter) according to the manufacturer's protocol and eluted in 20 μL nuclease-free water. PCR1 was carried out as before but using 1.5 μL of purified PCR0 as template and a forward primer that anneals outside the UMI (UMI-fwd, Table S4). PCR2 and high-throughput sequencing were carried out as before. Raw sequencing reads were UMI deduplicated using AmpUMI (90) before analyzing with CRISPResso2 as before.

### PLK1–SD40 HEK293T cell line generation

HEK293T cells were seeded in a 48-well plate (Corning) at a density of 30k cells in 250 μL DMEM + 10% FBS in each well. After 16–20 hours, cells were transfected at approximately 60% confluency with 1 μL of Lipofectamine 2000 (Thermo Fisher Scientific), according to the manufacturer’s protocols, and 750 ng of PEmax (91) plasmid DNA, 125 ng of pegRNA 277F plasmid DNA (Table S3) and 125 ng of pegRNA 278C plasmid DNA (Table S3). Cells were cultured for three days after transfection, then replated in 96-well plates at a density of 0.5 cells per well. Single-cell colonies were evaluated for homozygous knock-in of SD40 using the high-throughput DNA sequencing protocol above.

### Prime editing in K562 cells

K562 cells were nucleofected using a Lonza 4D-Nucleofector system. 200,000 cells per nucleofection were pelleted and washed with PBS, and washed cells were resuspended in nucleofection solution following the protocol for the Lonza SE Cell Line 4D-Nucleofector Kit. To each nucleofection cuvette was added 20 μL of resuspended cells, 750 ng of prime editor plasmid, 125 ng of pegRNA plasmid 1, 125 ng of pegRNA plasmid 2. The samples were electroporated using program FF-120, then allowed to incubate in the cuvette at room temperature for 10 minutes before adding the full nucleofection solution to 0.25 mL of media in a 48-well tissue culture plate (Corning). After three days, cells were pelleted in 1.5-mL tubes and media was removed. Genomic DNA was extracted by the addition of 100 μL freshly prepared lysis buffer (10 mM Tris-HCl, PH 7.5; 0.05% SDS 25 μg/mL of proteinase K (ThermoFisher)) directly into each well. The mixture was incubated at 37 °C for 1–2 hours, then transferred to a PCR plate and incubated at 80 °C for 30 minutes to inactivate enzymes. Genotyping of samples was performed as described above.

### SD40–BRD4 K562 cell line generation

The SD40–BRD4 K562 cell lines were generated by single-flap prime editing (27, 30). K562 cells were nucleofected as above with 750 ng of PEmax plasmid DNA, 250 ng of pegRNA DTN464 plasmid DNA (Table S3), and 83 ng of nicking sgRNA DTN487 plasmid DNA (Table S3). After 72 hours, cells were pooled, recounted, and nucleofected a second time according to the same protocol. Following another 72 hour incubation, cells were replated in 96-well plates at a density of 0.5 cells per well. Single-cell colonies were evaluated for homozygous knock-in of SD40 using the high-throughput DNA sequencing protocol above.

### Cas9-mediated HDR insertion of SD40 and FKBP12^F36V^

Double-stranded DNA donor templates (Table S2) were synthesized as gene blocks (IDT) and cloned into plasmids. Donor DNA was amplified from plasmids with Phusion U and appropriate primers (Table S4). Amplicons were purified with SPRI beads and resuspended in water to 1 μg/mL. Guide RNAs (Table S5) were synthesized (IDT) and resuspended in IDTE buffer to 50 μM. For each electroporation condition, RNP complex was prepared by mixing ALT-R v3 S.p. Cas9 nuclease (IDT, 120 pmol, 2.4 μL from 50 μM stock) and sgRNA (150 pmol, 3 μL) and allowed to incubate at room temperature for 20 minutes. Donor DNA (2.4 μg, 2.4 μL) and PBS (2.6 μL) were added to a total volume of 10 μL. Nucleofections were conducted in 20-μL cuvette strips with 200,000 HEK293T cells in 10 μL SF 4D-Nucleofector X Solution (Lonza). Immediately after electroporation 80 μL pre-warmed media (DEMEM + 10% FBS) aswas added to each cuvette. After 10 minutes, cells were transferred to 12-well plates in a total volume of 1 mL media and allowed to incubate for 72 hours. Media was removed, and genomic DNA was extracted by the addition of 250 μL freshly prepared lysis buffer (10 mM Tris-HCl, PH 7.5; 0.05% SDS 25 μg/mL of proteinase K (ThermoFisher)) directly into each well. The mixture was incubated in the tissue culture plate at 37 °C for 1–2 hours, then transferred to a PCR plate and incubated at 80 °C for 30 minutes to inactivate enzymes. Editing outcomes were determined by the high-throughput sequencing protocol with UMI deduplication as above. Reads were 200 base pairs; edits were considered correct if the 5' HDR junction was incorporated without indels.

### EM sample preparation and data collection

DDB1^ΔBPB^•CRBN, PT-179/pomalidomide, and SD40 were mixed and incubated on ice for 1 hour at final concentration of 10.5, 105, and 21 μM, respectively [mixture 1]. The dilution buffer used contained 20 mM HEPES/NaOH pH 7.0, 150 mM NaCl, and 3 mM TCEP. DMSO concentrations were kept below 2% (v/v). Grids (Quantifoil UltrAuFoil R 1.2/1.3) were glow discharged in PELCO easiGlow (20 mA, 120s, 39 Pa) and loaded onto a Leica EM-GP plunge freezer with chamber conditions of 10 °C and 90% relative humidity. Grids were first pre-incubated with 4 μL of 10 μM CRBN-agnostic ${\text{IKZF1}}_{140-196}^{Q146A,G151N}$ for 1 minute and then blotted from behind for 4 s with NCI Micro Whatman No.1 filter paper. Immediately, 4 μL of mixture 1 diluted 10-fold—with the dilution buffer during the 1-minute incubation time—was applied to the grids before blotting for 4 s and plunging into liquid ethane at −181°C.

Data sets were collected using SerialEM (92) (v4.1b) in either a Thermo Scientific Talos Arctica (DDB1^ΔBPB^•CRBN•pomalidomide•SD40) operated at 200 kV or a Thermo Scientific Titan Krios operated at 300 kV. The Titan Krios was equipped with a Gatan BioQuantum imaging filter (20 eV slit width). Movies were recorded on Gatan K3 direct electron detectors. For DDB1^ΔBPB^•CRBN•pomalidomide•SD40, nine movies (50 frames, 5 s exposure time) were recorded per stage position using beam shift at nominal magnifications of 36,000x. Defocus was varied between −0.8 and −2.0 μm, and total electron exposure was 53.80 e/Å^2^. For DDB1^ΔBPB^•CRBN•PT-179•SD40, 18 movies (2 per hole, 50 frames, 2.5 s exposure time) were recorded in 9 holes per stage position at a magnification of 105,000x and a total electron exposure of 52.0 e/Å^2^. The defocus was varied between −0.8 and −2.0 μm.

### Data processing and model building

Relion-4 (93) and cryoSPARC (v4.2.0) (94) were used for all processing steps. All resolutions given are based on the Fourier shell correlation (FSC) 0.143 threshold criterion (95, 96).

For the DDB1^ΔBPB^•CRBN•pomalidomide•SD40 data set, 2524 movies were corrected for beam-induced motion and CTF was estimated on-the-fly in cryoSPARC live. Low-quality movies were rejected, and 1,254,659 particles were extracted (1.59 Å/pixel) from the 1,212 remaining micrographs after TOPAZ (v0.2.5) (97) particle picking. The movie acceptance criterion in cryoSPARC live was the following: defocus average between −0.4 and −3.1 μm, CTF fit better than 8.1 Å, max in-frame motion below 6.153, astigmatism angle between −89.9° and 174.5°, sample tilt between 0.1° and 27.1°, and total number of particles extracted above 609. The particles were cleaned up in three rounds of heterogeneous refinement (using one good initial volume and 4 decoys). The resulting 561,074 particles were re-extracted at 1.28 Å/pixel and classified using 3D variability (with a soft mask around CRBN) into 10 clusters. A single cluster (55,205 particles) with most pronounced density for the zinc finger was used in a final homogeneous refinement, yielding a reconstruction at global resolution of ~3.3 Å with a B factor of 120 Å^2^. Local refinement with a soft mask encompassing CRBN (soft padding: 12 pixels), followed by postprocessing using deepEMhancer (98), further improved the density for the zinc finger. Maps post-processed by cryoSPARC, maps filtered according to local resolution and maps post-processed with deepEMhancer (all deposited under EMD-41423 and EMD-41777) were used for model building. DDB1 (from PDB: 8G46 (99)), CRBN (PDB: 5FQD (8)) and a zinc finger (PDB: 6H0F (9)) were fit into the density in UCSF ChimeraX (v1.5) (100) and relaxed using ISOLDE (101). COOT (v0.9.8) (102) was then used to manually build the remaining parts of the zinc finger and to improve the model. The final model was protonated and a ligand restraint file was generated with phenix.ready_set (103), followed by refinement using phenix.real_space_refine (v1.19.2-4158) (104) using reference restraints for the zinc finger. Initially the model was refined against the unsharpened map with ADP refinement switched on, followed by refinements against the sharpened map without ADP refinement. The final model was deposited in the PDB as 8TNP.

16,340 movies were collected for the DDB1^ΔBPB^•CRBN•PT-179•SD40 data set and corrected for beam-induced motion on-the-fly in cryoSPARC live. CTF was estimated and low-quality movies (CTF fit worse than 5 Å and relative ice thickness outside 0.986 to 1.128) were rejected, leaving 12,642 micrographs from which 6,798,113 particles were picked using TOPAZ. The particles were split into 3 batches, and each batch was sent through 3 rounds of heterogeneous refinement. Particles from good classes were combined and further cleaned up in another round of heterogeneous refinement. A consensus refinement from the remaining 1,844,380 particles yielded a reconstruction at 2.9 Å. The particles were Fourier-cropped to a pixel size of 2.2 Å/pixel and imported (105) into Relion for 3D classification. In a first round, particles were classified with alignment into 20 classes (τ=20), and all good particles (1,690,591) were sent into a masked classification (10 classes, τ=50) without alignment using a soft mask around CRBN (soft padding: 12 pixels). This yielded several classes with diverging CRBN conformations, four of which were re-imported into cryoSPARC and used for Movie S1. The two classes showing the most pronounced zinc finger density were re-imported into cryoSPARC, re-extracted at 0.87 Å/pixel, and refined to an overall global resolution of ~2.4 Å (B factor=70 Å^2^) and ~2.5 Å (B factor= 71 Å^2^), respectively. Local refinements both with and without a CRBN focused mask (soft padded to 12 pixels and 15 pixels, respectively) and post-processing using deepEMhancer further improved the zinc finger density. Local motion correction and CTF refinement did not improve the resolution or quality of the reconstructions and were thus not performed. All final maps were deposited in the EMDB as EMD-41424/EMD-41778 and EMD-41425/EMD-41779 for conformations 1 and 2, respectively. The starting model was obtained from the DDB1^ΔBPB^ •CRBN•pomalidomide•SD40 model built previously, and chains fit into the density in UCSF ChimeraX (v1.5) and PT-179 was built from pomalidomide in PyMOL (v2.5). Next, the two conformations were individually relaxed into density using Rosetta (v3.13) (79) in dual space—torsion and Cartesian with penalties for deviation from ideal bond lengths and angles. The fit-to-density score term was upweighted to 35 to approximate density correlations with interpolations on a per-atom basis on a grid pre-computed from the input map density. The command line used was: rosetta_refinement_script.sh .

COOT was then used to manually build and refine the model. The final model was protonated and a ligand restraint file was generated with phenix.ready_set, followed by unrestrained refinement using phenix.real_space_refine (v1.20.1–4487) against the unsharpened map with ADP refinement turned on. The final models were deposited in the PDB as 8TNQ and 8TNR for conformations 1 and 2, respectively.

A small patch of unaccounted density remains, which most probably stems from the uncleaved His_6_–TEV sequence of the used DDB1 construct (fig. S12L). Data collection parameters and refinement statistics validated with MolProbity (106) and EMRinger (107) integrated in Phenix and are available in Table S6, local resolution ranges are given based on the 0-75% percentile in local resolution histograms (108), and conical directional FSCs were used to determine the resolution ranges due to anisotropy(109). Structural biology applications used in this project were compiled and configured by SBGrid (110).

### Interface analysis

Interface comparison was made with Rosetta (v3.13) by first truncating the C-terminal tail of SD40, reordering the fold tree such that the interface being compared (i.e. jump number) corresponds to that of CRBN•PT-179 and SD40, and then using the following command to obtain interface scores, buried interface solvent-accessible surface area, and shape complementarity scores (77): sasa_and_sc_calc_scripts.sh .

### Scoring interactions between residue pairs

Reversion mutations were scored using Rosetta (v3.13) by first relaxing the SD40 and SD40^F18Q^ with minimal movement using the command: relax_script.sh .

Residue-wise scoring of the relaxed structures was then performed and the scores corresponding to the interaction between SD40 residue 18 and PT-179 was then extracting using residue_energy_breakdown_script.sh .

Custom scripts used for structural modeling and analysis can be found at Zenodo (111).

## Supplementary Material

SM Text

Fig S26

Fig S24

Fig S23

Fig S22

Fig S21

Fig S20

Fig S19

Fig S18

Fig S17

Fig S16

Fig S15

Fig S14

Fig S13

Fig S12

Fig S11

Fig S10

Fig S9

Fig S8

Fig S7

Fig S6

Fig S5

Fig S4

Fig S3

Fig S2

Fig S1

movie s1

Data S1
