# Supplementary material for: Continuous evolution of compact protein degradation tags regulated by selective molecular glues
Source: Science. Author manuscript; Available in PMC 2024 Jun 26. (PMC11203266; doi:10.1126/science.adk4422)
Supplement: SM Text [file NIHMS1991970-supplement-SM_Text.pdf]

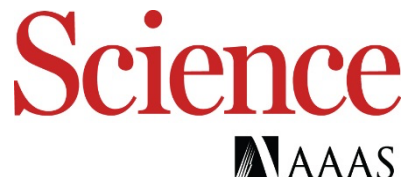

Supplementary Materials for

Continuous evolution of compact protein degradation tags regulated by selective molecular glues

Jaron A. M. Mercer[†], Stephan J. DeCarlo[†], Shourya S. Roy Burman[†], Vedagopuram Sreekanth, Andrew T. Nelson, Moritz Hunkeler, Peter J. Chen, Katherine A. Donovan, Praveen Kokkonda, Praveen K. Tiwari, Veronika M. Shoba, Arghya Deb, Amit Choudhary^{*}, Eric S. Fischer^{*}, David R. Liu^{*}

Corresponding author: drliu@fas.harvard.edu, eric_fischer@dfci.harvard.edu,
achoudhary@bwh.harvard.edu

The PDF file includes:

Figs. S1 to S26
Tables S1 to S7
References and Notes

Other Supplementary Materials for this manuscript include the following:

Movie S1
Data S1

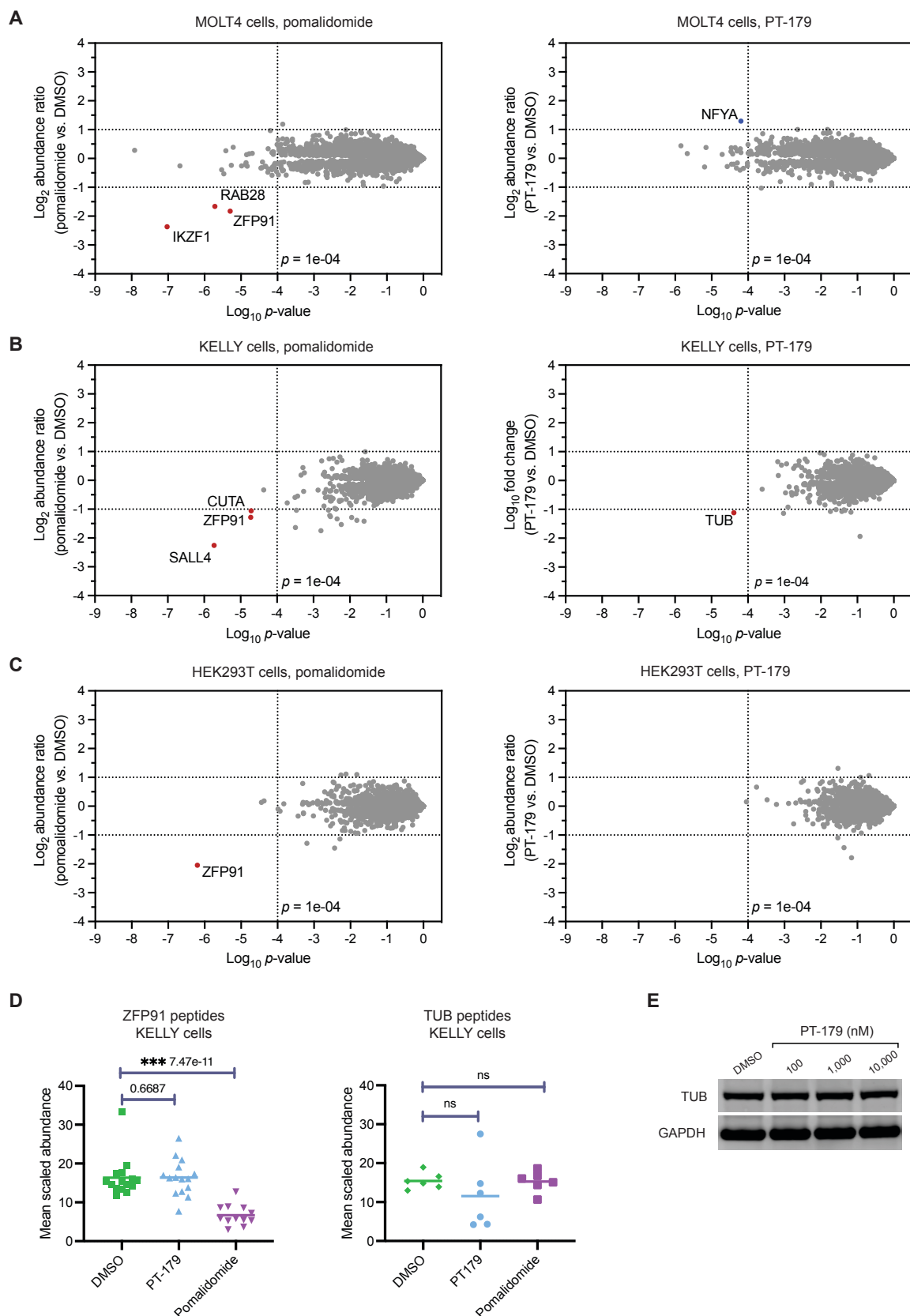

Fig. S1. PT-179 degrades no detected off-target neosubstrates in human cancer and immortalized cell lines. (A) Global proteomics by quantitative LCMS analysis of MOLT4 cells (6310 proteins detected), (B) KELLY cells (7259 proteins detected), and (C) HEK293T cells (7593 proteins detected) treated for

5 hours with pomalidomide (1 μ M) or PT-179 (1 μ M) compared to control cells treated with DMSO only. The only notable change in protein abundance following PT-179 treatment in KELLY cells was the protein TUB, which is not known to be a neosubstrate of IMiDs. TUB was confirmed to be a false positive by western blot shown in (E), below. **(D)** Mean scaled abundance of ZFP91 and TUB peptides under DMSO, PT-179 (1 μ M), and pomalidomide (1 μ M) treatment conditions in KELLY cells; *p*-values were calculated using a Welch's t-test. **(E)** Western blot of TUB in HEK293T cells indicates that PT-179 does not degrade TUB.

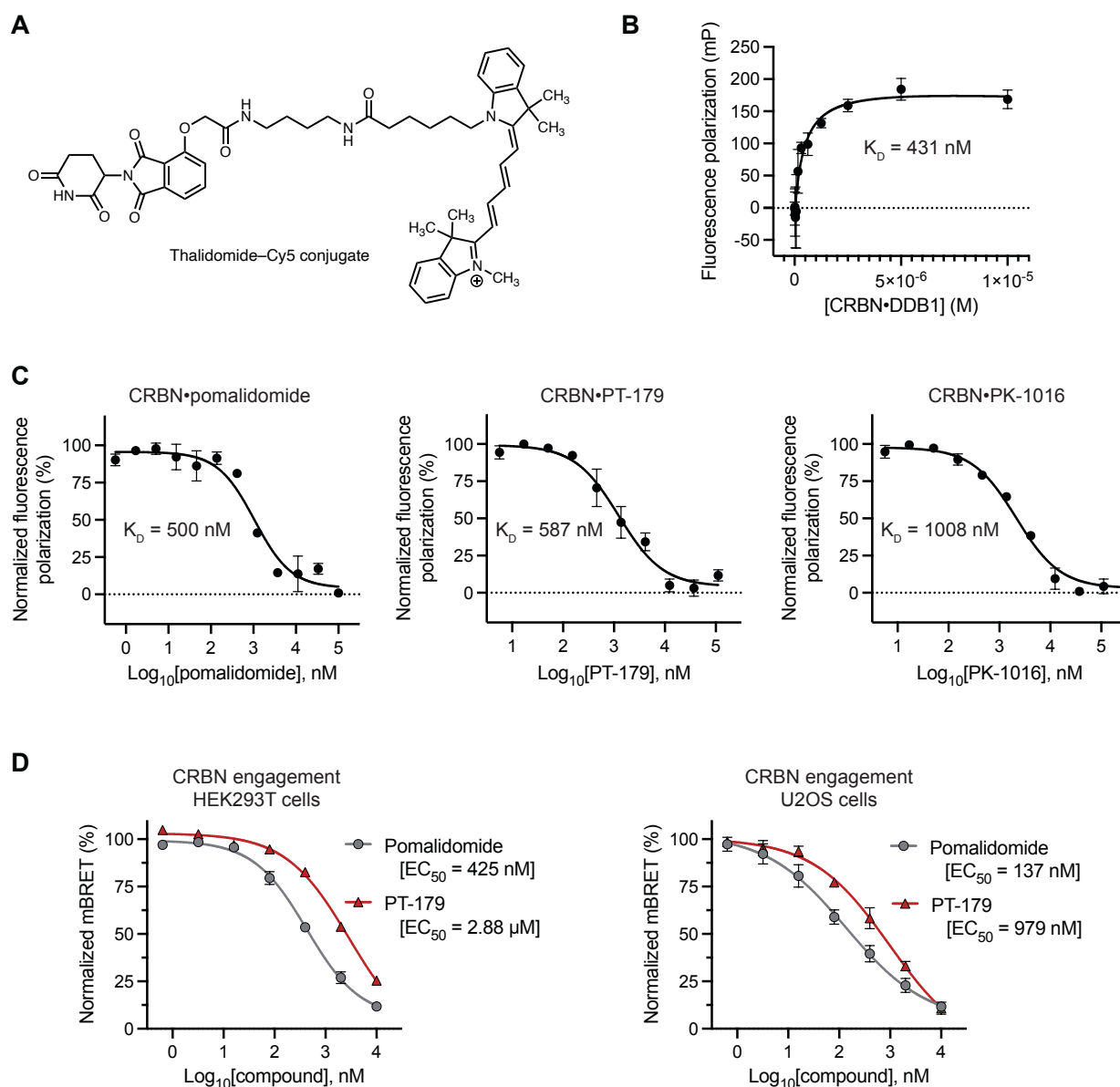

Fig. S2. PT-179 binds CRBN *in vitro* and in human cell tissue culture. **(A)** Structure of thalidomide–Cy5 conjugate for competitive fluorescence anisotropy. **(B)** Fluorescence polarization for thalidomide–Cy5 binding to DDB1•CRBN *in vitro*. **(C)** Competitive fluorescence anisotropy for pomalidomide, PT-179, and PK-1016 binding DDB1•CRBN *in vitro*. **(D)** CRBN engagement assay by bioluminescence resonance energy transfer (BRET) with pomalidomide and PT-179 in HEK293T cells (left) and U2OS cells (right). Values and error bars in (B–D) represent the mean and standard deviation of three replicates.

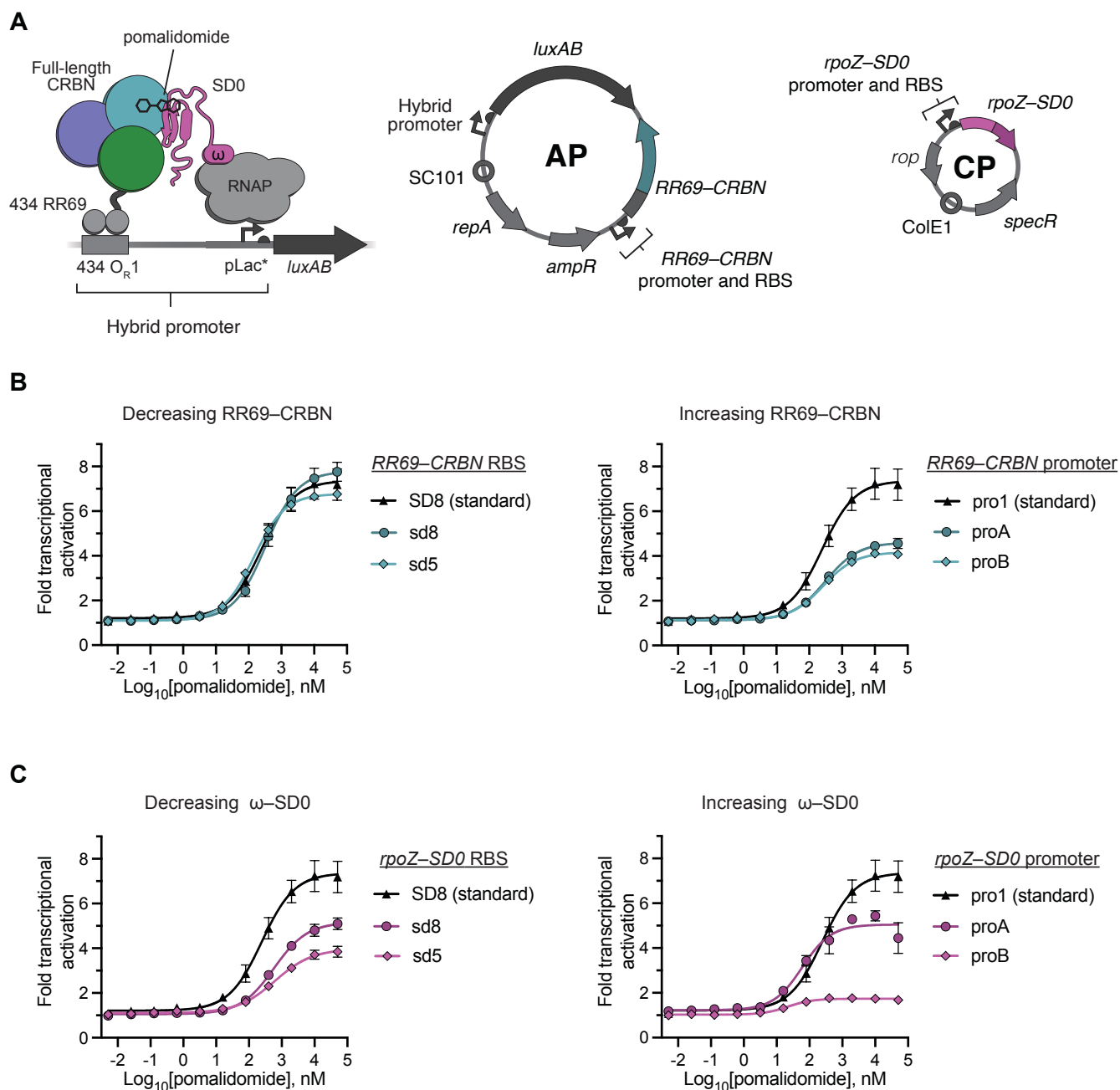

Fig. S3. The MG-PACE circuit is sensitive to the concentration of both protein components. **(A)** The active transcription complex (left) assembles at the hybrid promoter to drive expression of *luxAB* from the accessory plasmid (AP, center). Bait protein RR69-CRBN is encoded in a separate ORF on the AP. In transcriptional activation assays gene *rpoZ-SD0* (protein ω -SD0) is encoded on a complementary plasmid (CP, right). For evolution *rpoZ-deg* variants are encoded on selection phage (SP, not shown). **(B)** Decreasing (left) or increasing (right) expression of RR69-CRBN. **(C)** Decreasing (left) or increasing (right) expression of ω -SD0. Values and error bars in (B) and (C) represent the mean and standard deviation of three replicates, each normalized to a control treatment with DMSO only. Luminescence was measured one hour after treating cells with polymyxin B nonapeptide (3 μ g/ml) and pomalidomide at the indicated concentration. Relative RBS strengths (71): SD8 (1.0) > sd8 (0.20) > sd5 (0.048). Relative promoter strengths (113): pro1 (1.0) < proA (3.33) < proB (13.22).

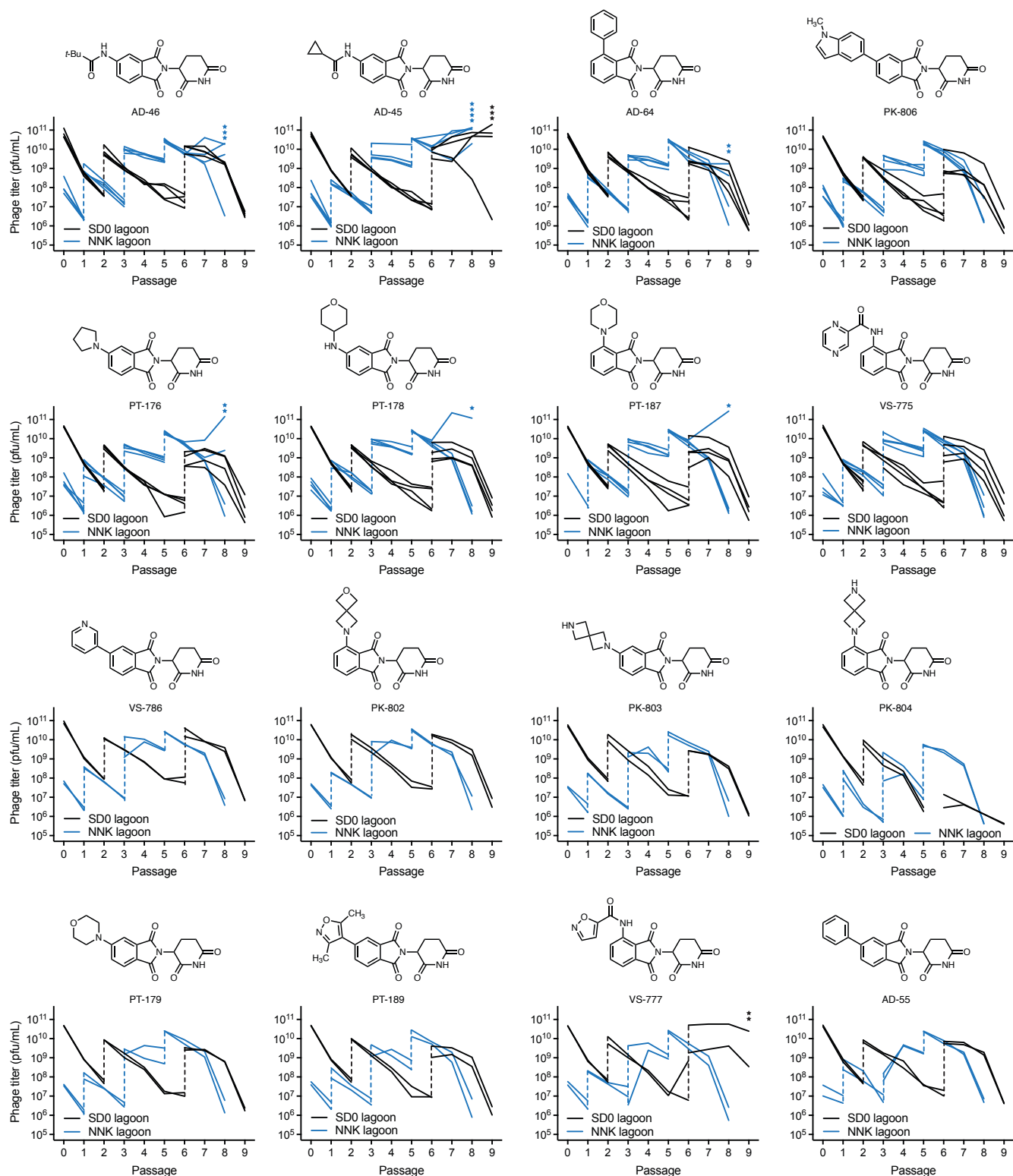

Fig. S4. PANCE on the CRBN_{CTD} circuit with IMiD derivatives. Black traces are lagoons seeded with SD0 phage; blue traces are lagoons seeded with the SD0 NNK library phage. Passages were 8–16 hours, and between passages surviving phage were diluted 1:50 into a new lagoon with fresh host cells and the indicated IMiD derivative (10 μ M). The final passage (passage 9 for SD0 lagoons and 8 for NNK lagoons) was a 1:1000 dilution. In most passages phage titer decreases, reflecting that SD0

cannot accommodate phthalimide ring substituents and thus insufficient pIII is produced from the MG-PACE circuit to support propagation. Vertical dashed lines represent neutral drift passages (1:50 dilution) in host cells that permit phage propagation in the absence of selection pressure to rescue phage titer and increase genetic diversity (38). Stars highlight lagoons that reached high phage titer after eight or nine passages.

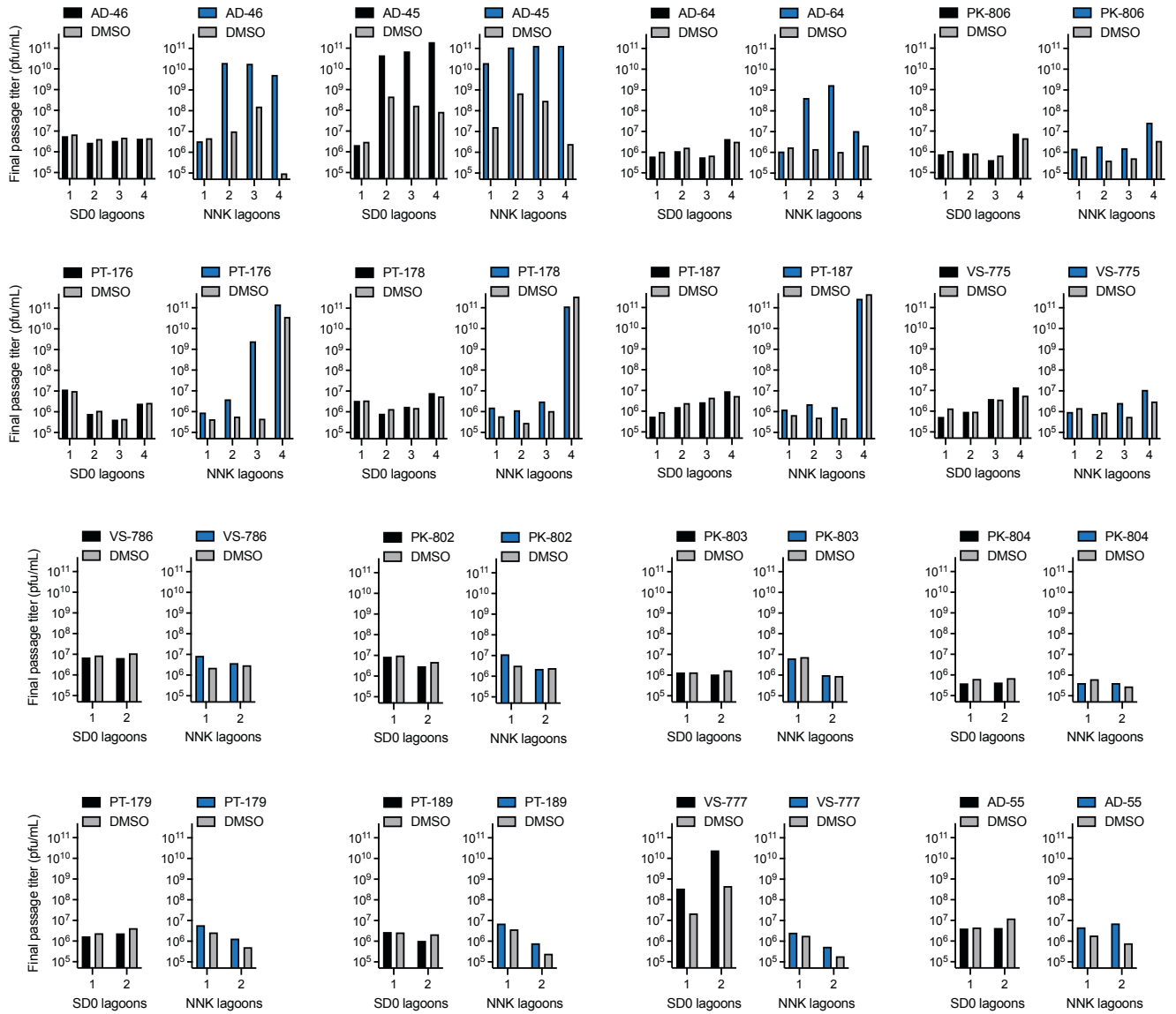

Fig. S5. IMiD derivative stepping-stone evolution results. The final PANCE passage was conducted with and without target IMiD derivatives in the lagoon media. Lagoons that reach high titer in media lacking IMiD derivatives harbor ‘cheating’ phage that evolved mechanisms to induce gene III expression in an IMiD-independent manner, for example by directly binding CRBN_{CTD}. High phage titers in the presence of IMiDs and low titers in the absence of IMiDs indicate successful evolution.

A

SD0 lagoons

	3	14	16	17	20	31	44	45	46	58
SD0:	V	E	P	L	E	N	P	F	K	D
AD-45 2						K	L			
						K	L			
						K	L			
					K	K	L			G
AD-45 3					K		L			
					K		L			
					K		L			
AD-45 4		F		F	K		L			
		F		F	K		L			
		F		F	K		L			
		F		F	K		L			
VS-777 1			S		K		T	V	*	-
		D	L		K		T	V	*	-
			S		K		T	V	*	-
VS-777 2	E		L		K					
			L		K					
			L		K					
			L		K					

B

SD0 NNK lagoons

	1	18	20	21	30	31	37	40	44	51
SD0:	F	Q	E	I	G	N	K	T	P	N
AD-46 2		M	P	V				M	L	
		M	P	V				M	L	
		M	P	V			N	M	L	
		M	P	V				M	L	
AD-46 3		M	P	V				M	L	
		M	P	V				M	L	
		M	P	V		K		M	L	
		M	P	V		K		M	L	
AD-46 4		I	P	V					L	
		I	P	V					L	
		I	P	V					L	
		I	P	V					L	K
AD-45 1		I	P	V					L	
		M	P	V				M	L	
		I	P	V					L	
		M	P	V				M	L	
AD-45 2	L	D	S	S						
		M	P	V				M	L	
		A	L	T						
		L	R	R						
AD-45 3		I	P	V					L	
		I	P	V					L	
		I	P	V	V				L	
		I	P	V					L	
AD-45 4		A	Y	P						
		I	P	V					L	
		I	P	V					L	
		I	P	V					L	
AD-64 2		M	P	V				M	L	
		M	P	V				M	L	
		M	P	V				M	L	
		M	P	V				M	L	
AD-64 3		M	P	V				M	L	
		M	P	V				M	L	
		M	P	V				M	L	
		M	P	V				M	L	
PT-176 3		I	P	V					L	
		I	P	V					L	
		I	P	V					L	
		I	P	V					L	

Fig. S6. Mutations in phage from non-cheating lagoons after PANCE with stepping-stone IMiD derivatives. **(A)** Mutations in phage from the five lagoons that reached high phage titer after nine passages. All were seeded with SD0 phage. **(B)** Mutations in phage from the ten lagoons seeded with SD0 NNK library phage that reached high titer after eight passages. Residues 18, 20, and 21 (highlighted in yellow) were diversified in the initial library; mutations highlighted in blue arose during PANCE.

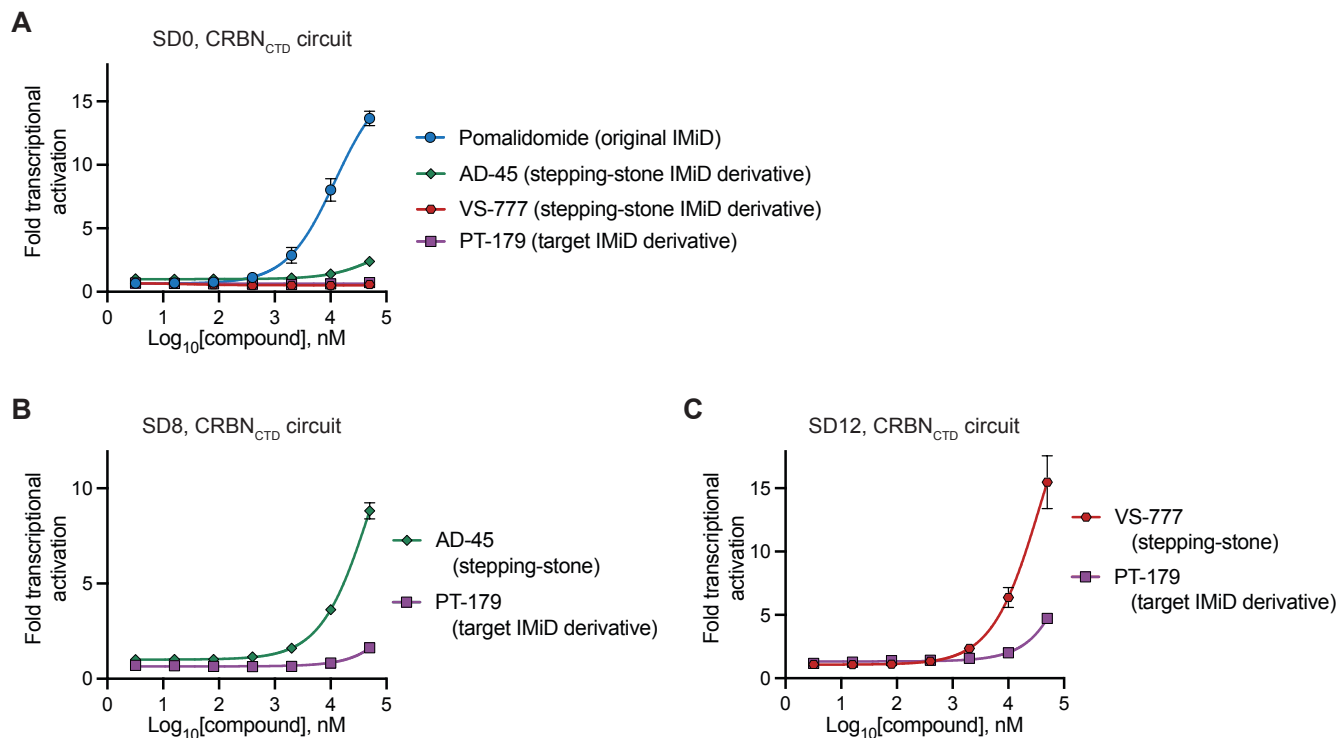

Fig. S7. MG-PACE circuit activation with degron variants evolved on stepping-stone IMiD derivatives. **(A)** CRBN_{CTD} circuit activation with SD0 by pomalidomide, target IMiD derivative PT-179, and stepping-stone IMiD derivatives AD-45 and VS-777. **(B)** PANCE-evolved variant SD8 activating the CRBN_{CTD} circuit in response to AD-45, which it evolved to bind, and PT-179. **(C)** Variant SD12 activating the CRBN_{CTD} in response to VS-777, which it evolved to bind, and PT-179. Values and error bars in (A–C) represent the mean and standard deviation of three replicates, each normalized to a control treatment with DMSO only. Luminescence was measured one hour after treating cells with polymyxin B nonapeptide (3 μ g/ml) and the indicated IMiD derivative.

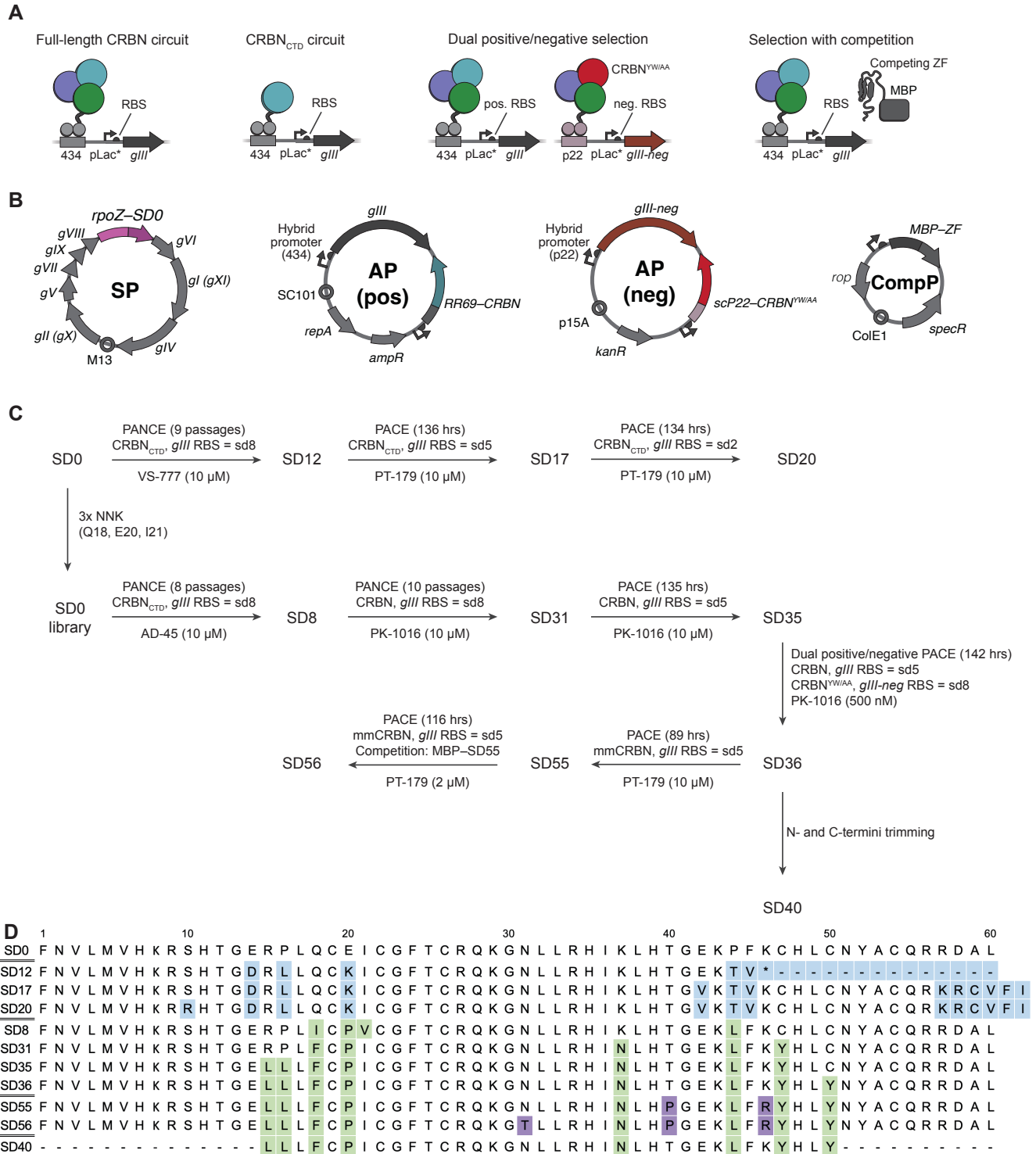

Fig. S8. Summary of degron evolution campaign. **(A)** MG-PACE selection circuits. **(B)** Selection phage (SP), accessory plasmid (AP), and competition plasmid (CompP) architectures. **(C)** Roadmap of all PANCE and PACE experiments in this work. SD8 and SD12 were isolated in a clonal phage population before the next evolution; in all other instances named degrons (e.g. SD17) are representative single genotypes from a pool of phage carried forward together. The SD0 library was cloned by site-specific mutagenesis using primers encoding NNK codon mixtures for Q18, E20, and I21. Relative RBS strengths (71): SD8 (1.0) > sd8 (0.20) > sd5 (0.048) > sd2 (0.010). **(D)** Genotypes of named degrons.

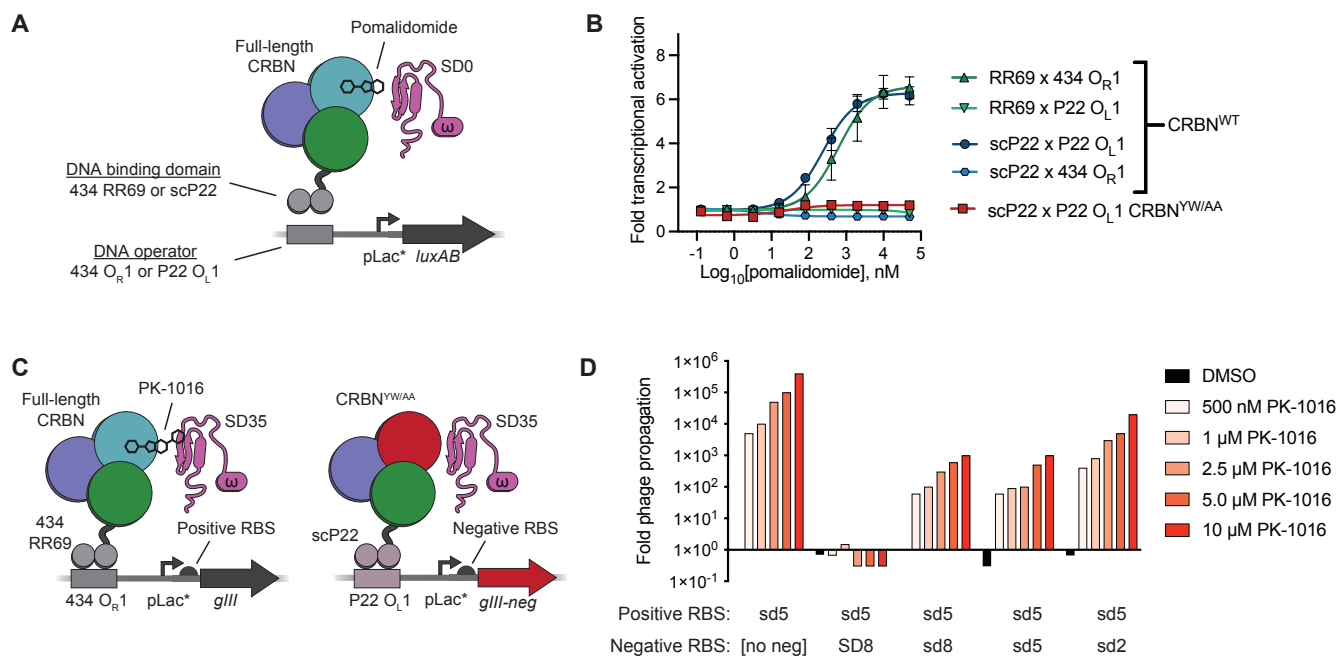

Fig. S9. Development of a negative selection PACE circuit with a single-chain variant of the P22 phage repressor c2 and CRBN^{Y384A,W386A}. **(A)** 434 RR69 and scP22 DNA-binding proteins with their respective operators in the full-length CRBN circuit. **(B)** Pomalidomide-induced transcriptional activation by 434 RR69–CRBN and scP22–CRBN paired with their cognate operators. The two circuits show no cross-talk. CRBN^{YW/AA}, which does not bind pomalidomide, does not activate the circuit. **(C)** MG-PACE circuits for simultaneous positive and negative selection. **(D)** Single PANCE passage phage propagation for SD35-encoding phage with simultaneous positive and negative selection. Values and error bars in **(B)** represent the mean and standard deviation of three replicates, each normalized to a control treatment with DMSO only. Luminescence was measured one hour after treating cells with polymyxin B nonapeptide (3 μg/ml) and pomalidomide.

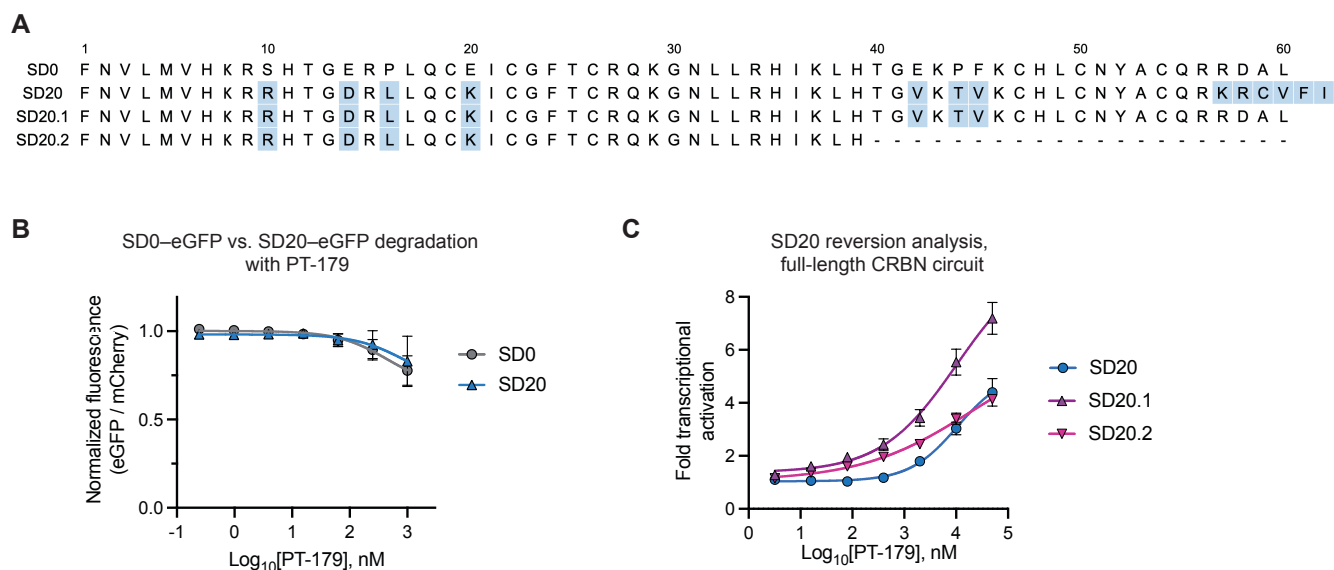

Fig. S10. Reversion of SD20 C-terminal mutations rescues full-length CRBN engagement. **(A)** Complete amino acid sequences of SD0, SD20, and SD20 variants. Mutations highlighted in blue. **(B)** SD0-eGFP and SD20-eGFP degradation by PT-179 (≥ 20 hrs treatment). SD20 shows no improvement as a degren in HEK293T cells. **(C)** PT-179-induced transcriptional activation in full-length CRBN circuit. Reversion of C-terminal mutations in SD20 (SD20.1) leads to a marked increase in signal. Removing the C-terminus of SD20 entirely (SD20.2) results in little-to-no improvement, supporting the relevance of favorable contacts with CRBN_{NTD} for ternary complex formation. Values and error bars in (B) and (C) represent the mean and standard deviation of at least three replicates, each normalized to a control treatment with DMSO only. Luminescence was measured one hour after treating cells with polymyxin B nonapeptide (3 $\mu\text{g/ml}$) and PT-179.

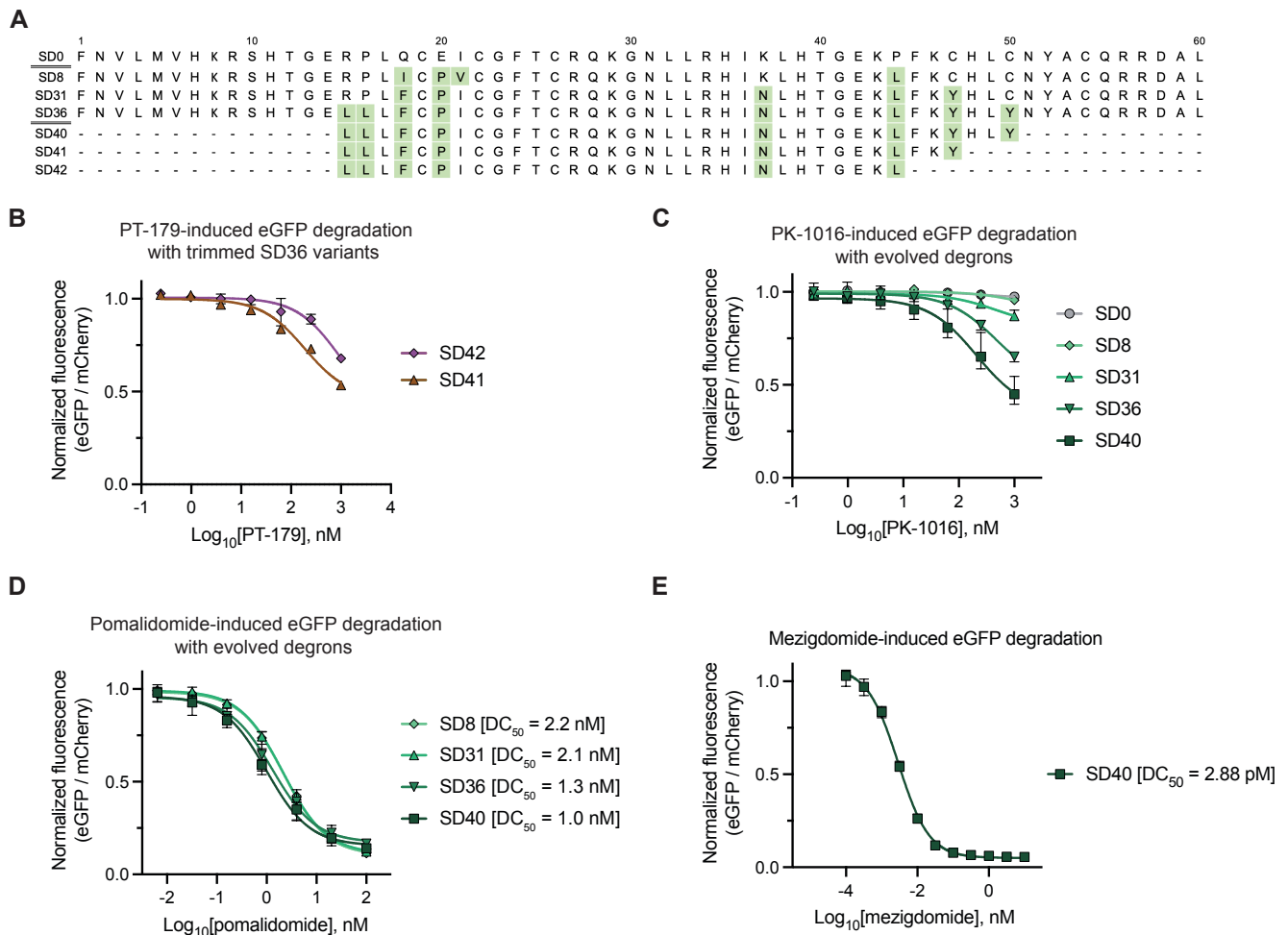

Fig. S11. Trimming of SD36 identifies a 36-amino acid minimal degron, SD40. **(A)** Complete amino acid sequences of evolved degrons and trimmed versions of SD36. Mutations compared to SD0 are highlighted in green. **(B)** PT-179-induced eGFP degradation with trimmed SD36 variants. Trimming the C-terminus of SD40 further leads to a marked reduction in potency. **(C)** PK-1016-induced degradation of eGFP tagged with evolved variants. PK-1016 displays poor activity as a degrader in mammalian cells. **(D)** Pomalidomide-induced degradation of eGFP tagged with evolved variants. Evolved variants retain compatibility with pomalidomide. **(E)** Mezigdomide-induced degradation of eGFP tagged with SD40. Values and error bars in (B–D) represent the mean and range of three or more replicates, ≥ 20 hrs treatment, all normalized to control treatment with DMSO only.

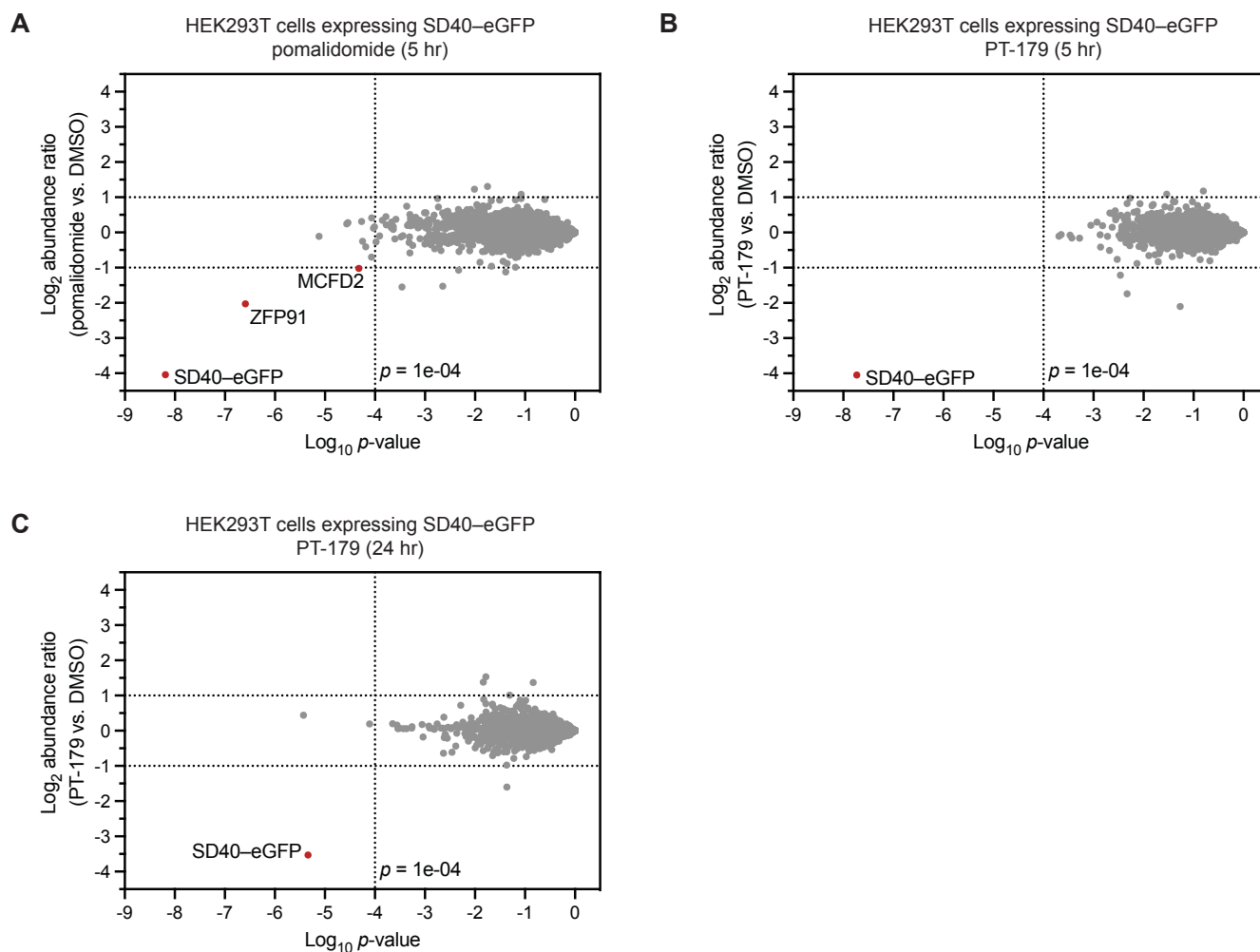

Fig. S12. SD40 does not create new PT-179 neosubstrates in HEK293T cells (A) Global proteomics by quantitative LCMS analysis of HEK239T cells expressing SD40-eGFP treated with pomalidomide (1 μ M, 5 hrs; 7,812 proteins detected), (B) PT-179 (1 μ M, 5 hrs; 7,793 proteins detected), and (C) PT-179 (1 μ M, 24 hrs; 7,830 proteins detected) compared to control cells treated with DMSO only.

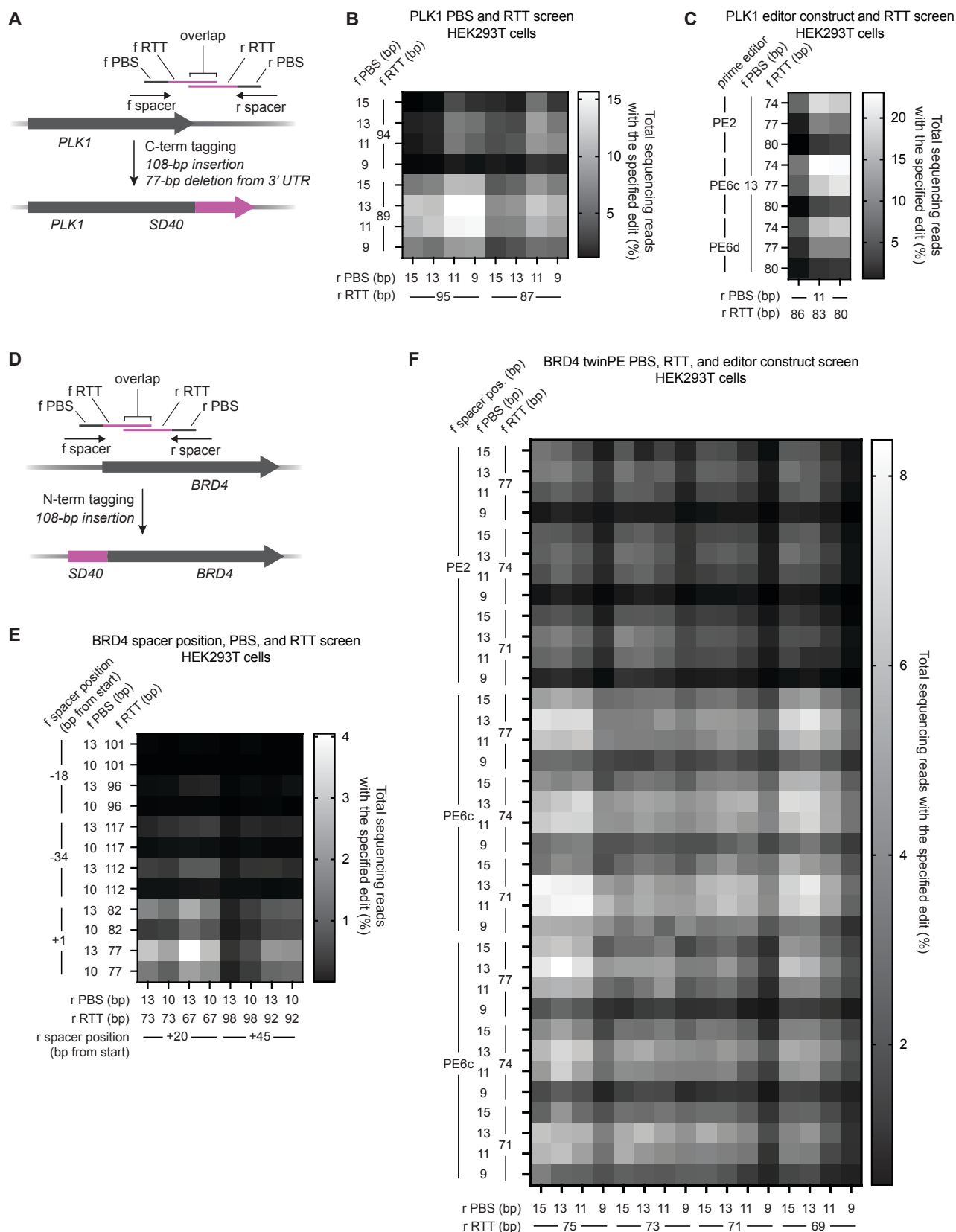

Fig. S13. In-frame insertion of SD40 in protein coding genes for endogenous protein tagging with twin prime editing. In twin prime editing (54, 75), two pegRNAs with forward (f) and reverse (r) spacers direct prime editor (Cas9–reverse transcriptase fusions) to target sites on opposite DNA strands. The

Cas9 domain of the prime editor nicks the genomic DNA, liberating a 3' end that anneals to a primer binding site (PBS) on the pegRNA. The engineered reverse transcriptase domain of the prime editor extends the nicked 3' end of target genomic DNA, copying an edit encoded by the reverse transcriptase templates (RTTs) on the pegRNAs. The resulting two complementary 3' flaps anneal and replace the original genomic sequence after cellular DNA repair. Optimizing twin prime editing requires the identification of optimal combinations of spacers, primer binding sites, reverse transcriptase templates, and prime editor constructs. **(A)** Twin prime editing to insert SD40 at the C-terminus of PLK1. **(B)** Initial PBS and RTT screen for PLK1 tagging. **(C)** Final RTT and prime editor construct screen for PLK1 tagging. **(D)** Twin prime editing to insert SD40 at the N-terminus of BRD4. **(E)** Initial spacer, PBS, and RTT screen for BRD4 tagging. **(F)** Final PBS, RTT, and prime editor construct screen for BRD4 tagging. Values in (B), (C), (E), and (F) represent the mean of three independent replicates.

Scarless degron tag insertion at BRD4 by HDR
HEK293T cells

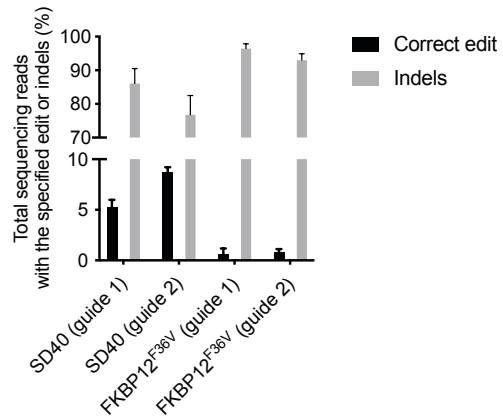

Fig. S14. In-frame insertion of SD40 and FKBP12^{F36V} at the BRD4 N-terminus by Cas9 nuclease-mediated HDR in HEK293T cells. Values and error bars represent the mean and standard deviation of three replicates.

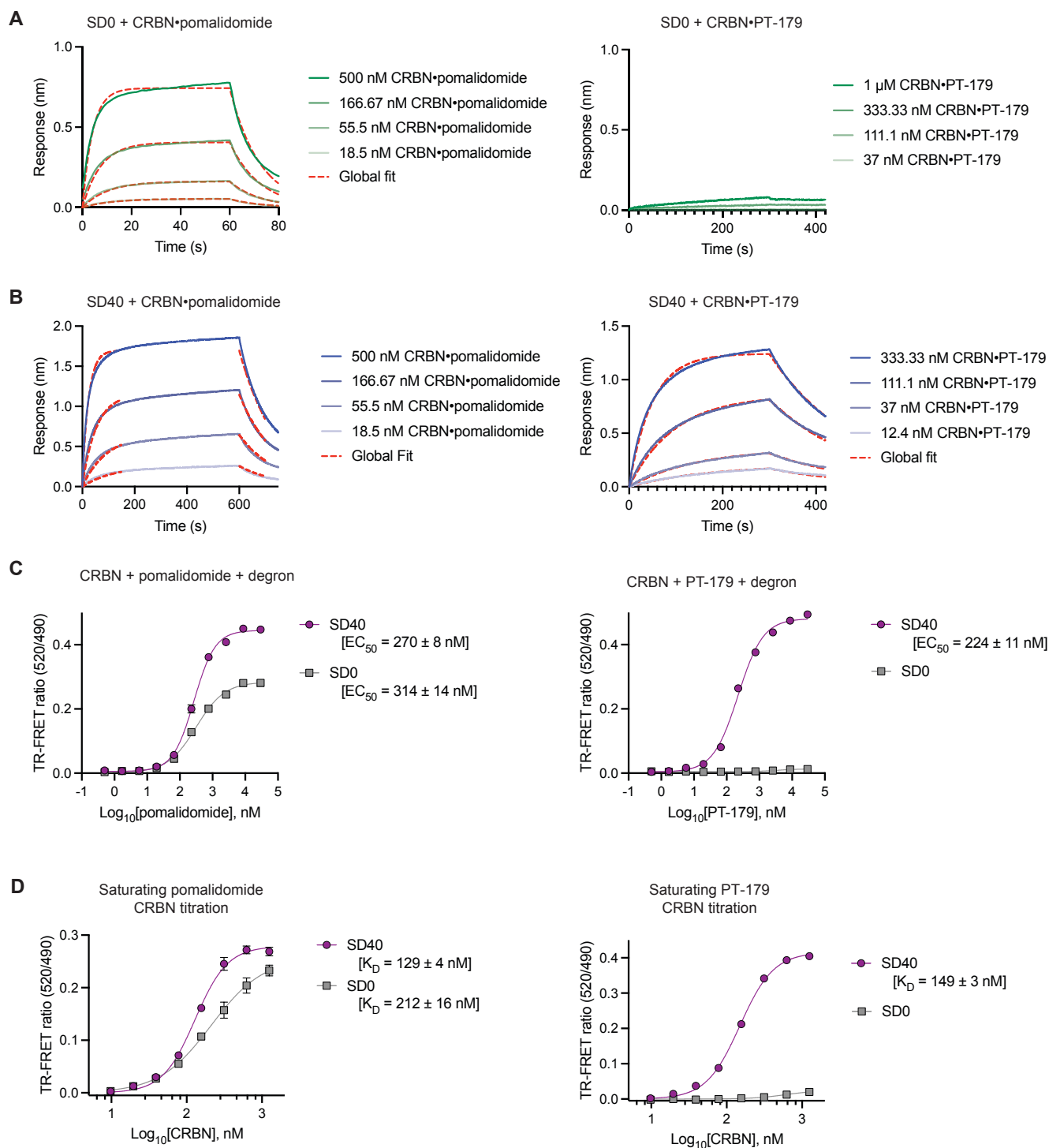

Fig. S15. SD40 potently binds CRBN•PT-179 in vitro. **(A)** Bio-layer interferometry measuring SD0 binding to CRBN•pomalidomide (left) and CRBN•179 (right). **(B)** Bio-layer interferometry measuring SD40 binding to CRBN•pomalidomide (left) and CRBN•179 (right). **(C)** Time-resolved fluorescence resonance energy transfer (TR-FRET) to measure pomalidomide (left) and PT-179 (right) EC₅₀ of ternary complex formation with SD0 and SD40. **(D)** TR-FRET to measure ternary complex dissociation constants for SD0 and SD40 in the presence of saturating pomalidomide (left) and PT-179 (right). Traces in (A) and (B) are representative single replicates. Values and error bars in (C) and (D) represent mean and standard deviation of three replicates.

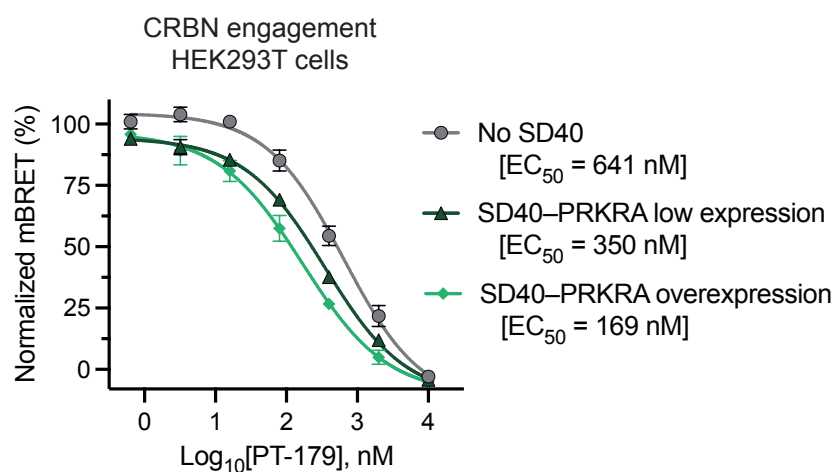

Fig. S16. Expressing SD40-GFP in HEK293T cells increases in-cell CRBN engagement by PT-179. CRBN engagement assay by bioluminescence resonance energy transfer (BRET) with PT-179 in HEK293T cells without SD40, with low SD40-PRKRA expression from a single-copy lentiviral integration, and with high SD40-PRKRA expression from plasmid transfection. Values and error bars represent the mean and standard deviation of three replicates normalized to control cells treated with DMSO only.

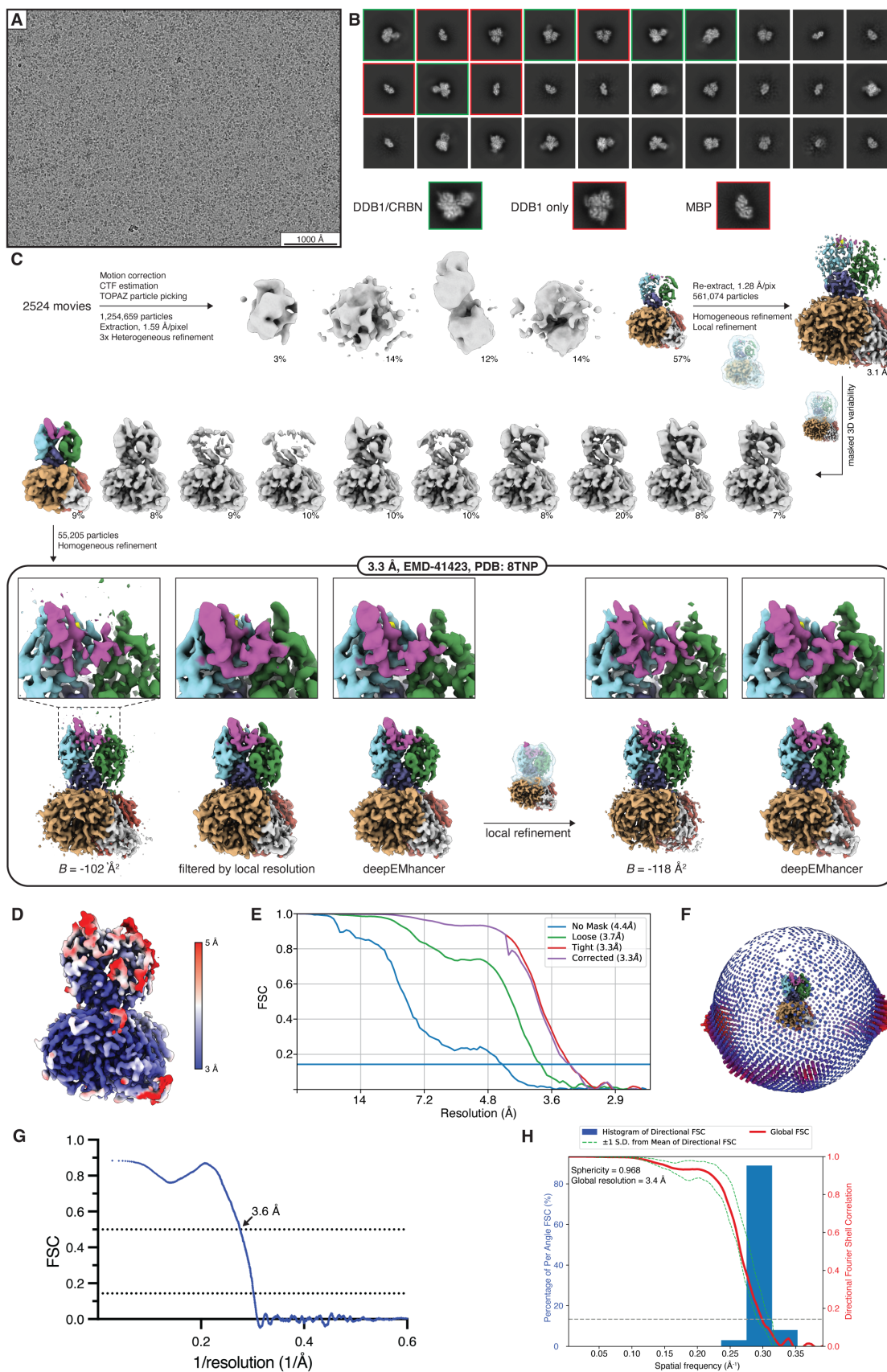

Fig. S17. Cryo-EM processing workflow and map and model statistics for DDB1^{ΔBPB}•CRBN•pomalidomide•SD40. (A) Representative raw micrograph, low pass-filtered to 5 Å. (B) Representative

2D classes, revealing distinct classes containing the ternary complex, DDB1 only classes, and classes focused on MBP. **(C)** Overview of processing workflow. Particles belonging to colored maps were kept for subsequent processing steps. The sharpening B -values are indicated where applicable, and final maps are shown at contour levels of 0.3 (main maps and maps filtered by local resolution), 0.42 (maps from local refinement), 0.09 (from DeepEMhancer) (99). **(D)** Main map colored according to local resolution. **(E)** FSC plot. **(F)** Viewing direction distribution. **(G)** Model-to-map FSC. **(H)** 3DFSC plot and directional distribution histogram.

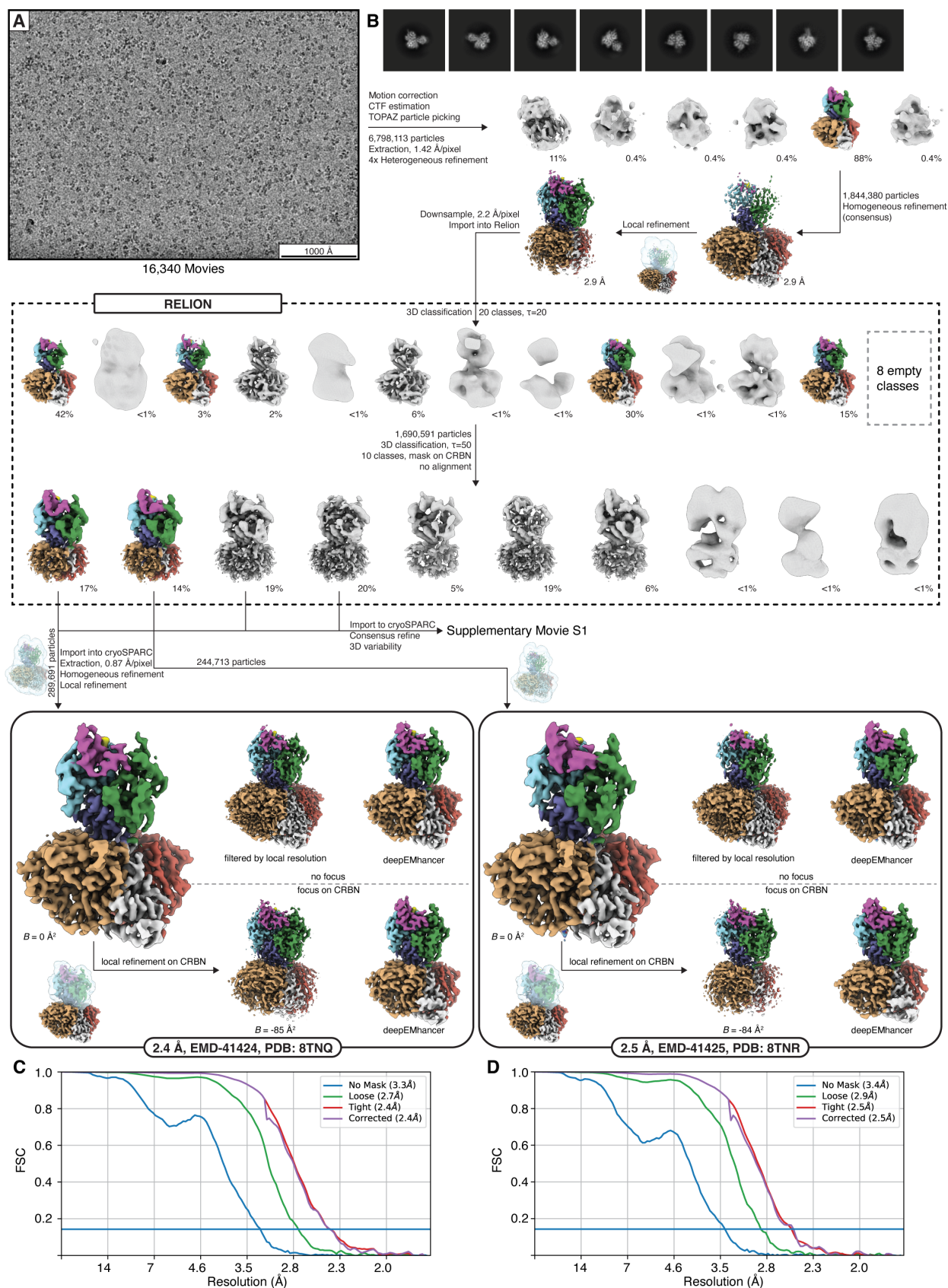

Fig. S18. Cryo-EM processing workflow for DDB1^{ΔBPB}•CRBN•PT-179•SD40. (A) Overview of processing workflow from micrograph (low pass-filtered to 5 Å, scale bar indicated) to final maps.

Two rounds of 3D classification were conducted in Relion, as indicated. Particles belonging to colored maps were kept for subsequent processing steps. The sharpening B -values are indicated where applicable, and final maps are shown at contour levels of 0.15 (main maps), 0.3 (filtered by local resolution), 0.42 (maps from local refinement), and 0.09 (from DeepEMhancer) (99). **(B)** Representative 2D classes. **(C, D)** FSC plots for deposited main maps (EMD-41424 (A), EMD-41425 (B)).

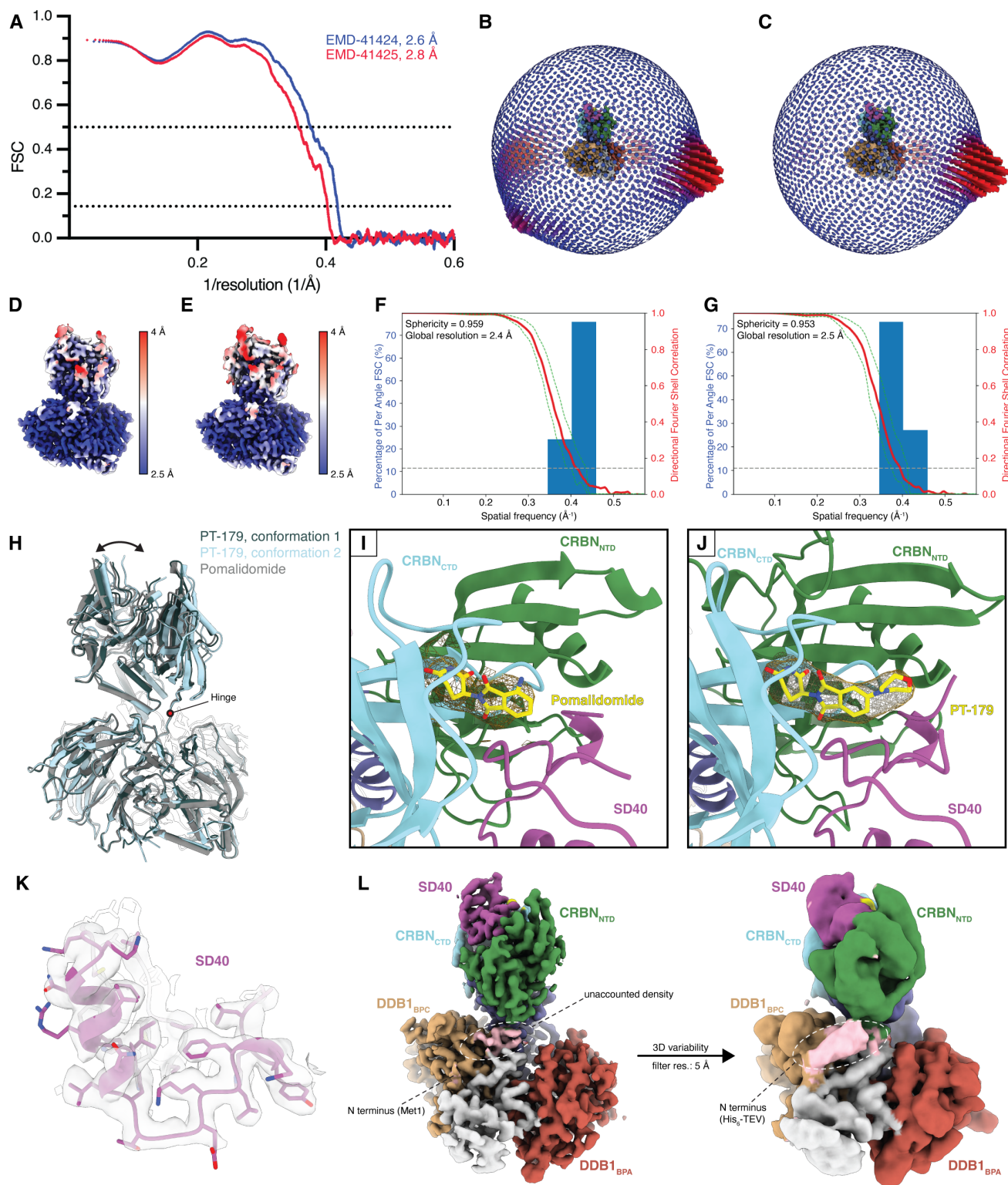

Fig. S19. Map and model validation for the DDB1^{ΔBPB}•CRBN•PT-179•SD40 structure. (A) Model-to-map FSC for 8TNQ and 8TNR in EMD-41424 and EMD-41425, respectively. (B, C) Viewing direction distribution for EMD-41424 (B) and EMD-41425 (C). (D, E) Final maps colored according to local resolution. (F, G) 3DFSC (*l*/*l*0) plots and directional resolution histograms for EMD-41424 (F) and EMD-41425 (G). (H) Structural superposition of the models derived from EMD-41424 and

EMD-41425, as well as of DDB1^{ΔBPB}•CRBN•pomalidomide•SD40 (EMD-41423). The range of motion of CRBN in respect to DDB1 is indicated with an arrow. **(I)** Cryo-EM density (golden mesh) for pomalidomide (shown as sticks). **(J)** Cryo-EM density (golden mesh) for PT-179 (shown as sticks). **(K)** Density example with transparent density and SD40 finger colored in magenta. **(L)** Unaccounted density (pink) at the interface between CRBN and DDB1 most likely stems from the uncleaved N-terminal sequence (His₆–TEV) of the DDB1 construct. Sharpened map is shown on the left and a low pass-filtered map from a 3D variability job is shown on the right.

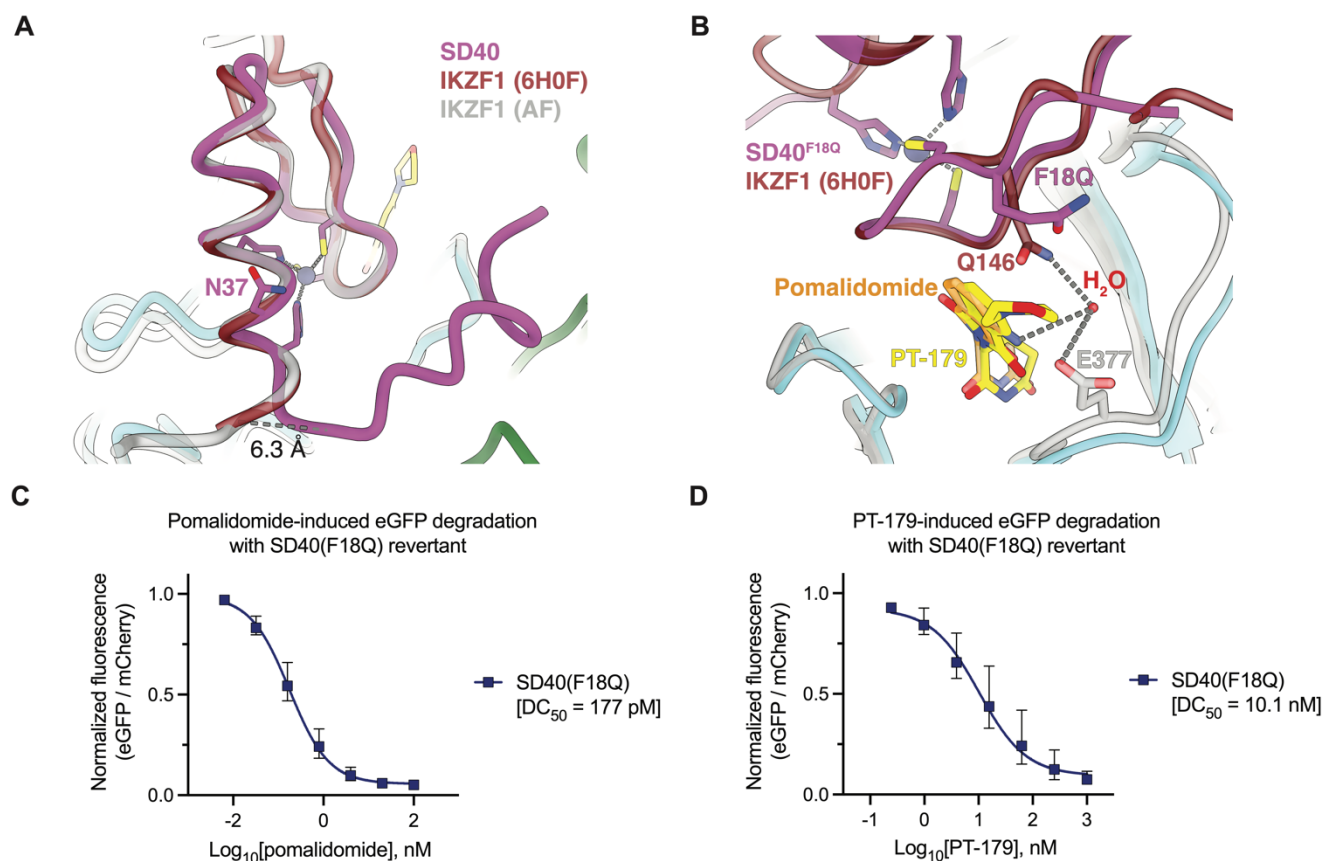

Fig. S20. Comparison of IKZF1, SD40, and SD40^{F18Q}. **(A)** Overlay of SD40 and IKZF1 from both crystal structure and AlphaFold2 model. The SD40 helix terminates two residues before IKZF1 and turns towards CRBN_{NTD} with equivalent residues 6.3 Å apart. **(B)** Rosetta model of SD40^{F18Q} overlaid on IKZF1 from crystal structure. The proposed water-mediated hydrogen bond between pomalidomide, Q146 of IKZF1, and E377 of CRBN is highlighted. The glutamine of SD40^{F18Q} adopts a rotamer that is unable to participate in such a bond but can accommodate PT-179. **(C)** pomalidomide or **(D)** PT-179-induced degradation of eGFP fused to SD40^{F18Q} revertant. Values and error bars in (C,D) represent the mean and range of three or more replicates, ≥20 hrs treatment, all normalized to control treatment with DMSO only.

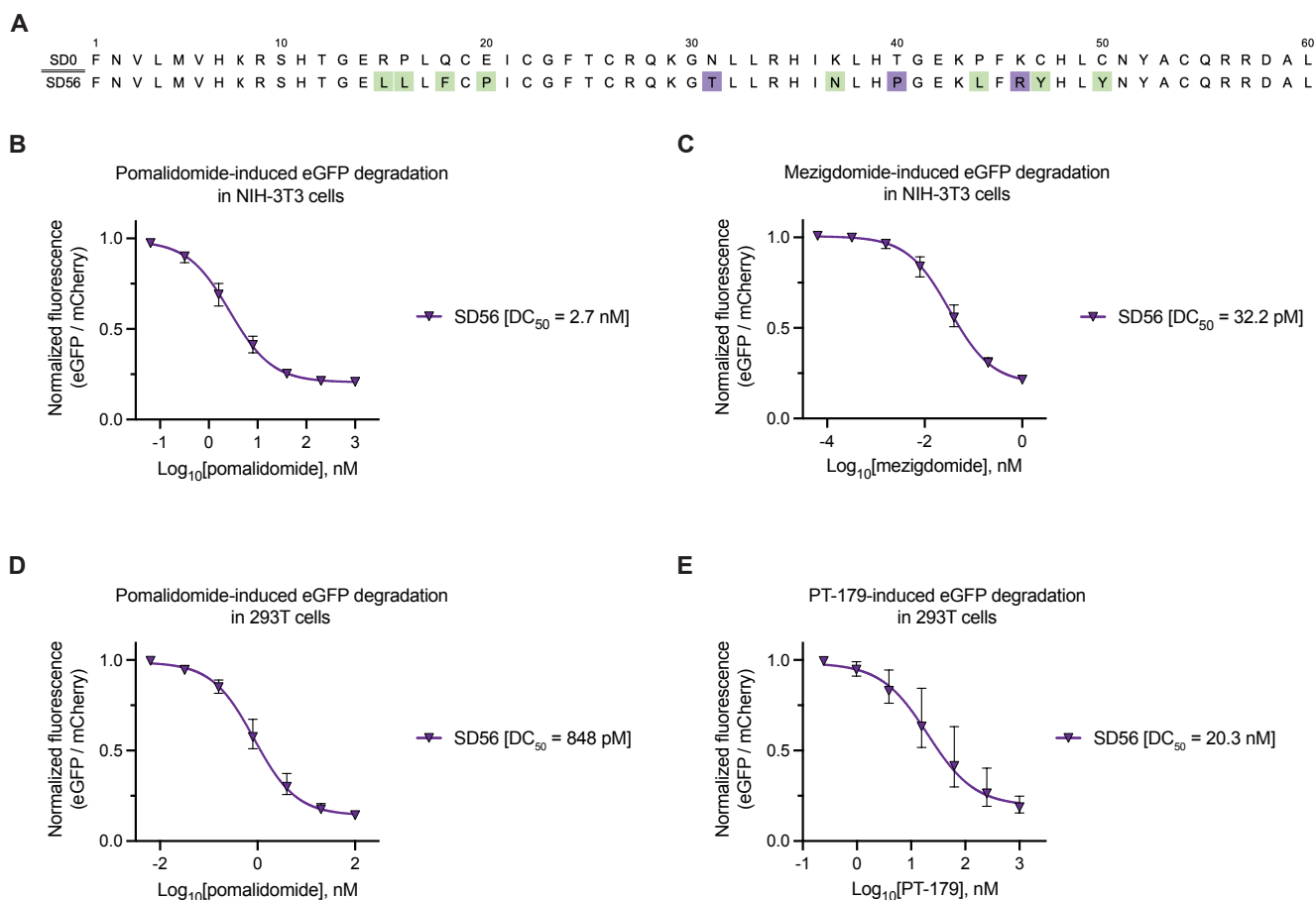

Fig. S21. Mouse CRBN-compatible degrons respond to canonical IMiDs in mouse cells and function in human cells. (A) Complete amino acid sequences of evolved degrons from SD56. Mutations compared to SD0 from evolution experiments with human CRBN (green) or mouse CRBN (purple) are highlighted. (B) pomalidomide or (C) mezigdomide-induced degradation of eGFP tagged with SD56 in mouse NIH-3T3 cells. (D) Pomalidomide or (E) mezigdomide-induced degradation of eGFP tagged with SD56 in HEK293T cells. Values and error bars in (B–E) represent the mean and range of three or more replicates, ≥ 20 hrs treatment, all normalized to control treatment with DMSO only.

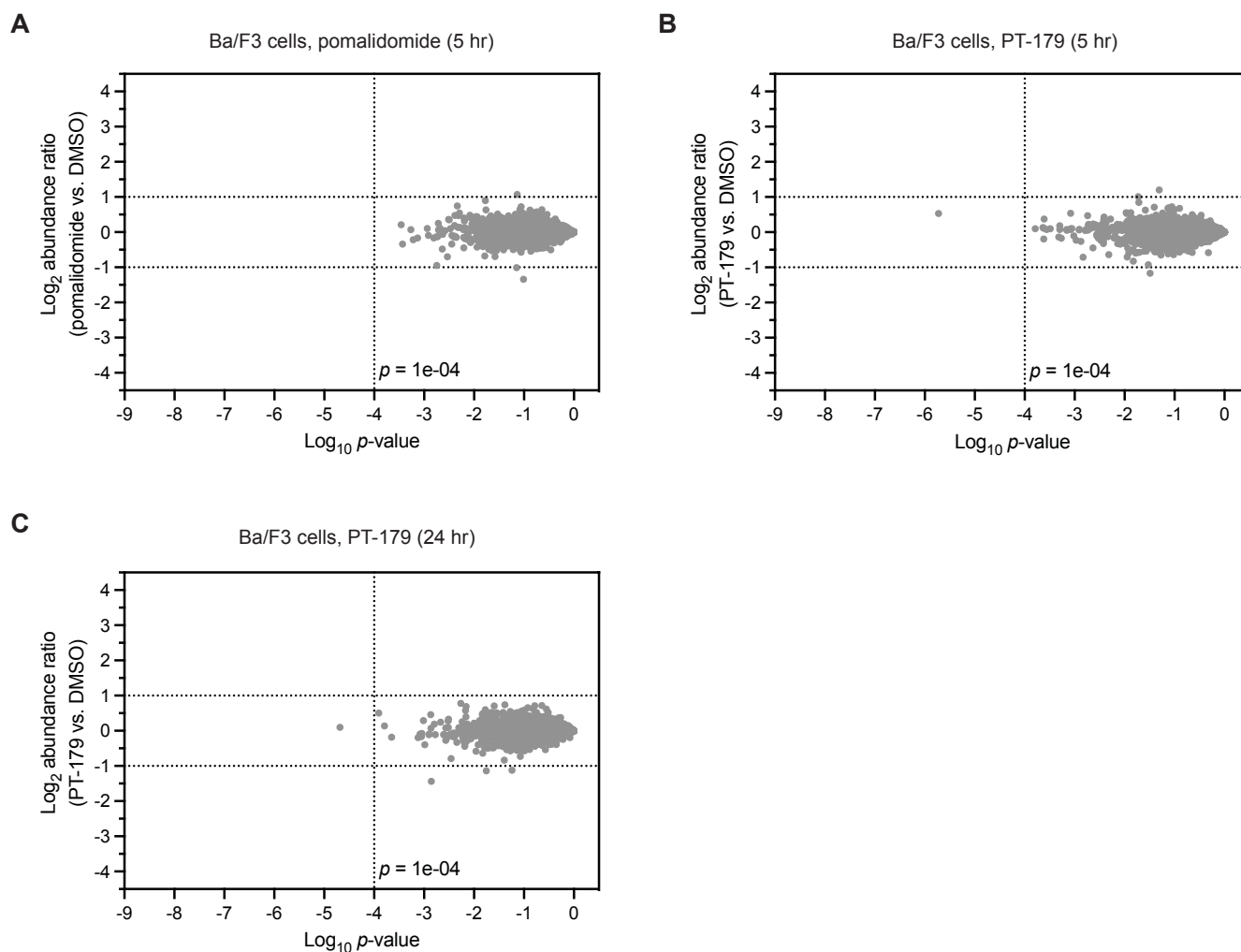

Fig. S22. Pomalidomide and PT-179 do not degrade proteins in mouse Ba/F3 cells. **(A)** Global proteomics by quantitative LCMS analysis of mouse Ba/F3 cells treated with pomalidomide (1 μ M, 5 hrs; 6765 proteins detected), **(B)** PT-179 (1 μ M, 5 hrs; 6792 proteins detected), and **(C)** PT-179 (1 μ M, 24 hrs; 6683 proteins detected) compared to control cells treated with DMSO only.

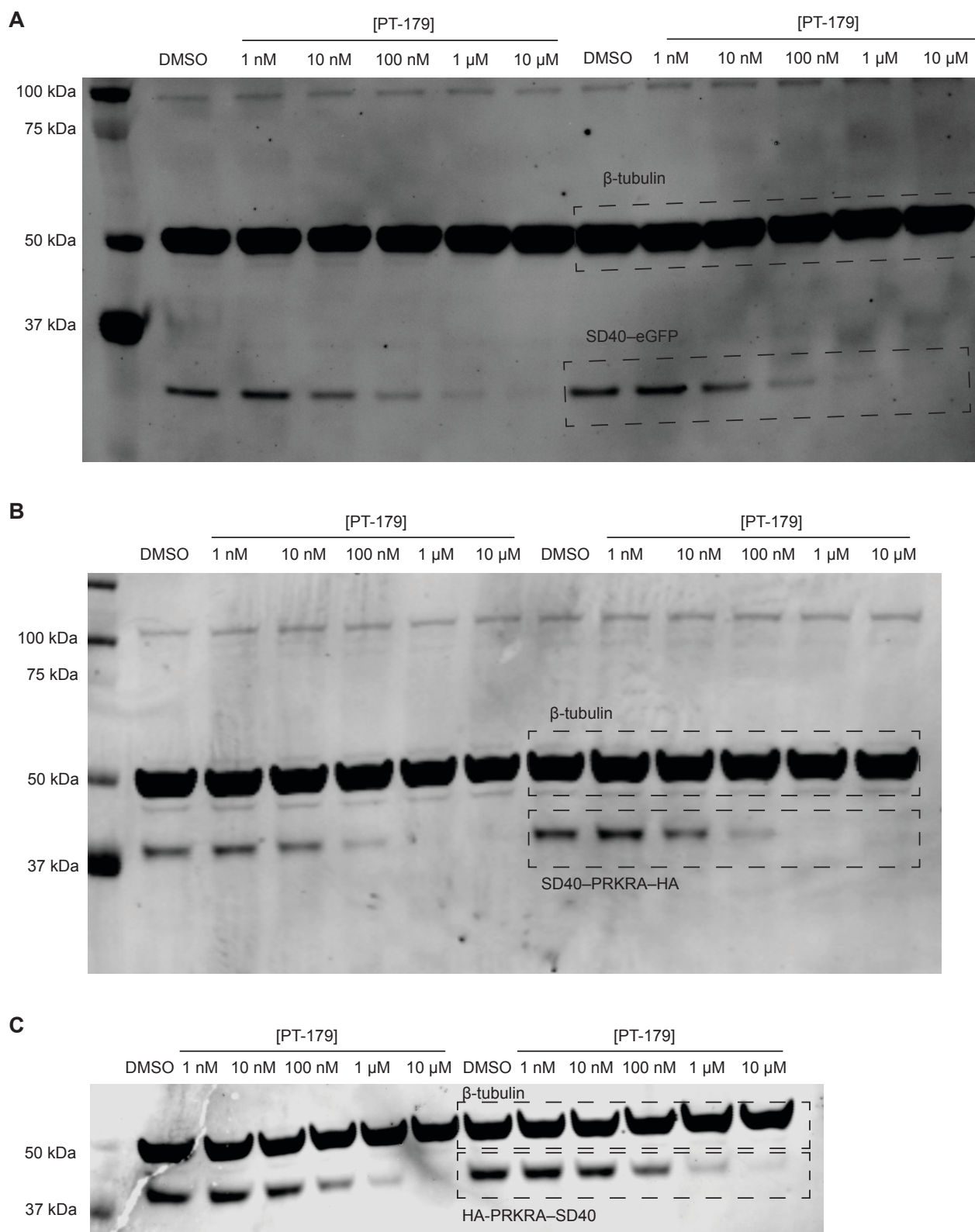

Fig. S23. Uncropped western blots for degradation of exogenously expressed SD40 fusion proteins. (A) Degradation of SD40-eGFP. (B) Degradation of SD40-PRKRA-HA. (C) Degradation of HA-PRKRA-SD40.

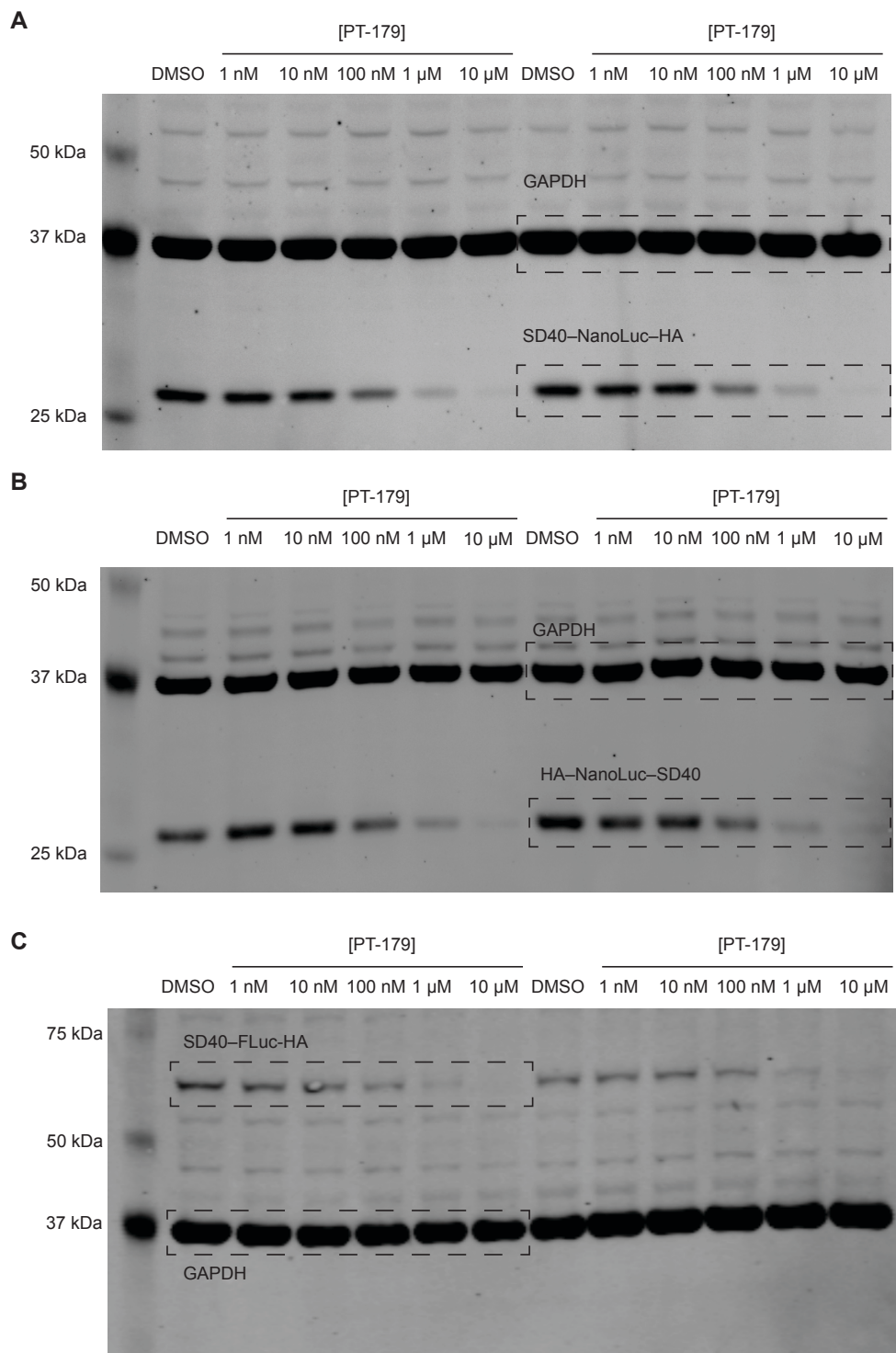

Fig. S24. Uncropped western blots for degradation of exogenously expressed SD40 fusion proteins. (A) Degradation of SD40-NanoLuc-HA. (B) Degradation of HA-NanoLuc-SD40. (C) Degradation of SD40-FLucHA.

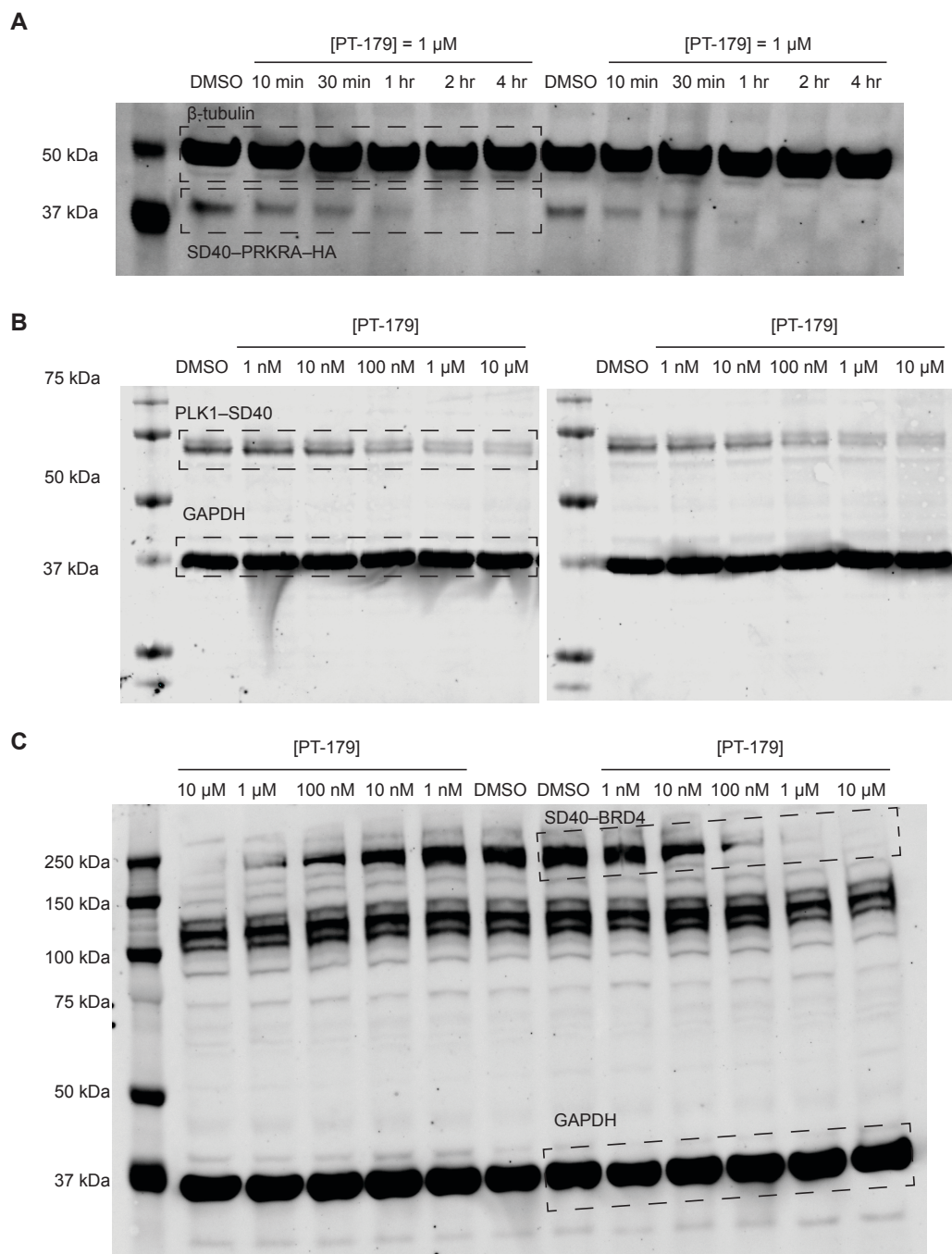

Fig. S25. Uncropped western blots for degradation of SD40 fusion proteins. (A) Time-course for degradation of exogenously expressed SD40-PRKRA-HA. (B) Degradation of endogenously tagged PLK1-SD40 (C) Degradation of endogenously tagged SD40-BRD4.

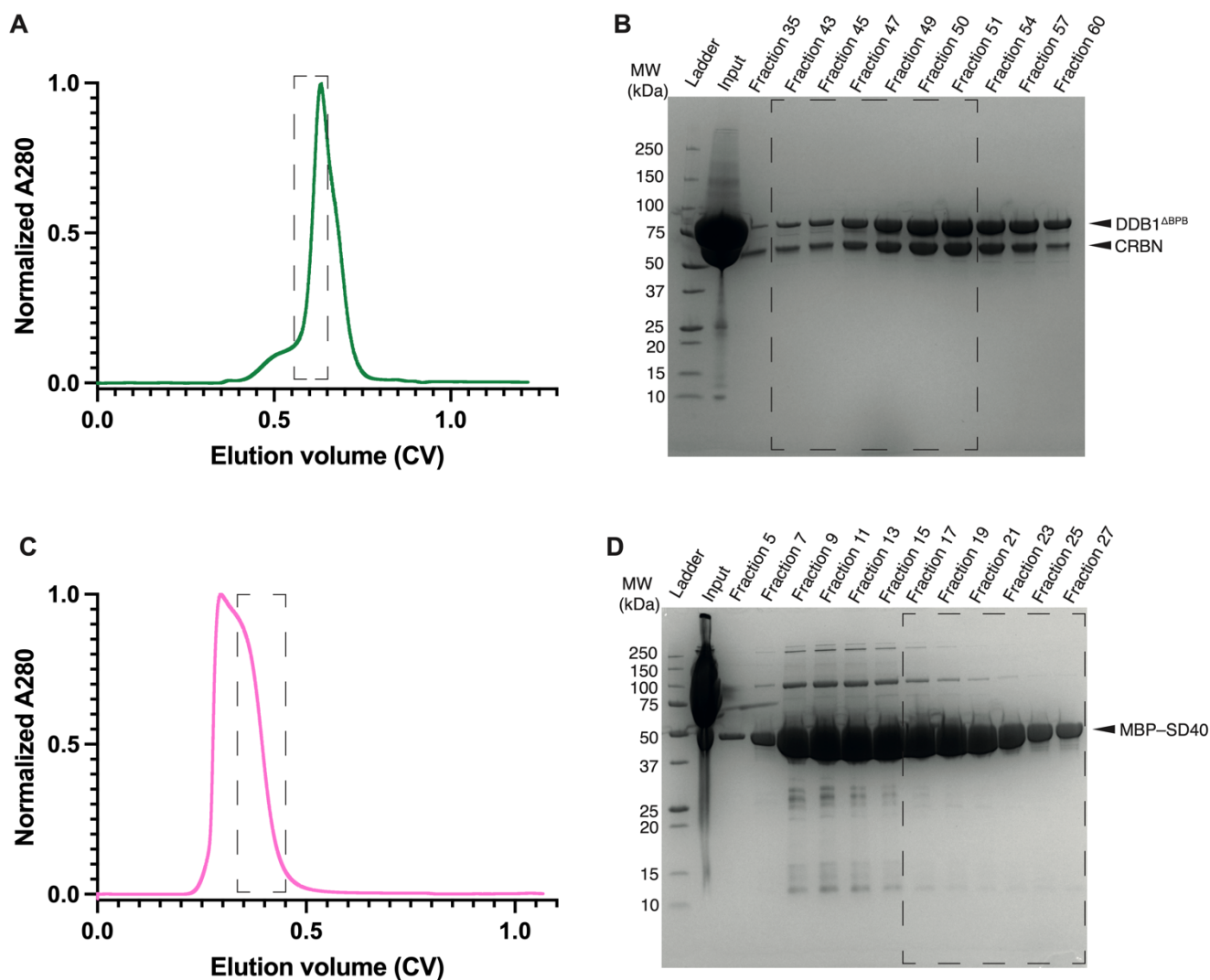

Fig. S26. Purity of samples used for cryo-electron microscopy. (A) Chromatogram from size exclusion chromatography for DDB1 Δ BPB•CRBN. (B) Uncropped SDS gel of size exclusion fractions for DDB1 Δ BPB•CRBN. (C) Chromatogram from size exclusion chromatography for MBP-tagged SD40. (D) Uncropped SDS gel of size exclusion fractions for MBP-tagged SD40. In all panels, the fractions highlighted in the box were collected.

Table S1. Plasmids used in this study.

Name	Class (Resistance)	Origin	ORF1 - Promoter	ORF1 - [RBS] Genes	ORF2 - Promoter	ORF2 - [RBS] Genes	Operator
pJM154	AP (CarbR)	SC101	pro1	[SD8] RR69-Flag-FKBP12	pLac*	[SD8] luxAB	434-OR1
pJM166B	CP (SpecR)	colE1	pro1	[SD8] rpoZ-cMyc-FRB			
pJM194A	AP (CarbR)	SC101	pro1	[SD8] RR69-Flag-cereblonCTD	pLac*	[SD8] luxAB	434-OR1
pJM195A	AP (CarbR)	SC101	pro1	[SD8] RR69-Flag-cereblonCTD	pLac*	[SD8] pIII	434-OR1
pJM195B	AP (CarbR)	SC101	pro1	[SD8] RR69-Flag-cereblonCTD	pLac*	[sd8] pIII	434-OR1
pJM195C	AP (CarbR)	SC101	pro1	[SD8] RR69-Flag-cereblonCTD	pLac*	[sd5] pIII	434-OR1
pJM195G	AP (CarbR)	SC101	pro1	[SD8] RR69-Flag-cereblonCTD	pLac*	[sd2] pIII	434-OR1
pJM185A	AP (CarbR)	SC101	pro1	[SD8] RR69-Flag-CRBN	pLac*	[SD8] luxAB	434-OR1
pJM185D	AP (CarbR)	SC101	pro1	[sd8] RR69-Flag-CRBN	pLac*	[SD8] luxAB	434-OR1
pJM185E	AP (CarbR)	SC101	pro1	[sd5] RR69-Flag-CRBN	pLac*	[SD8] luxAB	434-OR1
pSD033B	AP (CarbR)	SC101	proA	[SD8] RR69-Flag-CRBN	pLac*	[SD8] luxAB	434-OR1
pSD033C	AP (CarbR)	SC101	proB	[SD8] RR69-Flag-CRBN	pLac*	[SD8] luxAB	434-OR1
pJM186A	AP (CarbR)	SC101	pro1	[SD8] RR69-Flag-CRBN	pLac*	[SD8] pIII	434-OR1
pJM186B	AP (CarbR)	SC101	pro1	[SD8] RR69-Flag-CRBN	pLac*	[sd8] pIII	434-OR1
pJM186C	AP (CarbR)	SC101	pro1	[SD8] RR69-Flag-CRBN	pLac*	[sd5] pIII	434-OR1
pSD047A	AP (CarbR)	SC101	pro1	[SD8] RR69-Flag-CRBN	pLac*	[sd2] pIII	434-OR1
pSD025A	AP (KanR)	p15A	pro1	[SD8] scP22-Flag-CRBN(W384A, Y386A)	pLac*	[SD8] pIII-neg	P22-OL1
pSD025B	AP (CarbR)	p15A	pro1	[SD8] scP22-Flag-CRBN(W384A, Y386A)	pLac*	[sd8] pIII-neg	P22-OL1
pSD025C	AP (CarbR)	p15A	pro1	[SD8] scP22-Flag-CRBN(W384A, Y386A)	pLac*	[sd5] pIII-neg	P22-OL1
pSD025D	AP (CarbR)	p15A	pro1	[SD8] scP22-Flag-CRBN(W384A, Y386A)	pLac*	[sd2] pIII-neg	P22-OL1
pSD139A	AP (CarbR)	SC101	pro1	[SD8] scP22-Flag-CRBN(W384A, Y386A)	pLac*	[SD8] luxAB	P22-OL1
pSD137A	AP (CarbR)	SC101	pro1	[SD8] scP22-Flag-CRBN	pLac*	[SD8] luxAB	434-OR1
pSD138A	AP (CarbR)	SC101	pro1	[SD8] scP22-Flag-CRBN	pLac*	[SD8] luxAB	P22-OL1
pSD140A	AP (CarbR)	SC101	pro1	[SD8] RR69-Flag-CRBN	pLac*	[SD8] luxAB	P22-OL1
pSD094B	AP (CarbR)	SC101	pro1	[SD8] RR69-Flag-mmCRBN	pLac*	[sd5] pIII	434-OR1
pSD090A	AP (CarbR)	SC101	pro1	[SD8] RR69-Flag-mmCRBN	pLac*	[SD8] luxAB	434-OR1
pSD097D	CP (SpecR)	colE1	pro1	[SD8] rpoZ-cMyc-SD55			
pSD113A	CP (SpecR)	colE1	pro1	[SD8] rpoZ-cMyc-SD56			
pSD103D	CompP (SpecR)	colE1	proD	[SD8] MBP-SD55			
pJM189A	CP (SpecR)	colE1	pro1	[SD8] rpoZ-cMyc-SD0			
pJM332A	CP (SpecR)	colE1	proA	[SD8] rpoZ-cMyc-SD0			
pJM332B	CP (SpecR)	colE1	proB	[SD8] rpoZ-cMyc-SD0			
pJM332C	CP (SpecR)	colE1	pro1	[sd8] rpoZ-cMyc-SD0			
pJM332D	CP (SpecR)	colE1	pro1	[sd5] rpoZ-cMyc-SD0			
pJM189E	CP (SpecR)	colE1	pro1	[SD8] rpoZ-cMyc-SD8			
pJM189J	CP (SpecR)	colE1	pro1	[SD8] rpoZ-cMyc-SD12			
pSD012E	CP (SpecR)	colE1	pro1	[SD8] rpoZ-cMyc-SD17			
pSD014C	CP (SpecR)	colE1	pro1	[SD8] rpoZ-cMyc-SD20			

pSD275G	CP (SpecR)	colE1	pro1	[SD8] rpoZ-cMyc-SD20.1			
pSD275I	CP (SpecR)	colE1	pro1	[SD8] rpoZ-cMyc-SD20.2			
pSD034F	CP (SpecR)	colE1	pro1	[SD8] rpoZ-cMyc-SD31			
pSD046B	CP (SpecR)	colE1	pro1	[SD8] rpoZ-cMyc-SD35			
pSD057C	CP (SpecR)	colE1	pro1	[SD8] rpoZ-cMyc-SD36			
pSD069A	Lenti (CarbR)	pBR322	hPGK	SD0-eGFP-IRES-mCherry			
pSD069B	Lenti (CarbR)	pBR322	hPGK	SD8-eGFP-IRES-mCherry			
pSD121B	Lenti (CarbR)	pBR322	hPGK	SD20-eGFP-IRES-mCherry			
pSD082A	Lenti (CarbR)	pBR322	hPGK	SD31-eGFP-IRES-mCherry			
pSD068F	Lenti (CarbR)	pBR322	hPGK	SD36-eGFP-IRES-mCherry			
pSD080A	Lenti (CarbR)	pBR322	hPGK	SD40-eGFP-IRES-mCherry			
pSD225A	Lenti (CarbR)	pBR322	hPGK	SD40(F18Q)-eGFP-IRES-mCherry			
pSD080B	Lenti (CarbR)	pBR322	hPGK	SD41-eGFP-IRES-mCherry			
pSD080C	Lenti (CarbR)	pBR322	hPGK	SD42-eGFP-IRES-mCherry			
pSD141B	Lenti (CarbR)	pBR322	hPGK	SD56-eGFP-IRES-mCherry			
pSD141D	Lenti (CarbR)	pBR322	hPGK	SD63-eGFP-IRES-mCherry			
pSD092A	Lenti (CarbR)	pBR322	hPGK	SD40-PRKRA-HA-IRES-mCherry			
pSD092C	Lenti (CarbR)	pBR322	hPGK	HA-PRKRA-SD40-IRES-mCherry			
pSD115A	Lenti (CarbR)	pBR322	hPGK	SD40-NanoLuc-HA-IRES-mCherry			
pSD115B	Lenti (CarbR)	pBR322	hPGK	HA-NanoLuc-SD40-IRES-mCherry			
pSD115C	Lenti (CarbR)	pBR322	hPGK	SD40-Fluc-HA-IRES-mCherry			
pSD118A	Expression (KanR)	pBR322	T7	AviTag-6xHis-MBP-TEV-SD0(ZF2-3)			
pSD117A	Expression (KanR)	pBR322	T7	AviTag-6xHis-MBP-TEV-SD40			
pSSRB069	Baculo (AmpR)	pAC8	polyhedrin	6xHis-TEV-hsDDB1dB	p10	dsRed2	
pSSRB070	Baculo (AmpR)	pAC8	polyhedrin	FLAG-TEV-Spy-hsCRBN	p10	dsRed2	
pSSRB262	Expression (KanR)	pBR322	T7	AviTag-6xHis-MBP-Prescission-FLAG-SD40			
pSSRB271	Expression (KanR)	pBR322	T7	10xHis-TEV-SpyCatcher_22-104_S50C			
pSSRB274	Baculo (AmpR)	pAC8	polyhedrin	FLAG-TEV-eGFP-Prescission-hsCRBN	p10	dsRed2	

Table S2. DNA sequences of degtron constructs for phage-assisted evolution, protein purification, and HDR.

Promoter-[RBS]-RpoZ-cMyc-SD0

AATTCACCTCGAAAGCAAGTTGATAAACTGATACAATTAAAGGCTCCTTTTGGAGCCTTTT
TTTTTGGAGTAAGGAGGAAAAAATGGCACGCGTAACTGTTTCAGGACGCTGTAGAGAAA
ATTGGTAACCGTTTTGACCTGGTACTGGTCGCCGCGCGTCGCGTTCGTCAGCTACAGGTAG
GCGGAAAGGATCCACTGGTACCGGAAAAAACGATAAAACCACTGTAATCGCGCTGCGC
GAAATCGAAGAAGGTCTGATCAACAACCAGTTCCTCGACGTTTCGCGAACGCCAGGAACA
GCAAGAGCAGGAAGTCGCTGAATTACAAGCCGTTACCGCTATTGCTGAAGGTTCGTCGTGC
GGCCGCGAACAAGCTTATTTCTGAAGAGGACTTG
TTCAATGTACTGATGGTCCATAAACGGAGTCACACTGGCGAGCGCCCGCTCCAATGTGAA
ATCTGCGGGTTACGTGTCGGCAGAAGGGCAACCTCCTCCGGCATATCAAGCTGCACACG
GGTGAAAAACCGTTTAAGTGCCATCTCTGCAATTACGCCTGTCAGAGAAGAGATGCTTTG
TAA

RED = Promoter; Green = [RBS]; Orange = RpoZ; Purple = cMyc; Blue = SD0

pT7-lac-[RBS]-AviTag-6xHis-MBP-TEV-SD0(ZF2-3)

TAATACGACTCACTATAGGGGGAATTGTGAGCGGATAACAATTCCCCTCTAGAAATAATT
TTGTTTAACTTTAAGAAGGAGATATACATATGGGCTTAAACGACATTTTTTGAGGCTCAA
AGATCGAATGGCACGAGGGCAGCTCTCATCATCATCACCGGTTCCAGTAAGATCG
AAGAAGGCAAGTTAGTGATTTGGATCAACGGCGATAAAGGCTATAACGGTTTAGCGGAA
GTAGGCAAGAAGTTTGAGAAGGATACCGGAATCAAAGTGACGGTCGAACACCCGGATAA
GTTAGAGGAGAAGTTCCCTCAAGTAGCGGCAACGGGCGACGGTCCTGACATCATCTTTTG
GGCACACGACCGCTTCGGTGGTTACGCTCAGTCAGGTTTATTAGCGGAGATTACTCCAGA
CAAAGCGTTCCAGGACAAGTTATATCCATTTACGTGGGACGCAGTGCGTTATAACGGCAA
GTTAATCGCATATCCCATTTGCGGTAGAAGCCCTGAGCCTGATTTACAACAAGGACCTGTT
ACCCAACCCTCCGAAGACGTGGGAGGAGATTCCGGCTTTAGATAAGGAACTGAAAGCGA
AAGGCAAGTCTGCCCTGATGTTTAACCTGCAAGAACCCTACTTCACCTGGCCTCTGATCGC
GGCGGATGGCGGTTATGCGTTTAAGTACGAAAACGGCAAATACGATATTAAGGATGTAGG
CGTCGATAACGCCGGTGCCAAAGCCGGTCTGACCTTTTTAGTTGATCTGATTAAGAATAA
GCACATGAACGCAGACACCGACTACAGCATTGCGGAAGCTGCGTTTAATAAAGGTGAGA
CCGCCATGACGATTAACGGACCTTGGGCGTGGTCGAACATTGATACCAGTAAAGTCAATT
ACGGCGTTACAGTCCTGCCGACCTTCAAAGGTCAGCCGTCAAACCGTTTCGTAGGTGTCTT
ATCCGCCGGTATCAACGCGGCGTCCCCAAATAAAGAGTTGGCTAAAGAGTTCCTGGAAAA
CTATCTGCTGACAGACGAAGGACTGGAGGCTGTGAACAAGGACAAGCCACTGGGCGCTG
TTGCGCTGAAAAGTTATGAGGAAGAACTGGCGAAAGATCCCCGCATCGCGGCGACGATG
GAAAACGCCCAAAAAGGCGAAATCATGCCGAACATTCGCGCAGATGTCAGCTTTTTTGGTAT
GCCGTACGCACGGCTGTTATTAACGCCGCGTCGGGCCGCCAAACCGTTGATGAGGCACTG
AAGGACGCGCAGACTCGTATCACCAGGGTAGCGAAAATCTTTATTTTCAAGGCTCGGGT
GGAGGGGGCTCGGGAGGTGGCGGTGGGGAACGTCCCCTTCAATGTGAAATTTGCGGCTTT
ACGTGTCGTCAGAAGGGAAATTTGTTACGCCATATTAAACTGCATACGGGTGAGAAACCG
TTTAAGTGTCACCTGTGTAATTACGCATGTCAACGTCGTGACGCTCTGACTGGACACTTAC
GCACGCACTCCGTAATCAAGGAGGAGTGA

RED = pT7; Gray = lac; Green = [RBS]; Orange = AviTag; Purple = 6xHis; Blue = MBP; Peach = TEV; Brown = SD0(ZF2-3)

pT7-lac-[RBS]-AviTag-6xHis-MBP-TEV-SD40

TAATACGACTCACTATAGGGGGAATTGTGAGCGGATAACAATTCCCCTCTAGAAATAATT
TTGTTTAACTTTAAGAAGGAGATATACATATGGGCTTAAACGACATTTTTGAGGCTCAAA
AGATCGAATGGCACGAGGGCAGCTCTCATCATCATCACACCGTTCCAGTAAGATCG
AAGAAGGCAAGTTAGTGATTTGGATCAACGGCGATAAAGGCTATAACGGTTTAGCGGAA
GTAGGCAAGAAGTTTGAGAAGGATACCGGAATCAAAGTGACGGTCGAACACCCGGATAA
GTTAGAGGAGAAGTTCCCTCAAGTAGCGGCAACGGGCGACGGTCCTGACATCATCTTTTG
GGCACACGACCGCTTCGGTGGTTACGCTCAGTCAGGTTTATTAGCGGAGATTACTCCAGA
CAAAGCGTTCCAGGACAAGTTATATCCATTTACGTGGGACGCAGTGCGTTATAACGGCAA
GTTAATCGCATATCCCATTTGCGGTAGAAGCCCTGAGCCTGATTTACAACAAGGACCTGTT
ACCCAACCCTCCGAAGACGTGGGAGGAGATTCCGGCTTTAGATAAGGAACTGAAAGCGA
AAGGCAAGTCTGCCCTGATGTTTAACTGCAAGAACCCTACTTCACCTGGCCTCTGATCGC
GGCGGATGGCGGTTATGCGTTTAAGTACGAAAACGGCAAATACGATATTAAGGATGTAGG
CGTCGATAACGCCGGTGCCAAAGCCGGTCTGACCTTTTTAGTTGATCTGATTAAGAATAA
GCACATGAACGCAGACACCGACTACAGCATTGCGGAAGCTGCGTTTAAATAAAGGTGAGA
CCGCCATGACGATTAACGGACCTTGGGCGTGGTTCGAACATTGATACCAGTAAAGTCAATT
ACGGCGTTACAGTCCTGCCGACCTTCAAAGGTCAGCCGTCAAACCGTTCGTAGGTGTCTT
ATCCGCCGGTATCAACGCGGCGTCCCCAAATAAAGAGTTGGCTAAAGAGTTCCTGGAAAA
CTATCTGCTGACAGACGAAGGACTGGAGGCTGTGAACAAGGACAAGCCACTGGGCGCTG
TTGCGCTGAAAAGTTATGAGGAAGAACTGGCGAAAGATCCCCGCATCGCGGCGACGATG
GAAAACGCCCAAAAAGGCGAAATCATGCCGAACATTCCGCAGATGTCAGCTTTTTTGGTAT
GCCGTACGCACGGCTGTTATTAACGCCGCGTCGGGCCGCCAAACCGTTGATGAGGCACTG
AAGGACGCGCAGACTCGTATCACCAAGGGTAGCGAAAATCTTTATTTTCAAGGCTCGGGT
GGAGGGGGCTCGGGAGGTGGCGGTCTGTTGCTGTTCTGCCCTATTTGCGGGTTTACATGTC
GCCAGAAGGGCAACTTACTTCGCCATATTAACCTGCACACAGGGGAAAAGTTATTTAAGT
ACCACCTGTATTGA

RED = pT7; Gray = lac; Green = [RBS]; Orange = AviTag; Purple = 6xHis; Blue = MBP; Peach = TEV; Brown = SD40

pT7-lac-[RBS]-AviTag-6xHis-MBP-PreScission-FLAG-SD40

TAATACGACTCACTATAGGGGGAATTGTGAGCGGATAACAATTCCCCTCTAGAAATAATT
TTGTTTAACTTTAAGAAGGAGATATACATATGGGCTTAAACGACATTTTTGAGGCTCAAA
AGATCGAATGGCACGAGGGCAGCTCTCATCATCATCACACCGTTCCAGTAAGATCG
AAGAAGGCAAGTTAGTGATTTGGATCAACGGCGATAAAGGCTATAACGGTTTAGCGGAA
GTAGGCAAGAAGTTTGAGAAGGATACCGGAATCAAAGTGACGGTCGAACACCCGGATAA
GTTAGAGGAGAAGTTCCCTCAAGTAGCGGCAACGGGCGACGGTCCTGACATCATCTTTTG
GGCACACGACCGCTTCGGTGGTTACGCTCAGTCAGGTTTATTAGCGGAGATTACTCCAGA
CAAAGCGTTCCAGGACAAGTTATATCCATTTACGTGGGACGCAGTGCGTTATAACGGCAA
GTTAATCGCATATCCCATTTGCGGTAGAAGCCCTGAGCCTGATTTACAACAAGGACCTGTT
ACCCAACCCTCCGAAGACGTGGGAGGAGATTCCGGCTTTAGATAAGGAACTGAAAGCGA
AAGGCAAGTCTGCCCTGATGTTTAACTGCAAGAACCCTACTTCACCTGGCCTCTGATCGC

GGCGGATGGCGGTTATGCGTTTAAGTACGAAAACGGCAAATACGATATTAAGGATGTAGG
 CGTCGATAACGCCGGTGCCAAAGCCGGTCTGACCTTTTTAGTTGATCTGATTAAGAATAA
 GCACATGAACGCAGACACCGACTACAGCATTGCGGAAGCTGCGTTTAATAAAGGTGAGA
 CCGCCATGACGATTAACGGACCTTGGGCGTGGTTCGAACATTGATACCAGTAAAGTCAATT
 ACGGCGTTACAGTCCTGCCGACCTTCAAAGGTCAGCCGTCAAACCGTTTCGTAGGTGTCTT
 ATCCGCCGGTATCAACGCGGGCTCCCCAAATAAAGAGTTGGCTAAAGAGTTCCTGGAAAA
 CTATCTGCTGACAGACGAAGGACTGGAGGCTGTGAACAAGGACAAGCCACTGGGCGCTG
 TTGCGCTGAAAAGTTATGAGGAAGAACTGGCGAAAGATCCCCGCATCGCGGCGACGATG
 GAAAACGCCCAAAAAGGCGAAATCATGCCGAACATTCCGCAGATGTCAGCTTTTTGGTAT
 GCCGTACGCACGGCTGTTATTAACGCCGCGTCGGGGCCGCCAAACCGTTGATGAGGCACTG
 AAGGACGCGCAGACTCGTATCACCAAGCTGGAAGTTCTGTTCCAGGGGCCCCGACTACAAG
 GACGACGATGACAAGTCGGGAGGTGGCGGTCTGTTGCTGTTCTGCCCTATTTGCGGGTTTA
 CATGTCGCCAGAAGGGCAACTTACTTCGCCATATTAACCTGCACACAGGGGAAAAGTTAT
 TTAAGTACCACCTGTATTGA

RED = pT7; Gray = lac; Green = [RBS]; Orange = AviTag; Purple = 6xHis; Blue = MBP; Peach =
 PreScission; Magenta = FLAG; Brown = SD40

HDR Donr gene block for SD40

CTTCTCCTGTTTTGCCCCATATGCGGCTTCACTTGTTCGGCAAAGGGTAACCTGCTGCGAC
 ACATCAACCTGCATACTGGTGAGAAGCTGTTCAAATATCACCTGTAT

HDR Donr gene block for FKBP12

GGCGTCCAGGTTCGAGACCATTTGCCCCGGGGATGGGCGTACATTCCCGAAACGTGGGCAG
 ACGTGCGTGGTACATTACACAGGGATGTTAGAAGACGGTAAAAAAGTTGATTCTAGCCGC
 GACCGCAATAAGCCTTTCAAATTTATGCTGGGCAAACAGGAGGTCATCCGTGGTTGGGAA
 GAAGGCGTAGCCCAAATGTCGGTTGGACAGCGTGCAAATTAACCTATTTCTCCTGACTAC
 GCATACGGTGCCACGGGGCATCCAGGAATTATCCCCCCTCACGCGACGCTGGTGTTCGAC
 GTTGAGTTACTTAAATTGGAG

Table S3. Sequences of prime editing pegRNAs and sgRNAs. All pegRNAs share the scaffold sequence GTTTAAGAGCTATGCTGGAAACAGCATAGCAAGTTTAAATAAGGCTAGTCCGTTATCAACTTGAAAAAGTGGCACCGAGTCGGTGC (114) between the spacer and 3' extension and terminate with the evopreQ1 motif CGCGTTTCTATCTAGTTACGCGTTAAACCAACTAGAA (115) after the 3' extension. Nicking guide DTN487 shares the same scaffold sequence and does not have a 3' extension.

Name	Spacer	3' Extension (RTT and PBS)
PLK1 screen 1		
277A	GCAAGCTGCTGAGCTCACGCT	GGAGATTCCCTTTCTGCCTACAGGTGAATCCGCAGAT<br>GGGGCAAAAGAGCAGGAGAGAAGCTTTAAGGCGATTA<br>CTAGCTGAACGTGAGCTCAGCAGCT
277B	GCAAGCTGCTGAGCTCACGCT	GGAGATTCCCTTTCTGCCTACAGGTGAATCCGCAGAT<br>GGGGCAAAAGAGCAGGAGAGAAGCTTTAAGGCGATTA<br>CTAGCTGAACGTGAGCTCAGCAG
277C	GCAAGCTGCTGAGCTCACGCT	GGAGATTCCCTTTCTGCCTACAGGTGAATCCGCAGAT<br>GGGGCAAAAGAGCAGGAGAGAAGCTTTAAGGCGATTA<br>CTAGCTGAACGTGAGCTCAGC
277D	GCAAGCTGCTGAGCTCACGCT	GGAGATTCCCTTTCTGCCTACAGGTGAATCCGCAGAT<br>GGGGCAAAAGAGCAGGAGAGAAGCTTTAAGGCGATTA<br>CTAGCTGAACGTGAGCTCA
277E	GCAAGCTGCTGAGCTCACGCT	TTTCTGCCTACAGGTGAATCCGCAGATGGGGCAAAAG<br>AGCAGGAGAGAAGCTTTAAGGCGATTACTAGCTGAAC<br>GTGAGCTCAGCAGCT
277F	GCAAGCTGCTGAGCTCACGCT	TTTCTGCCTACAGGTGAATCCGCAGATGGGGCAAAAG<br>AGCAGGAGAGAAGCTTTAAGGCGATTACTAGCTGAAC<br>GTGAGCTCAGCAG
277G	GCAAGCTGCTGAGCTCACGCT	TTTCTGCCTACAGGTGAATCCGCAGATGGGGCAAAAG<br>AGCAGGAGAGAAGCTTTAAGGCGATTACTAGCTGAAC<br>GTGAGCTCAGC
277H	GCAAGCTGCTGAGCTCACGCT	TTTCTGCCTACAGGTGAATCCGCAGATGGGGCAAAAG<br>AGCAGGAGAGAAGCTTTAAGGCGATTACTAGCTGAAC<br>GTGAGCTCA
278A	GACACTGCAGACATGGCACCG	GATTCACCTGTAGGCAGAAAGGGAATCTCCTTCGGCA<br>CATCAACTTGCATACAGGTGAGAAATTGTTCAAGTATCA<br>TCTGTACTAATGCCATGTCTGCAGT
278B	GACACTGCAGACATGGCACCG	GATTCACCTGTAGGCAGAAAGGGAATCTCCTTCGGCA<br>CATCAACTTGCATACAGGTGAGAAATTGTTCAAGTATCA<br>TCTGTACTAATGCCATGTCTGCA
278C	GACACTGCAGACATGGCACCG	GATTCACCTGTAGGCAGAAAGGGAATCTCCTTCGGCA<br>CATCAACTTGCATACAGGTGAGAAATTGTTCAAGTATCA<br>TCTGTACTAATGCCATGTCTG
278D	GACACTGCAGACATGGCACCG	GATTCACCTGTAGGCAGAAAGGGAATCTCCTTCGGCA<br>CATCAACTTGCATACAGGTGAGAAATTGTTCAAGTATCA<br>TCTGTACTAATGCCATGTC
278E	GACACTGCAGACATGGCACCG	TAGGCAGAAAGGGAATCTCCTTCGGCACATCAACTTG<br>CATACAGGTGAGAAATTGTTCAAGTATCATCTGTACTAA<br>TGCCATGTCTGCAGT
278F	GACACTGCAGACATGGCACCG	TAGGCAGAAAGGGAATCTCCTTCGGCACATCAACTTG<br>CATACAGGTGAGAAATTGTTCAAGTATCATCTGTACTAA<br>TGCCATGTCTGCA
278G	GACACTGCAGACATGGCACCG	TAGGCAGAAAGGGAATCTCCTTCGGCACATCAACTTG<br>CATACAGGTGAGAAATTGTTCAAGTATCATCTGTACTAA<br>TGCCATGTCTG
278H	GACACTGCAGACATGGCACCG	TAGGCAGAAAGGGAATCTCCTTCGGCACATCAACTTG<br>CATACAGGTGAGAAATTGTTCAAGTATCATCTGTACTAA<br>TGCCATGTC
PLK1 screen 2		

313A	CAAGCTGCTGAGCTCACGCT	TTTCTGCCTACAGGTGAATCCGCAGATGGGGCAAAG<br>AGCAGGAGAGAAGCTTTAAGGCGATTACTAGCTGAAC<br>GTGAGCTCAGCAG
313B	CAAGCTGCTGAGCTCACGCT	CCCTTTCTGCCTACAGGTGAATCCGCAGATGGGGCAA<br>AAGAGCAGGAGAGAAGCTTTAAGGCGATTACTAGCTG<br>AACGTGAGCTCAGCAG
313C	CAAGCTGCTGAGCTCACGCT	GATTCCTTTCTGCCTACAGGTGAATCCGCAGATGGG<br>GCAAAAGAGCAGGAGAGAAGCTTTAAGGCGATTACTA<br>GCTGAACGTGAGCTCAGCAG
314A	ACACTGCAGACATGGCACCG	GATTCACCTGTAGGCAGAAAGGGAATCTCCTTCGGCA<br>CATCAACTTGCATACAGGTGAGAAATTGTTCAAGTATCA<br>TCTGTACTAATGCCATGTCTG
314B	ACACTGCAGACATGGCACCG	TCACCTGTAGGCAGAAAGGGAATCTCCTTCGGCACAT<br>CAACTTGCATACAGGTGAGAAATTGTTCAAGTATCATCT<br>GTACTAATGCCATGTCTG
314C	ACACTGCAGACATGGCACCG	CCTGTAGGCAGAAAGGGAATCTCCTTCGGCACATCAA<br>CTTGCATACAGGTGAGAAATTGTTCAAGTATCATCTGTA<br>CTAATGCCATGTCTG
BRD4 screen 1		
321A	TGCCTGGTGAAGAATGTGAT	TCTCCTGTATGGAGGTTGATGTGCCTGAGAAGATTGCC<br>TTTCTGCCGACAAGTAAAACCGCAGATCGGGCAGAAC<br>AACAGCAACATGCTAGTGATCCCATCACATTCTTCACC<br>A
321B	TGCCTGGTGAAGAATGTGAT	TCTCCTGTATGGAGGTTGATGTGCCTGAGAAGATTGCC<br>TTTCTGCCGACAAGTAAAACCGCAGATCGGGCAGAAC<br>AACAGCAACATGCTAGTGATCCCATCACATTCTTCA
321C	TGCCTGGTGAAGAATGTGAT	TGTATGGAGGTTGATGTGCCTGAGAAGATTGCCTTTCT<br>GCCGACAAGTAAAACCGCAGATCGGGCAGAACAAACA<br>GCAACATGCTAGTGATCCCATCACATTCTTCACCA
321D	TGCCTGGTGAAGAATGTGAT	TGTATGGAGGTTGATGTGCCTGAGAAGATTGCCTTTCT<br>GCCGACAAGTAAAACCGCAGATCGGGCAGAACAAACA<br>GCAACATGCTAGTGATCCCATCACATTCTTCA
322A	CTCTTCCTTTGTAGAGTGCC	TCTCCTGTATGGAGGTTGATGTGCCTGAGAAGATTGCC<br>TTTCTGCCGACAAGTAAAACCGCAGATCGGGCAGAAC<br>AACAGCAACATGCTAGTGATCCCATCACATTCTTCACC<br>AGGCACTCTACAAAGGA
322B	CTCTTCCTTTGTAGAGTGCC	TCTCCTGTATGGAGGTTGATGTGCCTGAGAAGATTGCC<br>TTTCTGCCGACAAGTAAAACCGCAGATCGGGCAGAAC<br>AACAGCAACATGCTAGTGATCCCATCACATTCTTCACC<br>AGGCACTCTACAAA
322C	CTCTTCCTTTGTAGAGTGCC	TGTATGGAGGTTGATGTGCCTGAGAAGATTGCCTTTCT<br>GCCGACAAGTAAAACCGCAGATCGGGCAGAACAAACA<br>GCAACATGCTAGTGATCCCATCACATTCTTCACCAGGC<br>ACTCTACAAAGGA
322D	CTCTTCCTTTGTAGAGTGCC	TGTATGGAGGTTGATGTGCCTGAGAAGATTGCCTTTCT<br>GCCGACAAGTAAAACCGCAGATCGGGCAGAACAAACA<br>GCAACATGCTAGTGATCCCATCACATTCTTCACCAGGC<br>ACTCTACAAA
323A	TGGGATCACTAGCATGTCTG	TCTCCTGTATGGAGGTTGATGTGCCTGAGAAGATTGCC<br>TTTCTGCCGACAAGTAAAACCGCAGATCGGGCAGAAC<br>AACAGCAACATGCTAGTGAT
323B	TGGGATCACTAGCATGTCTG	TCTCCTGTATGGAGGTTGATGTGCCTGAGAAGATTGCC<br>TTTCTGCCGACAAGTAAAACCGCAGATCGGGCAGAAC<br>AACAGCAACATGCTAGT
323C	TGGGATCACTAGCATGTCTG	TGTATGGAGGTTGATGTGCCTGAGAAGATTGCCTTTCT<br>GCCGACAAGTAAAACCGCAGATCGGGCAGAACAAACA<br>GCAACATGCTAGTGAT
323D	TGGGATCACTAGCATGTCTG	TGTATGGAGGTTGATGTGCCTGAGAAGATTGCCTTTCT<br>GCCGACAAGTAAAACCGCAGATCGGGCAGAACAAACA<br>GCAACATGCTAGT
324A	GATTTCTCAATCTCGTCCCA	TCAGGCACATCAACCTCCATACAGGAGAGAACTCTTC<br>AAGTACCATCTGTATTCCGCAGAAAGTGGGCCTGGGA<br>CGAGATTGAGA

324B	GATTTCTCAATCTCGTCCCA	TCAGGCACATCAACCTCCATACAGGAGAGAAACTCTTC<br>AAGTACCATCTGTATTCCGCAGAAAGTGGGCCTGGGA<br>CGAGATTG
324C	GATTTCTCAATCTCGTCCCA	ACATCAACCTCCATACAGGAGAGAAACTCTTCAAGTAC<br>CATCTGTATTCCGCAGAAAGTGGGCCTGGGACGAGAT<br>TGAGA
324D	GATTTCTCAATCTCGTCCCA	ACATCAACCTCCATACAGGAGAGAAACTCTTCAAGTAC<br>CATCTGTATTCCGCAGAAAGTGGGCCTGGGACGAGAT<br>TG
325A	TCTAGTCCATCCCCATTAC	TCAGGCACATCAACCTCCATACAGGAGAGAAACTCTTC<br>AAGTACCATCTGTATTCCGCAGAAAGTGGGCCCGGAA<br>CAAGGTTAAGGAACCTACCGGTGATGGGGGATGGA
325B	TCTAGTCCATCCCCATTAC	TCAGGCACATCAACCTCCATACAGGAGAGAAACTCTTC<br>AAGTACCATCTGTATTCCGCAGAAAGTGGGCCCGGAA<br>CAAGGTTAAGGAACCTACCGGTGATGGGGGAT
325C	TCTAGTCCATCCCCATTAC	ACATCAACCTCCATACAGGAGAGAAACTCTTCAAGTAC<br>CATCTGTATTCCGCAGAAAGTGGGCCCGGAACAAGGT<br>TAAGGAACCTACCGGTGATGGGGGATGGA
325D	TCTAGTCCATCCCCATTAC	ACATCAACCTCCATACAGGAGAGAAACTCTTCAAGTAC<br>CATCTGTATTCCGCAGAAAGTGGGCCCGGAACAAGGT<br>TAAGGAACCTACCGGTGATGGGGGAT
BRD4 screen 2		
327A	TGGGATCACTAGCATGTCTG	TGTATGGAGGTTGATGTGCCTGAGAAGATTGCCTTTCT<br>GCCGACAAGTGAAACCGCAGATCGGGCAGAACAACA<br>GCAACATGCTAGTGATCC
327B	TGGGATCACTAGCATGTCTG	TGTATGGAGGTTGATGTGCCTGAGAAGATTGCCTTTCT<br>GCCGACAAGTGAAACCGCAGATCGGGCAGAACAACA<br>GCAACATGCTAGTGAT
327C	TGGGATCACTAGCATGTCTG	TGTATGGAGGTTGATGTGCCTGAGAAGATTGCCTTTCT<br>GCCGACAAGTGAAACCGCAGATCGGGCAGAACAACA<br>GCAACATGCTAGTG
327D	TGGGATCACTAGCATGTCTG	TGTATGGAGGTTGATGTGCCTGAGAAGATTGCCTTTCT<br>GCCGACAAGTGAAACCGCAGATCGGGCAGAACAACA<br>GCAACATGCTAG
327E	TGGGATCACTAGCATGTCTG	ATGGAGGTTGATGTGCCTGAGAAGATTGCCTTTCTGC<br>CGACAAGTGAAACCGCAGATCGGGCAGAACAACAGC<br>AACATGCTAGTGATCC
327F	TGGGATCACTAGCATGTCTG	ATGGAGGTTGATGTGCCTGAGAAGATTGCCTTTCTGC<br>CGACAAGTGAAACCGCAGATCGGGCAGAACAACAGC<br>AACATGCTAGTGAT
327G	TGGGATCACTAGCATGTCTG	ATGGAGGTTGATGTGCCTGAGAAGATTGCCTTTCTGC<br>CGACAAGTGAAACCGCAGATCGGGCAGAACAACAGC<br>AACATGCTAGTG
327H	TGGGATCACTAGCATGTCTG	ATGGAGGTTGATGTGCCTGAGAAGATTGCCTTTCTGC<br>CGACAAGTGAAACCGCAGATCGGGCAGAACAACAGC<br>AACATGCTAG
327I	TGGGATCACTAGCATGTCTG	GAGGTTGATGTGCCTGAGAAGATTGCCTTTCTGCCGA<br>CAAGTGAAACCGCAGATCGGGCAGAACAACAGCAACA<br>TGCTAGTGATCC
327J	TGGGATCACTAGCATGTCTG	GAGGTTGATGTGCCTGAGAAGATTGCCTTTCTGCCGA<br>CAAGTGAAACCGCAGATCGGGCAGAACAACAGCAACA<br>TGCTAGTGAT
327K	TGGGATCACTAGCATGTCTG	GAGGTTGATGTGCCTGAGAAGATTGCCTTTCTGCCGA<br>CAAGTGAAACCGCAGATCGGGCAGAACAACAGCAACA<br>TGCTAGTG
327L	TGGGATCACTAGCATGTCTG	GAGGTTGATGTGCCTGAGAAGATTGCCTTTCTGCCGA<br>CAAGTGAAACCGCAGATCGGGCAGAACAACAGCAACA<br>TGCTAG
328A	GATTTCTCAATCTCGTCCCA	TCTCAGGCACATCAACCTCCATACAGGAGAGAAACTCT<br>TCAAGTACCATCTGTATTCCGCAGAAAGTGGGCCTGG<br>GACGAGATTGAGAAA

328B	GATTTCTCAATCTCGTCCCA	TCTCAGGCACATCAACCTCCATACAGGAGAGAAACTCT<br>TCAAGTACCATCTGTATTCCGCAGAAAGTGGGCCTGG<br>GACGAGATTGAGA
328C	GATTTCTCAATCTCGTCCCA	TCTCAGGCACATCAACCTCCATACAGGAGAGAAACTCT<br>TCAAGTACCATCTGTATTCCGCAGAAAGTGGGCCTGG<br>GACGAGATTGA
328D	GATTTCTCAATCTCGTCCCA	TCTCAGGCACATCAACCTCCATACAGGAGAGAAACTCT<br>TCAAGTACCATCTGTATTCCGCAGAAAGTGGGCCTGG<br>GACGAGATT
328E	GATTTCTCAATCTCGTCCCA	TCAGGCACATCAACCTCCATACAGGAGAGAAACTCTTC<br>AAGTACCATCTGTATTCCGCAGAAAGTGGGCCTGGGA<br>CGAGATTGAGAAA
328F	GATTTCTCAATCTCGTCCCA	TCAGGCACATCAACCTCCATACAGGAGAGAAACTCTTC<br>AAGTACCATCTGTATTCCGCAGAAAGTGGGCCTGGGA<br>CGAGATTGAGA
328G	GATTTCTCAATCTCGTCCCA	TCAGGCACATCAACCTCCATACAGGAGAGAAACTCTTC<br>AAGTACCATCTGTATTCCGCAGAAAGTGGGCCTGGGA<br>CGAGATTGA
328H	GATTTCTCAATCTCGTCCCA	TCAGGCACATCAACCTCCATACAGGAGAGAAACTCTTC<br>AAGTACCATCTGTATTCCGCAGAAAGTGGGCCTGGGA<br>CGAGATT
328I	GATTTCTCAATCTCGTCCCA	AGGCACATCAACCTCCATACAGGAGAGAAACTCTTCAA<br>GTACCATCTGTATTCCGCAGAAAGTGGGCCTGGGACG<br>AGATTGAGAAA
328J	GATTTCTCAATCTCGTCCCA	AGGCACATCAACCTCCATACAGGAGAGAAACTCTTCAA<br>GTACCATCTGTATTCCGCAGAAAGTGGGCCTGGGACG<br>AGATTGAGA
328K	GATTTCTCAATCTCGTCCCA	AGGCACATCAACCTCCATACAGGAGAGAAACTCTTCAA<br>GTACCATCTGTATTCCGCAGAAAGTGGGCCTGGGACG<br>AGATTGA
328L	GATTTCTCAATCTCGTCCCA	AGGCACATCAACCTCCATACAGGAGAGAAACTCTTCAA<br>GTACCATCTGTATTCCGCAGAAAGTGGGCCTGGGACG<br>AGATT
328M	GATTTCTCAATCTCGTCCCA	GCACATCAACCTCCATACAGGAGAGAAACTCTTCAAGT<br>ACCATCTGTATTCCGCAGAAAGTGGGCCTGGGACGAG<br>ATTGAGAAA
328N	GATTTCTCAATCTCGTCCCA	GCACATCAACCTCCATACAGGAGAGAAACTCTTCAAGT<br>ACCATCTGTATTCCGCAGAAAGTGGGCCTGGGACGAG<br>ATTGAGA
328O	GATTTCTCAATCTCGTCCCA	GCACATCAACCTCCATACAGGAGAGAAACTCTTCAAGT<br>ACCATCTGTATTCCGCAGAAAGTGGGCCTGGGACGAG<br>ATTGA
328P	GATTTCTCAATCTCGTCCCA	GCACATCAACCTCCATACAGGAGAGAAACTCTTCAAGT<br>ACCATCTGTATTCCGCAGAAAGTGGGCCTGGGACGAG<br>ATT
BRD4 single flap edit		
DTN464	GATTTCTCAATCTCGTCCCA	TGCCTGGTGAAGAATGTGATGGGATCACTAGCATGCT<br>CCTGTTGTTCTGCCGATCTGCGGTTTCACTTGTCGG<br>CAGAAAGGCAATCTTCTCAGGCACATCAACCTCCATAC<br>AGGAGAGAAACTCTTCAAGTACCATCTGTATTCTGCGG<br>AGAGCGGGCCTGGGACGAGATTGAGA
DTN487	GTGCCTGGTGAAGAATGTGA	

Table S4. Primers used in this study. Illumina barcodes and UMIs underlined.

Primer	Sequence	Use
JM1079	<u>ACACTCTTTCCCTACACGACGCTCTTCCGATCTNNNNNNNNNNNNNNNN</u> CTCCTGGAGGAGTACGGCT	PLK1-fwd
JM1080	<u>TGGAGTTCAGACGTGTGCTCTTCCGATCTCACCTGCAAGGATGATGCAGC</u>	PLK1-rev
JM1081	<u>ACACTCTTTCCCTACACGACGCTCTTCCGATCTNNNNNNNNNNNNNNNN</u> gctgactgatatctcacgggg	BRD4-fwd
JM1082	<u>TGGAGTTCAGACGTGTGCTCTTCCGATCTTGGTCTGCCTCTTGGGCTTGTTA</u>	BRD4-rev
JM1062	ACACTCTTTCCCTACACG	UMI-fwd PCR1
AB1396	ACAGAGAGAATAACATAAAAAACAGGAAGC	Phage-fwd
AB1792	TAATGGAAACTTCCTCATGAAAAAGTCTTTAG	Phage-rev
JM1103	TCTTCTCTTCCTTTGTAGAGTGCCTGGTGAAGAATGTGATGGGATCACTAGCATGCTTCTCCTGT<br>TTTGCCCCATATGC	SD40 HDR fwd
JM1104	CATCCCCCATTACTGGCAGATTTCTCAATCTCGTGCCAGGCCGCTCTCCGCAGAATACAGGTGA<br>TATTTGAACAGCTTCTCAC	SD40 HDR rev
JM1105	TCTTCTCTTCCTTTGTAGAGTGCCTGGTGAAGAATGTGATGGGATCACTAGCATGGGCGTCCAGG<br>TCGAGACCATTTC	FKBP12 HDR fwd
JM1106	CATCCCCCATTACTGGCAGATTTCTCAATCTCGTGCCAGGCCGCTCTCCGCAGACTCCAATTTA<br>AGTAACTCAACGTCGAAC	FKBP12 HDR rev

Table S5. Sp-Cas9 sgRNAs targeting BRD4. The spacers are underlined. The sgRNAs were synthesized with IDT's Alt-R modifications from Integrated DNA Technologies (IDT).

Spacer 1

UGGGAUCACUAGCAUGUCUGGUUUAAGAGCUAUGCUGGAAACAGCAUAGCAAGUUUAAAUAAGCUAGUCCGUUAUCAACUUGAAAAAGUGGCACCGAGUCGGUGCUUUU

Spacer 2

GAUUUCUCAAUCUCGUCCCAGUUUAAGAGCUAUGCUGGAAACAGCAUAGCAAGUUUAAAUAAGCUAGUCCGUUAUCAACUUGAAAAAGUGGCACCGAGUCGGUGCUUUU

Table S6. Cryo-EM data collection, refinement, and validation statistics.

	DDB1•CRBN•<br>pomalidomide•SD40<br>8TNP<br>EMD-41423 &<br>EMD-41777	DDB1•CRBN•PT-<br>179•SD40, conf 1<br>8TNQ<br>EMD-41424 &<br>EMD-41778	DDB1•CRBN•PT-<br>179•SD40, conf 2<br>8TNR<br>EMD-41425 &<br>EMD-41779
Data collection and processing			
Microscope	Thermo Fisher Scientific<br>Talos Arctica	Thermo Fisher Scientific Titan Krios	
Voltage (kV)	200	300	
Camera	Gatan K3	Gatan K3 BioQuantum	
Magnification (x)	36,000	105,000	
Pixel size at detector (Å/pixel)	1.1	0.83	
Total electron exposure (e ⁻ /Å ²)	53.8	52.6	
Exposure rate (e ⁻ /pixel/s)	13.0	14.5	
Frames collected during exposure (no.)	50	50	
Defocus range (µm)	-0.8 – -2.0	-0.8 – -2.0	
Automation software	SerialEM 4.1b	SerialEM 4.1b	
Energy filter slit width (eV)	-	20	
Micrographs collected (no.)	2,524	16,340	
Micrographs used (no.)	1,212	12,642	
Total extracted particles (no.)	1,254,659	6,798,113	
For each reconstruction			
Refined particles (no.)	561,074	1,844,380	1,844,380
Final particles (no.)	53,025	289,691	244,713
Point-group	C1	C1	C1
Resolution (global, Å)			
FSC 0.5 (unmasked/masked)	8.3/3.8	4.0/2.8	4.2/2.9
FSC 0.143 (unmasked/masked)	4.4/3.3	3.3/2.4	3.4/2.5
Resolution range (local, Å)	2.8 – 8.1	2.1 – 6.6	2.3 – 6.7
Resolution range due to anisotropy (Å)	3.3 – 4.0	2.3 – 2.9	2.4 – 3.1
3D FSC sphericity	0.968	0.959	0.953
Map sharpening <i>B</i> factor (Å ²)	102	70	71
Map sharpening methods	Global B-factor<br>sharpening (for model<br>refinement and<br>validation)<br>Local resolution<br>sharpening and<br>DeepEMhancer (for<br>visualization and model<br>building)	Unsharpened (for<br>model refinement and<br>validation)<br>Local resolution<br>sharpening and<br>DeepEMhancer (for<br>visualization and<br>model building)	Unsharpened (for<br>model refinement and<br>validation)<br>Local resolution<br>sharpening and<br>DeepEMhancer (for<br>visualization and<br>model building)
Model composition			
Protein	1159	1208	1216
Ligands	3	3	3
Model refinement			
Refinement package	phenix.real_space_refine	phenix.real_space_refine	phenix.real_space_refine
- Real or reciprocal space	Real	Real	Real
- Resolution cutoff (Å)	3.3	2.4	2.5

Model-Map scores			
- CC	0.67	0.82	0.84
- Average FSC (Å, FSC 0.5)	3.6	2.6	2.8
<i>B</i> factors (Å ²)			
Protein residues	129.0	126.0	113.1
Ligands	152.3	148.2	129.3
R.m.s. deviations from ideal values			
Bond lengths (Å)	0.005	0.004	0.004
Bond angles (°)	0.577	0.514	0.524
**Validation**			
MolProbity score	1.66	1.46	1.81
CaBLAM outliers (%)	2.60	1.46	1.96
Clashscore	7.14	4.52	6.66
Rotamer outliers (%)	0	0.65	1.58
C _β outliers (%)	0	0	0
EMRinger score	2.43	4.41	3.66
Ramachandran plot			
Favored (%)	96.04	96.46	95.74
Allowed (%)	3.96	3.54	4.26
Disallowed (%)	0	0	0

Table S7. Residue pair energy between residue 18 of SD40/S40^{F18Q} and PT-179 calculated by Rosetta (non-zero terms). Full tables available online (112).

Residue type	fa_atr	fa_rep	fa_sol	lk_ball	lk_ball_iso	fa_elec	total
Phenylalanine	-4.359	0.408	1.103	0.499	-0.485	0.146	-2.688
Glutamine	-3.336	0.458	1.927	0.334	-0.543	-0.218	-1.378

Key:

fa_atr: attractive component of Lennard-Jones potential between atoms in different residues

fa_rep: repulsive component of Lennard-Jones potential between atoms in different residues

fa_sol: Gaussian exclusion implicit solvation energy

lk_ball: orientation-dependent solvation of polar atoms

lk_ball_iso: isotropic contribution to the solvation energy

fa_elec: interaction between non-bonded charged atoms in different residues

total: weighted sum of the individual energy terms

Movie S1. CRBN–DDB1 interface flexibility (provided as a separate file)

Movie looping through 20 clusters from 3D variability illustrating the flexibility at the CRBN–DDB1 interface in the DDB1^{ΔBPB}•CRBN•PT-179•SD40 data set.

Data S1. Global_proteomics_limma_output.xlsx (provided as a separate file)

Hit lists depicting the fold-change in relative abundance comparing treatment to DMSO control determined using global proteomics. Significant changes were assessed by moderated t-test as implemented in the limma package (87).
